# Reassessment of *Annamocarya sinesis* (*Carya sinensis*) Taxonomy through Concatenation and Coalescence Phylogenetic Analysis

**DOI:** 10.3390/plants11010052

**Published:** 2021-12-24

**Authors:** Jie Luo, Junhao Chen, Wenlei Guo, Zhengfu Yang, Kean-Jin Lim, Zhengjia Wang

**Affiliations:** 1 State Key Laboratory of Subtropical Silviculture, College of Forestry and Biotechnology, Zhejiang A&F University, Lin’an, Hangzhou 311300, China; luojie0103@outlook.com (J.L.); junhao.chen.1@slu.edu (J.C.); guowenlei@ioz.ac.cn (W.G.); zafuyzf@163.com (Z.Y.); 2 Department of Biology, Saint Louis University, St. Louis, MO 63104, USA; 3 State Key Laboratory of Integrated Management of Pest Insects and Rodents, Institute of Zoology, Chinese Academy of Sciences, Beijing 100101, China

**Keywords:** *Carya sinensis*, *Annamocarya sinensis*, unique gene, concatenation, coalescence, phylogeny

## Abstract

Due to its peculiar morphological characteristics, there is dispute as to whether the genus of *Annamocarya sinensis*, a species of Juglandaceae, is *Annamocarya* or *Carya*. Most morphologists believe it should be distinguished from the *Carya* genus while genomicists suggest that *A. sinensis* belongs to the *Carya* genus. To explore the taxonomic status of *A. sinensis* using chloroplast genes, we collected chloroplast genomes of 16 plant species and assembled chloroplast genomes of 10 unpublished *Carya* species. We analyzed all 26 species’ chloroplast genomes through two analytical approaches (concatenation and coalescence), using the entire and unique chloroplast coding sequence (CDS) and entire and protein sequences. Our results indicate that the analysis of the CDS and protein sequences or unique CDS and unique protein sequence of chloroplast genomes shows that *A. sinensis* indeed belongs to the *Carya* genus. In addition, our analysis shows that, compared to single chloroplast genes, the phylogeny trees constructed using numerous genes showed higher consistency. Moreover, the phylogenetic analysis calculated with the coalescence method and unique gene sequences was more robust than that done with the concatenation method, particularly for analyzing phylogenetically controversial species. Through the analysis, our results concluded that *A. sinensis* should be called *C. sinensis*.

## 1. Introduction

**Figure 1:**
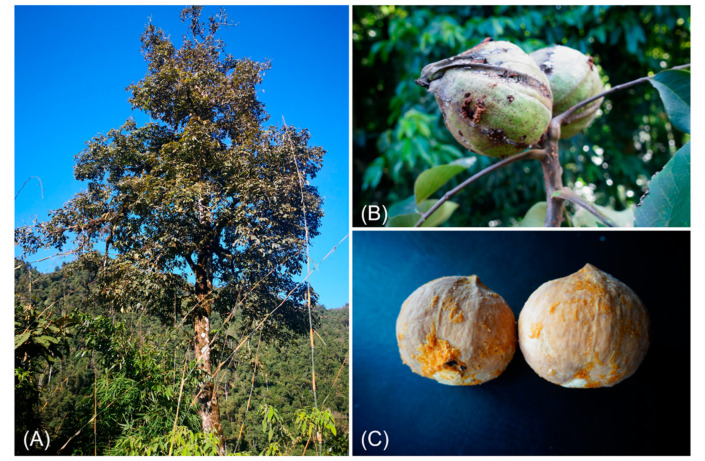
*Annamocarya sinensis* (*Carya sinensis*). (**A**) Tree; (**B**) Fruits in tree; (**C**) Fruits without husk (Photos provided by Weibang Sun, Kunming Institute of Botany, China).

*Annamocarya sinensis*, controversially called *Carya sinensis*, is widely distributed in southern China and northern Vietnam. As a deciduous tree, it is generally about 30 m tall, has a 125-cm diameter trunk, and has grayish bark with alternate leaves (Figure 1) [1]. As the individual number of *A. sinensis* has reduced and its distribution narrowed, it is now a Class II endangered species in China [2]. It belongs to the Juglandaceae family, but its genus is still controversial; while most plant scientists classify it as *Annamocarya*, some classify it as *Carya*. This dispute has been caused by different taxonomic approaches, especially in morphology and evolutionary genomics.

Dode (1912) first described *A. sinensis* as *C. sinensis* based on limited herbarium samples, seed specimens without leaves, and flower samples [3]. Hence, his classification was controversial [4]. Chevalier (1941) [5] described the morphological characteristics of the leaves and nuts and concluded that it belonged to a new, single genus, *Annamocarya*. Nevertheless, Manning and Hjelmqvist (1951) [4] questioned whether, given that *Carya* and it shared plenty of similar morphological characteristics, it was necessary to describe this uncertain species as a single genus. Most morphologists believe that it is distinguished from *Carya* based on several morphological characteristics. For example, the leaves of *A. sinensis* are smooth, while *Carya* has serrated leaves; *A. sinensis* has five to eight catkins per bundle, whereas *Carya* has only three. The staminate bract of *A. sinensis* have three or more vascular strands, but only one in *Carya* [6]. And Leroy (1955) [7] proposed that the characters of the shell might be an important reason to distinguish it from *Carya*. Grauke et al. (1991) [3] compared the differences between *Carya* and *Annamocarya* in terms of tree shape, leaves, flowers, fruit shape, and other samples collected from Cuc Phuong National Forest in Vietnam. They concluded that the separate genus *Annamocarya* was necessary. As more morphological pieces of evidence became available, the genus *Annamocarya* was adopted over a long time.

In the 21st century, with the rapid development of sequencing technology, researchers have increasingly used gene sequences to study the evolutionary history of species with a phylogenomic approach. Manos and Stone (2001) [6] analyzed the phylogeny of Juglandaceae by using the internal transcribed spacer (ITS) and a few chloroplast genes. Their results suggest that *A. sinensis* is closely related to the *Carya* genus. In contrast, Li et al. (2004) [8] separated *A. sinensis* and *Carya* on different branches of infrafamilial relationships of the order Fagales based on six genes (*atpB*, *matK*, *matR*, *rbcL*, *trnL-F* and 18S rDNA). Manos et al. (2007) [9] investigated the evolution of Juglandaceae by integrating fossils and nucleic sequence evidence. Several results in their study suggest that *A. sinensis* should still be grouped within the *Carya* genus. Zhang et al. (2013) [10] used six plastid fragments (*matK*, *rbcL-atpB*, *rpoC1*, *rps16*, *trnH-psbA*, and *trnL-F*) and nuclear markers (ITS and *phyA*) in their study; the results suggest that *A. sinensis* should be clustered within the *Carya* genus. It seems, based on these examples, that sequence analysis is likely to classify *A. sinensis* in the *Carya* genus.

Recently, nuclear and organelle genes have commonly been used to analyze the phylogeny of species. Among them, chloroplast genome data has been utilized due to its low evolution rate and stable in variation [11]. It has been widely used to study phylogeny, chloroplast inheritance, domestication history, and adaptative evolution [12,13]. For example, Wu et al. (2020) [14] constructed a phylogenetic analysis of *Chrysosplenium* based on the whole chloroplast genome. They conclude that *Chrysosplenium* could be divided into two subgroups: those having alternate leaves or opposite leaves. Summarizing empirical studies that studied the phylogeny of *Carya* based on chloroplast genes, the similarity among them is that only a few chloroplast genes or fragments were utilized [6,9,10]. Researchers have found that phylogeny trees built from individual genes may show discordance on the species tree [15,16]. Furthermore, as the number of gene trees increases, they may converge in probability to the true species tree [17]. Hence, phylogenetic trees constructed entirely of chloroplast genome genes may be reliable.

**Figure 2:**
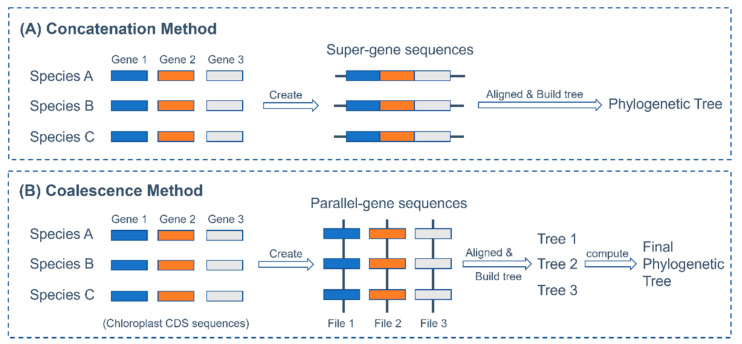
Methods of concatenation and coalescence. (**A**) Concatenation method joins all gene sequences of each species into each super-gene sequence. These super-gene sequences are then used in aligning and building phylogeny trees; (**B**) Coalescence method extracts the same gene sequences from all species and merges them into each single parallel-gene sequence file. Then all parallel-gene sequence files are aligned separately to build trees. Finally, all those trees are computed to get the final tree.

Since the development of next-generation sequencing and computational phylogenomics, the reconstruction of angiosperm phylogenies from multiple genes has relied upon concatenation methods [18]. The concatenation method concatenates multiple gene sequences of each species and treats them as one alignment to generate a phylogenetic tree (Figure 2A). As the number of subsampled nuclear genes increases, the result of concatenation analyses becomes dependable [18]. However, a simulation study by Kubatko and Degnan (2007) [19] shows that such approaches could lead to misleading phylogenetic calculations. They demonstrated that using concatenated data, species tree estimation performed deficiently and was statistically inconsistent even under stable population size without selection or population stratification. In addition to concatenation method, recent studies have also taken advantage of coalescence method for phylogenetic analysis [20,21].This method first computes gene trees by the individual gene sequences and then aggregates all gene trees to obtain the final species tree (Figure 2B) [15]. Goncalves and colleagues [22] conducted a phylogenetic analysis of 78 plastid genes, and their results showed that the phylogenetic tree inferred by the coalescence method was reliable. Xi et al. (2013) [23] propose that the coalescence method might reduce the potential deleterious effect of elevated substitution rates in phylogenomic analyses. To test this, Xi et al. (2014) [18] used the two methods to analyze the phylogeny of *Amborella* within 45 seed plants and found that fast-evolving sites likely disrupt the concatenation method, while the coalescence method appears more robust in response to elevated substitution rates.

In this work, we first assembled 10 chloroplast genomes of the *Carya* species for observing the phylogenetic relationship between *A. sinensis* and *Carya*. We determined the true phylogenetic location of *A. sinensis* by comparing and evaluating the concatenation and coalescence methods with the chloroplast genome of 26 species. Our results indicate that *A. sinensis* was clustered within the *Carya* genus. Hence, we conclude that *Annamocarya sinesis* should be referred to as *Carya sinensis*. Moreover, the phylogeny results constructed by the coalescence method are more robust than those by the concatenation method.

## 2. Results

We analyze the phylogenetic position of *A. sinensis* using the genes of the chloroplast genome. A total of 14 species belonging to the *Carya* genus of Eastern Asia and North America were included in this work (four species of Eastern Asia include *C. cathayensis*, *C. dabieshanensis*, *C. hunanensis*, and *C. kweichowensis*; 10 species of North America include *C. aquatica*, *C. cordiformis*, *C. glabra*, *C. illinoinensis*, *C. laciniosa*, *C. myristiciformis*, *C. ovata*, *C. palmeri*, *C. texana*, and *C. tomentosa*) [10]. The CDS and protein sequences of all 26 species of six genera (*Betula*, *Carya*, *Cyclocarya*, *Juglans*, *Platycarya*, and *Pterocarya*) were used for the analysis.

### 2.1. Assemblies and Annotations of Chloroplast Genomes

**Figure 3:**
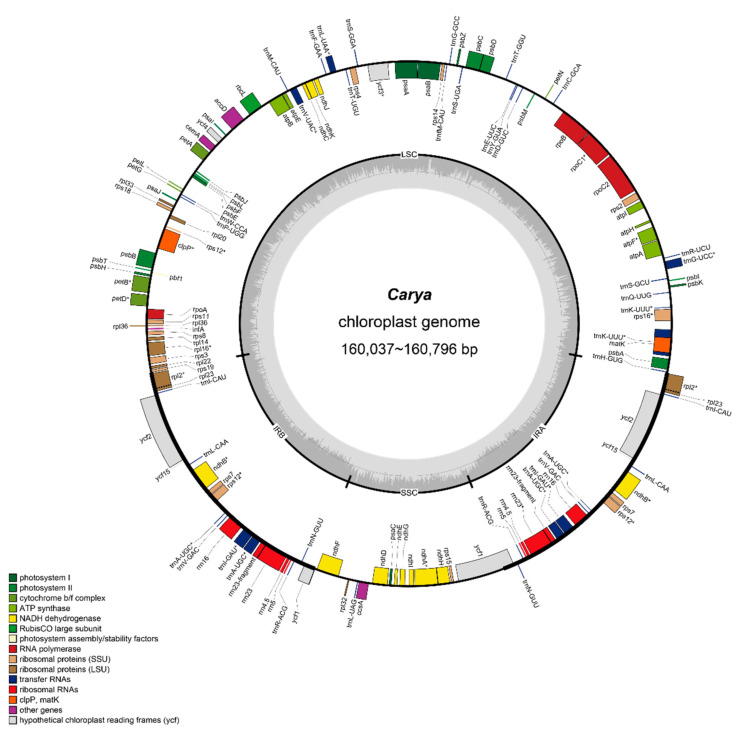
The circular chloroplast genome map of *Carya* (take *C. cordiformis* as reference graph). The genes shown inside and outside of the circle are transcribed in clockwise and counterclockwise directions, respectively. Genes from different functional groups are shown in different colors. The thick dark lines in inner circle show the extent of the Inverted repeats (IRA and IRB) separating the Large Single-Copy (LSC) and the Small Single-Copy (SSC) regions. The gray ring represents the GC content. The circular chloroplast genome maps of 10 species were show in Figure S1.

We first obtained the raw sequencing data of 10 species of the *Carya* genus (Table S1). Then, we assembled the 10 complete closed-loop chloroplast genomes of these species (Figure 3 and Figure S1) for the analysis below. From the assemblies, we learned that the average size of the 10 species was about 160 kb (Table 1). The chloroplast genome of *C. cordiformis* was the longest at 160,796 bp, and the shortest one was *C. dabieshanensis* at 160,037 bp; the difference between the two was 759 bp. The GC content of the assembled 10 chloroplast genomes was similar, around 36%.

We also obtained the chloroplast genomes of 16 species of six genera from other database, used GeSeq [24] to reannotate the chloroplast genomes of all 26 species to standardize the annotations previously generated by different annotation tools. The chloroplast genomes of the *Carya* genus contained an average of 79 coding genes, suggesting that they were highly conserved (Table 2). The *Cyclocarya* and *Pterocarya* genera contained the most coding genes. The CDS GC content of all species was approximately 37.22%, with little difference. The uniformly reannotated chloroplast genomes were used for downstream analysis.

### 2.2. Unique Genes of the Chloroplast Genomes

After uniformly reannotating the chloroplast genomes, we used Orthofinder to identify 50 unique genes (Table 3) based on these chloroplast protein sequences. All 50 unique genes were single-copy genes in the chloroplast genome of all 26 species. Most of them were related to photoreaction and ribosome function. And according to these unique genes, we then extracted 50 unique CDS sequences and 50 unique protein sequences for downstream analysis.

**Figure 4:**
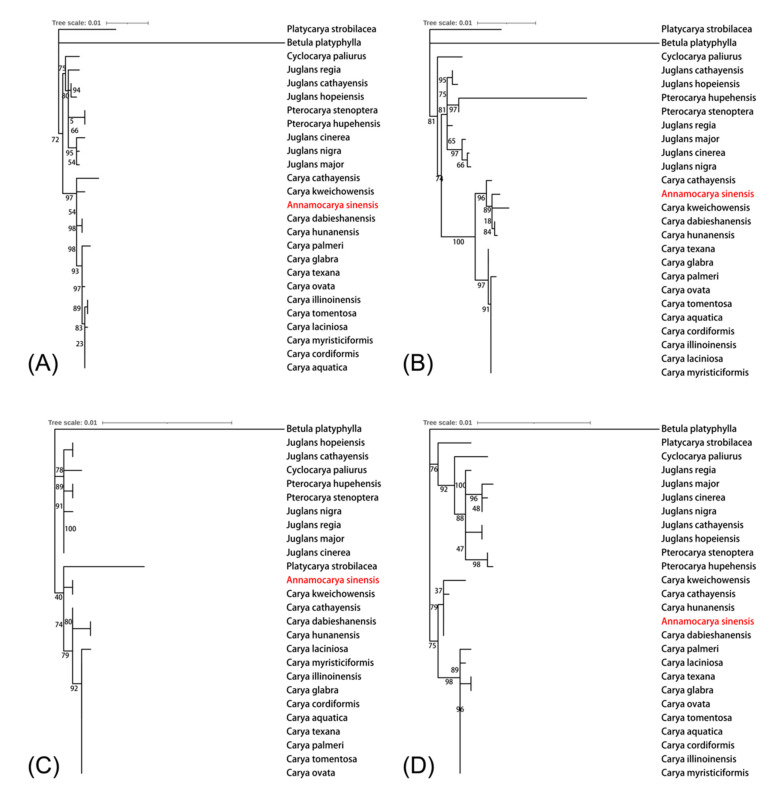
Phylogenetic trees constructed by unique chloroplast gene. (**A**) *matK*; (**B**) *ndhF*; (**C**) *rbcL*; (**D**) *rpoC1*.

After extracting 50 unique genes, we constructed the phylogenetic trees using each individual gene. However, most of the 50 chloroplast gene trees (Figure S2) showed chaotic phylogenetic relationships. For example, *matK*, *ndhF*, *rbcL* and *rpoC1* genes (Figure 4), which are commonly used to construct phylogenetic trees, indicated that species of the genus *Juglans* failed to group together. And even if we rerooted *Betula platyphylla* as an outgroup, it cannot be separated from *Platycarya strobilaceaat* some point (Figure 4A,B). The *rpoC1* gene phylogenetic tree was the only tree correctly classified all genera and clustered pecans into East Asia and North America. Most phylogenetic trees showed the analyzed species were confusingly classified into the wrong genera. The evolutionary relationship of these results was unstable and inconsistent. Hence, we utilize the following two methods to achieve a certain degree of reliability.

### 2.3. Phylogenetic Analysis Based on the Concatenation Method

**Figure 5:**
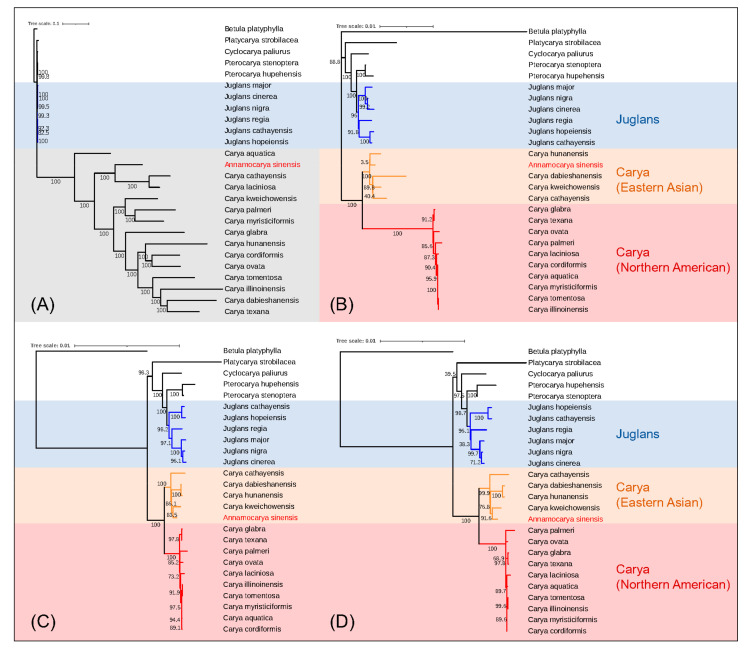
Phylogeny analysis based on the concatenation method. Visualized using ITOL. (**A**) Analyzed with entire CDS sequences; (**B**) Analyzed with entire protein sequences; (**C**) Analyzed with unique CDS sequences; (**D**) Analyzed with unique protein sequences.

We collected whole CDS sequences and protein sequences (79–89 genes in each) from the reannotated chloroplast genomes of all 26 species. Then, we extracted the unique CDS sequences and unique protein sequences (50 genes in each) according to the analysis result of Orthofinder. The four phylogenetic trees of entire CDS sequences, entire protein sequences, unique CDS sequences, and unique protein sequences constructed by the concatenation method are shown in Figure 5A–D. Impressively, we noticed that the out-of-group branches (genera of *Betula*, *Cyclocarya*, *Juglans*, *Platycarya*, and *Ptercocarya*) in these four trees were clustered in the same clades. Species in genera such as *Juglans* and *Pterocarya* were always clustered together. In all the results, as we hypothesized, *A. sinensis* was classified into the *Carya* genus branch. The results (Figure 5B–D) clustered all *Carya* into Eastern Asian and North American subgroups well except for the phylogenetic tree using the entire CDS sequences (Figure 5A), which showed low bootstrap credibility. Interestingly, compared to the results of entire gene sequences, the two results based on unique genes (Figure 5C,D) showed more similarity, indicating that the phylogeny analysis using unique genes was stable. Moreover, *A. sinensis* was close with *C. kweichowensis* in these two result trees (Figure 5C,D). The results are in accordance with the geographical distribution of these two species [2,25].

### 2.4. Phylogenetic Analysis Based on Coalescence Method

**Figure 6:**
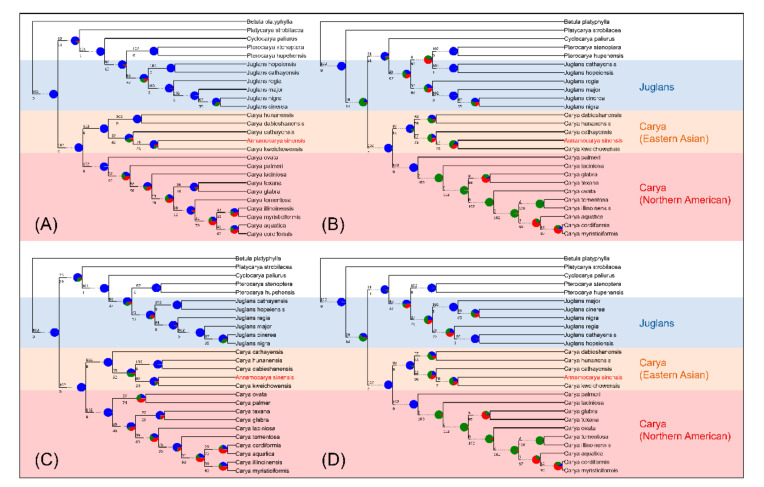
Phylogeny analysis based on the coalescence method. (**A**) Analyzed with entire CDS sequences, final normalized quartet score: 0.954; (**B**) Analyzed with entire protein sequences, final normalized quartet score: 0.804; (**C**) Analyzed with unique CDS sequences, final normalized quartet score: 0.948; (**D**) Analyzed with unique protein sequences, final normalized quartet score: 0.807. The pie chart at each node showed the support of gene trees as the following: concordant with gene trees (blue), most common conflict bipartition (green), other conflicting bipartitions (red) and unsupported with gene trees (grey).

Subsequently, we again analyzed the phylogenetics of *A. sinensis* based on the coalescence method with entire CDS sequences, entire protein sequences, unique CDS sequences, and unique protein sequences. The resulting four phylogenetic trees are shown in Figure 6. It should be noted that these coalescence results were computed by ASTRAL, which used a quartet score to score species trees instead of bootstrap. The closer the score is to 1, the more credible the tree is. Scores of the four results were all greater than 0.8, denoting credibility. As was conjectured, the out-of-group branches (the five genera mentioned before) of the trees were clustered in correct phylogenetic positions with high confidence support (blue pie charts, Figure 6). Similarly, *A. sinensis* in all results was consistently classified into the Eastern Asian *Carya* genus branch, especially close with *C. kweichowensis*. The conflict analysis of CDSs (Figure 6A,C) showed more concordant with gene trees than protein sequences (Figure 6B,D). The clades of Northern American *Carya*, on the other hand, showed much common conflicts (green pie charts, Figure 6B,D), perhaps because of the degeneracy among these close species. Surprisingly, all leaves of phylogenetic tree constructed by the coalescence method, either using entire-gene or unique-gene sequences, were almost identical (Figure 6).

## 3. Discussion

The taxonomic status of *A. sinensis* has long been debated. Morphologists distinguished it from the *Carya* genus by morphological features such as leaves, bunches, bracts, and nut shells. However, the accuracy of sequencing technologies nowadays makes molecular phylogeny being more common and credible [26]. Following the release of raw sequencing data (Table S1) for some species of *Carya* [27], we have assembled and annotated the chloroplast genomes of 10 species of *Carya* that have not yet been published. Among them, nine North American species were assembled using *C. illinoinensis* (MH909600) as a reference chloroplast genome, and only *C. dabieshanensis* (Eastern Asian) was assembled using *C. cathayensis* (NC_046572) as a reference chloroplast genome (Table S1). The chloroplast genomes we assembled (Table 1) using GetOrganelle were of high quality; both the size of the LSC/SSC/IR region and the GC content were similar to the published chloroplast genomes of other *Carya* species [28]. All these characteristics of Juglandaceae chloroplast genomes show stability in variation and low evolutionary rate [11].

*Juglans* has been divided into a four-section classification (*Cardiocaryon*, *Dioscaryon*, *Rhysocaryon*, *Trachycaryon*) [29]. Six species of these four sections were selected as the reference taxa in our evolutionary trees. As with the eight phylogenetic trees mentioned in Figure 5 and Figure 6, we observed that all outgroup taxa were clustered into proper phylogenetic positions. In particular, the *Carya* genus were categorized correctly into Eastern Asia and North America in both methods, which means that the analytical strategies we employed were reliable. Our results show that *A. sinensis* does have a close phylogenetic relationship with the *Carya* species, rather than separating itself into an independent clade. Our results also show that *A. sinensis* close to *C. kweichowensis* in the *Carya* clade. Both of these two species are scattered in southwestern China with an endemic distribution area [2,25]. The phylogenetic relationship of our results was highly consistent with geographical distribution, indicating that *A. sinensis* is reliably clustered in the *Carya* genus, in the Eastern Asian branch, whether based on molecular or geographical evidence. We conclude that *A. sinensis* should belong in *Carya* rather than divided into a single genus. Hence, it should be referred to as *Carya sinensis*.

The phylogenetic trees created by a single gene indicate that most of these single-gene trees cannot classify all species into the correct genera. It reveals that the evolutionary tree created by this single chloroplast gene may be unreliable. We also analyzed the phylogeny of the entire chloroplast genome of 26 species (Figure S3). However, the resulting taxa did not conform to the geographical distribution of these species. Therefore, it is necessary to adopt appropriate methods to make the evolutionary results stable and reliable. The two main approaches we put used in this study were concatenation and coalescence. Compared with the traditional concatenation method, the coalescence method, a new computer method, was more effective and robust when applied to phylogeny analysis [20]. The phylogenetic trees created by ASTRAL includes the conflicts of each node (displayed as pie charts), indicating that coalescence results were concordant with highly supported gene trees (blue pie charts, Figure 6). However, some nodes still showed conflicts (green or red pie charts), indicating that the gene trees which were inconsonant with species trees were still not negligible, especially in the American *Carya* genus branch. Conflicts caused by individual genes trees may be related to several reasons. Some plastid genes are multiple in one species while haploid in another species or the inverted repeats of genes were confused when extracting them and constructing phylogenetic trees. The correction of such conflicts between genes trees and species trees probably should be investigated in further study. The phylogenetic relationship between *Carya* species computed with the coalescence method (Figure 6) showed high similarity. In contrast, these results analyzed by the concatenation method showed some heterogeneity (Figure 5). In addition to the entire chloroplast genes, we also combined unique genes for analysis. The utilization of combined unique genes may provide a complete selection of independent characters for plant phylogenetic analysis [30]. Our results showed that *A. sinensis* was always next to *C. kweichowensis* in the *Carya* clade when using the combined unique gene sequences for the analysis (Figure 5C,D and Figure 6C,D), indicating that the combined unique gene sequences were stable and consistent for the phylogenetic analysis. The very low non-conservative percentage of the sequence alignment (Figure S4) of the chloroplast genes of *A. sinensis* and *C. kweichowensis* suggested that this approach is reasonable. All these results conclude that the coalescence method using unique genes is recommended when computing phylogenetic species trees, especially for the analysis of phylogenetic controversial or close related species.

Even if molecular analysis evidence seems more accurate in classification, we cannot ignore the practicality and reliability of morphological evidence. Compared with molecular analysis, morphological analysis is serviceable to identify plants in field studies. With permitted conditions, by comparing the molecular and morphological analysis of closely related species, it may be possible to find out the reasons for the differences between them and perhaps prove the correct classification of these species better.

## 4. Materials and Methods

### 4.1. Data Sets

In this work, a total of 26 chloroplast genomes in five genera of Juglandaceae (*Carya*, *Cyclocarya*, *Juglans*, *Platycarya*, and *Pterocarya*) and *Betula platyphylla* were used for phylogeny analysis. Among them, 15 complete chloroplast genomes of Juglandaceae and *Betula platyphylla* were obtained from National Center for Biotechnology Information (NCBI, https://www.ncbi.nlm.nih.gov/, accessed on 1 March 2021; Table S2). The raw sequencing data of another 10 species of *Carya* (Table S1) [27] whose complete chloroplast genome had not been disclosed were downloaded and assembled. The assembled chloroplast genome of these 10 species was then uploaded to NCBI (Table S3). The chloroplast genome of *C. illinoinensis* (cultivar Pawnee) was sequenced and assembled by our lab and can be downloaded by the accession GWHBGBH00000000 at National Genomics Data Center (https://ngdc.cncb.ac.cn/, accessed on 1 November 2021) [31,32].

### 4.2. Chloroplast Genomes Assembly and Gene Annotation

The quality of raw sequencing data (raw reads) mentioned above was assessed using fastp v0.20.1 [33] with default parameters. After the quality assessment, the reads were mapped against the chloroplast genome of *C. illinoinensis* cultivar 87MX3-2.11 (MH909600) using BWA-MEM 0.7.17 [34] to obtain clean chloroplast reads. The SAM mapping results were then transformed to BAM format by SAMtools v1.9 [35,36] for assembly. The complete closed-loop chloroplast genome of each species was assembled using GetOrganelle v1.7.137 [37] with particular reference chloroplast genomes (Table S1). These assembled chloroplast genomes were annotated by the GeSeq tool [24] with *C. illinoinensis* (MH909600) as the reference genome.

### 4.3. Analysis of Unique Chloroplast Genes

We first used GeSeq to uniformly reannotate the chloroplast genomes of all 26 species to standardize the annotations previously generated by different annotation tools. Then the genomes’ protein sequences were subjected to Orthofinder [38,39] analysis with default parameters to obtain the single-copy genes (unique genes). Then both the protein and nucleotide sequences of these unique genes were extracted and prepared for subsequent phylogeny analysis.

The alignment of unique protein and nucleotide sequence files was created with L-INS-I parameters using MAFFT v7.271 [40,41]. Next, these alignment results were then used to construct maximum likelihood phylogenetic trees using IQtree [42] with K3Pu+F as the best fit model and 500 bootstraps.

### 4.4. Phylogeny Analysis Based on Concatenation Method

The entire chloroplast coding sequence (CDS) and protein sequences (entire-genes) of each species (Table 2) were concatenated to make super sequences (Figure 2A) using in-house Linux scripts. The super-entire-CDSs and super-entire-protein sequences of all the chloroplast genomes were acquired. Then, the unique CDS and protein sequences (unique-genes) of all species were concatenated into super-unique-CDSs and super-unique-protein sequences. The multiple alignment of all entire-genes and unique-genes of the super sequences were individually constructed using MAFFT v7.271 with FFT-NS-2 parameters. The phylogenetic tree of the concatenation method was obtained using FastTree [43,44] with the Jukes-Cantor model. Finally, these phylogenetic trees were visualized using ITOL [45].

### 4.5. Phylogeny Analysis Based on Coalescence Method

All coding gene sequences of chloroplast genomes of 26 species were extracted using Seqkit [46]. After gathering common coding gene sequences into common files, a total of 73 specific gene sequence files were acquired (Table S4). These 73 CDS and protein sequences were used to contract the parallel-entire-genes phylogeny trees (Figure 2B). Similarly, the alignment of each specific sequence file was also created using MAFFT v7.271 and constructed phylogenetic trees using IQtree with the same parameters as described in unique chloroplast genes above. All parallel-genes phylogeny trees generated by IQtree were first merged into one file using ASTRAL with the “-b” parameter. And the output (102 trees in default) was then computed to the final species trees using ASTRAL again. The conflicts of nodes of species trees were analyzed using PhyParts [47]. The visualization of trees with pie charts were drawn using PhypartsPieChart (https://github.com/mossmatters/phyloscripts/tree/master/phypartspiecharts, accessed on 7 November 2021).

## 5. Conclusions

The phylogenetic trees created using a single unique gene are unreliable. The concatenation and coalescence methods possibly are appropriate approaches to study the phylogeny of close species. Compared to the concatenation method, the phylogenetic trees constructed using the coalescence method shows stability and reliability. We collected 16 chloroplast genomes and assembled 10 chloroplast genomes of *Carya* species to analyze the phylogeny of *A. sinensis*. Our analysis of the chloroplast genomes with concatenation and coalescence methods showed that *A. sinensis* is clustered into the *Carya* genus. Our results concluded that *A. sinensis*, therefore, should be called *C. sinensis*.

## Figures and Tables

**Table 1:** The assembled ten chloroplast genome structure of 10 *Carya* species.

Species	LSC	SSC	IR	Total Length	GC Content(%)
*Carya aquatica*	89,966	18,791	26,003	160,763	36.16
*Carya cordiformis*	89,992	18,798	26,003	160,796	36.15
*Carya dabieshanensis*	89,508	18,861	25,834	160,037	36.20
*Carya glabra*	89,888	18,786	25,989	160,652	36.18
*Carya laciniosa*	89,927	18,842	26,001	160,771	36.17
*Carya myristiciformis*	89,990	18,792	26,003	160,788	36.15
*Carya ovata*	89,930	18,809	25,994	160,727	36.17
*Carya palmeri*	89,818	18,778	26,004	160,604	36.18
*Carya texana*	89,964	18,793	25,994	160,745	36.17
*Carya tomentosa*	89,988	18,792	26,002	160,784	36.16

**Table 2:** Chloroplast genome features of 26 species.

Species	Genome Size (bp)	Coding Gene Number	tRNA Genes	rRNA Genes	CDS Total Length (bp)	CDS GC Content (%)
*A. sinensis*	158,484	79	35	8	68,261	37.26
*B. platyphylla*	160,518	84	37	8	78,972	37.43
*C. aquatica*	160,763	79	37	8	68,673	37.24
*C. cathayensis*	160,666	80	36	8	69,595	37.22
*C. cordiformis*	160,796	79	37	8	68,673	37.24
*C. dabieshanensis*	160,037	80	37	8	69,250	37.24
*C. glabra*	160,652	79	37	8	68,674	37.25
*C. hunanensis*	160,397	80	36	8	69,449	37.21
*C. illinoinensis*	160,819	79	37	8	68,673	37.25
*C. kweichowensis*	175,313	79	38	8	68,314	37.27
*C. laciniosa*	160,771	79	37	8	68,672	37.26
*C. myristiciformis*	160,788	79	37	8	68,673	37.24
*C. ovata*	160,727	79	37	8	68,680	37.26
*C. palmeri*	160,604	79	37	8	68,673	37.25
*C. texana*	160,745	79	37	8	68,674	37.25
*C. tomentosa*	160,784	79	37	8	68,673	37.25
*Cy. paliurus*	160,562	89	40	8	81,015	37.19
*J. cathayensis*	159,730	87	40	8	80,331	37.26
*J. cinerea*	160,288	84	37	8	78,333	37.26
*J. hopeiensis*	159,714	86	40	8	80,259	37.27
*J. major*	160,276	83	37	8	77,970	37.2
*J. nigra*	160,274	83	37	8	77,958	37.21
*J. regia*	160,370	83	37	8	77,979	37.2
*P. strobilacea*	160,994	85	36	8	78,915	37.18
*Pt. hupehensis*	159,770	89	40	8	81,315	37.2
*Pt. stenoptera*	160,202	89	40	8	81,021	37.23

**Table 3:** A total of 50 unique genes existed in 26 chloroplast genomes.

Fuctional Groups	Name of Genes
*Ribosomal protein small subunit*	*rps2*, *rps3*, *rps4*, *rps8*, *rps11*, *rps14*, *rps18*
*Ribosomal protein large subunit*	*rpl14*, *rpl20*, *rpl22*, *rpl32*, *rpl33*
*Subunits of RNA polymerase*	*rpoA*, *rpoB*, *rpoC1*, *rpoC2*
*Photosystcm I*	*psaA*, *psaB*, *psaI*, *psaJ*
*Photosystem II*	*psbA*, *psbC*, *psbD*, *psbE*, *psbH*, *psbJ*, *psbK*, *psbL*, *psbM*, *psbT*
*Cythochrome b/f complex*	*petA*, *petG*, *petL*, *petN*
*ATP synthase*	*atpA*, *atpB*, *atpE*, *atpF*, *atpH*, *atpI*
*NADH-dehydrogenase*	*ndhC*, *ndhF*, *ndhJ*, *ndhK*
*Large subunit Rubisco*	*rbcL*
*Acetyl-CoA carboxylase*	*accD*
*Maturase*	*matk*
*Inner membrane protein*	*cemA*
*Conserved open reading frames*	*ycf3*, *ycf4*

## Data Availability

The data presented in this study are available upon request from NCBI (https://www.ncbi.nlm.nih.gov/, accessed on 1 March 2021) and National Genomics Data Center (https://ngdc.cncb.ac.cn/, accessed on 1 November 2021), accession number GWHBGBH00000000.

## Supplementary Materials

The following supporting information can be downloaded at: https://www.mdpi.com/2223-7747/11/1/52/s1, Figure S1: The circular chloroplast genome map of 10 assembled chloroplast genome; Figure S2: 50 phylogenetic results constructed by unique chloroplast genes; Figure S3: Phylogeny analysis of the entire chloroplast genome of 26 species; Figure S4: The alignment of chloroplast genomes of *A. sinensis*, *C. cathayensis* and *C. kweichowensis*; Table S1: Assembled chloroplast genome of 10 species; Table S2: Complete chloroplast genome of 15 species from NCBI; Table S3: NCBI accession number of assembled chloroplast genomes; Table S4: Mutual coding genes within all 26 chloroplast genomes (73 in total).

## References

[1] Long X. (2002). Annamocarya Sinensis, a Rare Species of Guizhou, China. For. By-Prod. Spec. China.

[2] Zhang Z.Y., Pang X.M., Han J.W., Wang Y., Li Y.Y. (2013). Conservation Genetics of Annamocarya Sinensis (Dode) Leroy, an Endangered Endemic Species. Genet. Mol. Res..

[3] Grauke L.J., Wood B., Payne J. (1991). Genetic Resources of Carya in Vietnam and China. Ann. Report Northern Nut Growers Assoc..

[4] Manning W.E., Hjelmqvist H. (1951). Annamocarya, Rhamphocarya, and Carya Sinensis. Botaniska Notiser..

[5] Chevalier A. (1941). Variabilité Et Hybridité Chez Les Noyers. Notes Sur Des Juglans Peu Connus, Sur L’annamocarya Et Un Carya D’indochine. Rev. Bot. Appl. d’Agric. Coloniale.

[6] Manos P.S., Stone D.E. (2001). Evolution, Phylogeny, and Systematics of the Juglandaceae. Ann. Mo. Bot. Gard..

[7] Leroy J.F. (1955). Etude sur les Juglandaceae: A la Recherche d’une Concepcion Morphologique de la Fleur Femelle et du Fruit.

[8] Li R., Chen Z., Lu A., Soltis D.E., Soltis P.S., Manoss P.S. (2004). Phylogenetic Relationships in Fagales Based on DNA Sequences from Three Genomes. Int. J. Plant Sci..

[9] Manos P.S., Soltis P.S., Soltis D.E., Manchester S.R., Oh S.-H., Bell C.D., Dilcher D.L., Stone D.E. (2007). Phylogeny of Extant and Fossil Juglandaceae Inferred from the Integration of Molecular and Morphological Data Sets. Syst. Biol..

[10] Zhang J.-B., Li R.-Q., Xiang X.-G., Manchester S.R., Lin L., Wang W., Wen J., Chen Z.-D. (2013). Integrated Fossil and Molecular Data Reveal the Biogeographic Diversification of the Eastern Asian-Eastern North American Disjunct Hickory Genus (Carya Nutt.). PLoS ONE.

[11] Leebens-Mack J., Raubeson L.A., Cui L., Kuehl J.V., Fourcade M.H., Chumley T.W., Boore J.L., Jansen R.K., Claude W., DePamphilis C.W. (2005). Identifying the Basal Angiosperm Node in Chloroplast Genome Phylogenies: Sampling One’s Way out of the Felsenstein Zone. Mol. Biol. Evol..

[12] Alzahrani D.A., Yaradua S.S., Albokhari E.J., Abba A. (2020). Complete chloroplast genome sequence of Barleria prionitis, comparative chloroplast genomics and phylogenetic relationships among Acanthoideae. BMC Genom..

[13] Wu Z., Gui S., Quan Z., Pan L., Wang S., Ke W., Liang D., Ding Y. (2014). A precise chloroplast genome of Nelumbo nucifera (Nelumbonaceae) evaluated with Sanger, Illumina MiSeq, and PacBio RS II sequencing platforms: Insight into the plastid evolution of basal eudicots. BMC Plant Biol..

[14] Wu Z., Liao R., Yang T., Dong X., Lan D., Qin R., Liu H. (2020). Analysis of six chloroplast genomes provides insight into the evolution of Chrysosplenium (Saxifragaceae). BMC Genom..

[15] Rabiee M., Sayyari E., Mirarab S. (2019). Multi-Allele Species Reconstruction Using Astral. Mol. Phylogenet. Evol..

[16] Roch S., Steel M. (2015). Likelihood-based tree reconstruction on a concatenation of aligned sequence data sets can be statistically inconsistent. Theor. Popul. Biol..

[17] Sayyari E., Mirarab S. (2016). Fast Coalescent-Based Computation of Local Branch Support from Quartet Frequencies. Mol. Biol. Evol..

[18] Xi Z., Liu L., Rest J.S., Davis C.C. (2014). Coalescent Versus Concatenation Methods and the Placement of Amborella as Sister to Water Lilies. Syst. Biol..

[19] Kubatko L.S., Degnan J.H. (2007). Inconsistency of Phylogenetic Estimates from Concatenated Data under Coalescence. Syst. Biol..

[20] Simmons M.P., Gatesy J. (2015). Coalescence Vs. Concatenation: Sophisticated Analyses Vs. First Principles Applied to Rooting the Angiosperms. Mol. Phylogenet. Evol..

[21] Edwards S.V., Xi Z., Janke A., Faircloth B., McCormack J.E., Glenn T.C., Zhong B., Wu S., Lemmon E.M., Lemmon A.R. (2016). Implementing and testing the multispecies coalescent model: A valuable paradigm for phylogenomics. Mol. Phylogenet. Evol..

[22] Gonçalves D.J., Simpson B.B., Ortiz E.M., Shimizu G.H., Jansen R.K. (2019). Incongruence between gene trees and species trees and phylogenetic signal variation in plastid genes. Mol. Phylogenet. Evol..

[23] Xi Z., Rest J.S., Davis C.C. (2013). Phylogenomics and Coalescent Analyses Resolve Extant Seed Plant Relationships. PLoS ONE.

[24] Tillich M., Lehwark P., Pellizzer T., Ulbricht-Jones E.S., Fischer A., Bock R., Greiner S. (2017). GeSeq—Versatile and accurate annotation of organelle genomes. Nucleic Acids Res..

[25] Ye L., Fu C., Wang Y., Liu J., Gao L.-M. (2018). Characterization of the complete plastid genome of a Chinese endemic species Carya kweichowensis. Mitochondrial DNA Part B.

[26] Favre F., Jourda C., Besse P., Charron C. (2020). Genotyping-by-Sequencing Technology in Plant Taxonomy and Phylogeny. Molecular Plant Taxonomy.

[27] Huang Y., Xiao L., Zhang Z., Zhang R., Wang Z., Huang C., Huang R., Luan Y., Fan T., Wang J. (2019). The genomes of pecan and Chinese hickory provide insights into Carya evolution and nut nutrition. GigaScience.

[28] Wang X., Rhein H.S., Jenkins J., Schmutz J., Grimwood J., Grauke L.J., Randall J.J. (2020). Chloroplast Genome Sequences of Carya Illinoinensis from Two Distinct Geographic Populations. Tree Genet. Genomes.

[29] Mu X.-Y., Tong L., Sun M., Zhu Y.-X., Wen J., Lin Q.-W., Liu B. (2020). Phylogeny and divergence time estimation of the walnut family (Juglandaceae) based on nuclear RAD-Seq and chloroplast genome data. Mol. Phylogenet. Evol..

[30] Salas-Leiva D.E., Meerow A.W., Calonje M., Griffith M.P., Francisco-Ortega J., Nakamura K., Stevenson D.W., Lewis C.E., Namoff S. (2013). Phylogeny of the Cycads Based on Multiple Single-Copy Nuclear Genes: Congruence of Concatenated Parsimony, Likelihood and Species Tree Inference Methods. Ann. Bot..

[31] CNCB-NGDC Members and Partners (2021). Database Resources of the National Genomics Data Center, China National Center for Bioinformation in 2022. Nucleic Acids Res..

[32] Chen M., Ma Y., Wu S., Zheng X., Kang H., Sang J., Xu X., Hao L., Li Z., Gong Z. (2021). Genome Warehouse: A Public Repository Housing Genome-scale Data. Genom. Proteom. Bioinform..

[33] Chen S., Zhou Y., Chen Y., Gu J. (2018). fastp: An ultra-fast all-in-one FASTQ preprocessor. Bioinformatics.

[34] Li H. (2012). Exploring single-sample SNP and INDEL calling with whole-genome de novo assembly. Bioinformatics.

[35] Li H., Handsaker B., Wysoker A., Fennell T., Ruan J., Homer N., Marth G., Abecasis G., Durbin R., 1000 Genome Project Data Processing Subgroup (2009). The Sequence Alignment/Map format and SAMtools. Bioinformatics.

[36] Li H. (2011). A statistical framework for SNP calling, mutation discovery, association mapping and population genetical parameter estimation from sequencing data. Bioinformatics.

[37] Jin J.-J., Yu W.-B., Yang J.-B., Song Y., Depamphilis C.W., Yi T.-S., Li D.-Z. (2020). GetOrganelle: A fast and versatile toolkit for accurate de novo assembly of organelle genomes. Genome Biol..

[38] Emms D.M., Kelly S. (2019). OrthoFinder: Phylogenetic orthology inference for comparative genomics. Genome Biol..

[39] Emms D.M., Kelly S. (2015). Orthofinder: Solving Fundamental Biases in Whole Genome Comparisons Dramatically Improves Orthogroup Inference Accuracy. Genome Biol..

[40] Katoh K., Asimenos G., Toh H. (2009). Multiple Alignment of DNA Sequences with Mafft. Methods Mol. Biol..

[41] Katoh K., Standley D.M. (2013). Mafft Multiple Sequence Alignment Software Version 7: Improvements in Performance and Usability. Mol. Biol. Evol..

[42] Minh B.Q., Schmidt H.A., Chernomor O., Schrempf D., Woodhams M.D., von Haeseler A., Lanfear R. (2020). Iq-Tree 2: New Models and Efficient Methods for Phylogenetic Inference in the Genomic Era. Mol. Biol. Evol..

[43] Price M.N., Dehal P.S., Arkin A.P. (2009). FastTree: Computing Large Minimum Evolution Trees with Profiles instead of a Distance Matrix. Mol. Biol. Evol..

[44] Price M.N., Dehal P.S., Arkin A.P. (2010). Fasttree 2—Approximately Maximum-Likelihood Trees for Large Alignments. PLoS ONE.

[45] Letunic I., Bork P. (2021). Interactive Tree Of Life (iTOL) v5: An online tool for phylogenetic tree display and annotation. Nucleic Acids Res..

[46] Shen W., Le S., Li Y., Hu F. (2016). SeqKit: A Cross-Platform and Ultrafast Toolkit for FASTA/Q File Manipulation. PLoS ONE.

[47] Smith S.A., Moore M.J., Brown J.W., Yang Y. (2015). Analysis of phylogenomic datasets reveals conflict, concordance, and gene duplications with examples from animals and plants. BMC Evol. Biol..

