# Correction: Luo et al. Reassessment of *Annamocarya sinesis* (*Carya sinensis*) Taxonomy through Concatenation and Coalescence Phylogenetic Analysis. *Plants* 2022, *11*, 52

**DOI:** 10.3390/plants11233282

**Published:** 2022-11-29

**Authors:** Jie Luo, Junhao Chen, Wenlei Guo, Zhengfu Yang, Kean-Jin Lim, Zhengjia Wang

**Affiliations:** 1 State Key Laboratory of Subtropical Silviculture, College of Forestry and Biotechnology, Zhejiang A&F University, Lin’an, Hangzhou 311300, China; 2 Department of Biology, Saint Louis University, St. Louis, MO 63104, USA; 3 State Key Laboratory of Integrated Management of Pest Insects and Rodents, Institute of Zoology, Chinese Academy of Sciences, Beijing 100101, China

In the original publication [1], there was a mistake in Table 2 as published. 

Minor errors were found in the number of tRNA and rRNA genes, CDS length and GC content in Table 2. We recalculated and corrected the statistics and fixed the numbers in Table 2.

The corrected Table 2 appear below. The authors state that the scientific conclusions are unaffected. This correction was approved by the Academic Editor. The original publication has also been updated.

## Figures and Tables

**Table 2:** Chloroplast genome features of 26 species.

Species	Genome Size (bp)	Coding Gene Number	tRNA Genes	rRNA Genes	CDS Total Length (bp)	CDS GC Content (%)
*A. sinensis*	158,484	79	35	8	68,261	37.26
*B. platyphylla*	160,518	84	37	8	78,972	37.43
*C. aquatica*	160,763	79	37	8	68,673	37.24
*C. cathayensis*	160,666	80	36	8	69,595	37.22
*C. cordiformis*	160,796	79	37	8	68,673	37.24
*C. dabieshanensis*	160,037	80	37	8	69,250	37.24
*C. glabra*	160,652	79	37	8	68,674	37.25
*C. hunanensis*	160,397	80	36	8	69,449	37.21
*C. illinoinensis*	160,819	79	37	8	68,673	37.25
*C. kweichowensis*	175,313	79	38	8	68,314	37.27
*C. laciniosa*	160,771	79	37	8	68,672	37.26
*C. myristiciformis*	160,788	79	37	8	68,673	37.24
*C. ovata*	160,727	79	37	8	68,680	37.26
*C. palmeri*	160,604	79	37	8	68,673	37.25
*C. texana*	160,745	79	37	8	68,674	37.25
*C. tomentosa*	160,784	79	37	8	68,673	37.25
*Cy. paliurus*	160,562	89	40	8	81,015	37.19
*J. cathayensis*	159,730	87	40	8	80,331	37.26
*J. cinerea*	160,288	84	37	8	78,333	37.26
*J. hopeiensis*	159,714	86	40	8	80,259	37.27
*J. major*	160,276	83	37	8	77,970	37.2
*J. nigra*	160,274	83	37	8	77,958	37.21
*J. regia*	160,370	83	37	8	77,979	37.2
*P. strobilacea*	160,994	85	36	8	78,915	37.18
*Pt. hupehensis*	159,770	89	40	8	81,315	37.2
*Pt. stenoptera*	160,202	89	40	8	81,021	37.23
