# Supplementary material for: Reassessment of Annamocarya sinesis (Carya sinensis) Taxonomy through Concatenation and Coalescence Phylogenetic Analysis
Source: Plants (Basel). 2021 Dec 24;11(1):52. doi: 10.3390/plants11010052 (PMC8747223; doi:10.3390/plants11010052)
Supplement: Supplementary file 1 [file plants-11-00052-s001.zip › supplementary_materials_correction/Supplementary_Tables.pdf]

Supplementary Table S1: Assembled chloroplast genome of 10 species

Assemble Species	SRA (From NCBI)	Reference species	Reference chloroplast genome of NCBI
Carya aquatica	SRR6804841	*C. illinoinensis*	MH909600
Carya cordiformis	SRR6804840	*C. illinoinensis*	MH909600
Carya dabieshanensis	SRR6804849	*C. cathayensis*	NC_046572
Carya glabra	SRR6804848	*C. illinoinensis*	MH909600
Carya laciniosa	SRR6804855	*C. illinoinensis*	MH909600
Carya myristiciformis	SRR6804845	*C. illinoinensis*	MH909600
Carya ovata	SRR6804846	*C. illinoinensis*	MH909600
Carya palmeri	SRR6804847	*C. illinoinensis*	MH909600
Carya texana	SRR6804850	*C. illinoinensis*	MH909600
Carya tomentosa	SRR6804842	*C. illinoinensis*	MH909600

Supplementary Table S2: Complete chloroplast genome of 15 species from NCBI

Species	NCBI accession	Species	NCBI accession
Annamocarya sinensis	MN911165.1	*Juglans hopeiensis*	NC_033894.1
Betula platyphylla	MH205735.1	*Juglans major*	NC_035966.1
Carya cathayensis	NC_046572.1	*Juglans nigra*	NC_035967.1
Carya hunanensis	NC_046435.1	*Juglans regia*	MF167463.1
Carya kweichowensis	NC_040864.1	*Pterocarya hupehensis*	NC_046431.1
Cyclocarya paliurus	NC_034315.1	*Pterocarya stenoptera*	NC_046428.1
Juglans cathayensis	NC_033893.1	*Platycarya strobilacea*	NC_035413.1
Juglans cinerea	NC_035960.1		

Supplementary Table S3: NCBI accession number of assembled chloroplast genome

Species	Accession	Species	Accession
Carya aquatica	BK061153	*Carya myristiciformis*	BK061158
Carya cordiformis	BK061154	*Carya ovata*	BK061159
Carya dabieshanensis	BK061155	*Carya palmeri*	BK061160
Carya glabra	BK061156	*Carya texana*	BK061161
Carya laciniosa	BK061157	*Carya tomentosa*	BK061162

Supplementary Table S4: Mutual coding genes within all 26 chloroplast genomes (73 in total)

Gene ID					
accD	*ndhD*	*petN*	*psbJ*	*rpl33*	*rps3*
atpA	*ndhE*	*psaA*	*psbK*	*rpl36*	*rps4*
atpB	*ndhF*	*psaB*	*psbL*	*rpoA*	*rps7*
atpE	*ndhG*	*psaC*	*psbM*	*rpoB*	*rps8*
atpF	*ndhH*	*psaI*	*psbT*	*rpoC1*	*ycf1*
atpH	*ndhI*	*psaJ*	*rbcL*	*rpoC2*	*ycf2*
atpI	*ndhJ*	*psbA*	*rpl14*	*rps11*	*ycf3*
ccsA	*ndhK*	*psbC*	*rpl16*	*rps12*	*ycf4*
cemA	*petA*	*psbD*	*rpl2*	*rps14*	
matK	*petB*	*psbE*	*rpl20*	*rps15*	
ndhA	*petD*	*psbF*	*rpl22*	*rps18*	
ndhB	*petG*	*psbH*	*rpl23*	*rps19*	
ndhC	*petL*	*psbI*	*rpl32*	*rps2*	
