# Supplementary material for: Reassessment of Annamocarya sinesis (Carya sinensis) Taxonomy through Concatenation and Coalescence Phylogenetic Analysis
Source: Plants (Basel). 2021 Dec 24;11(1):52. doi: 10.3390/plants11010052 (PMC8747223; doi:10.3390/plants11010052)
Supplement: Supplementary file 1 [file plants-11-00052-s001.zip › supplementary_materials_correction/Supplementary_Figure_3.pdf]

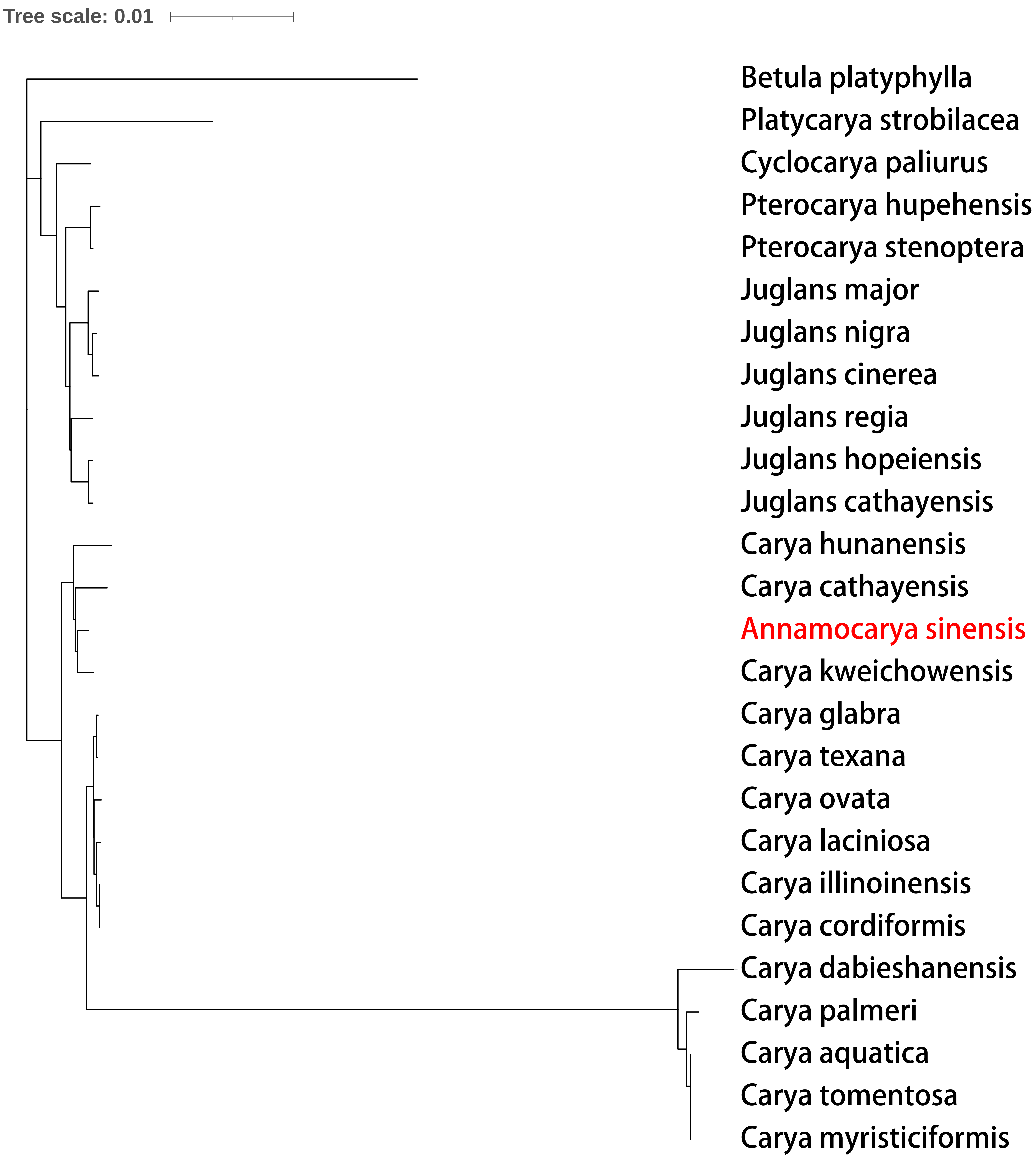

Figure S3: Phylogeny analysis of the entire chloroplast genome of 26 species. All species were classified into expected genus. However, *Carya dabieshanensis*, which should belong to the East Asian branch (based on geographical distribution and our results), was clustered into the North American branch. The phylogenetic analysis was performed using Fasttree, and ITOL was used for visualization.
