# Supplementary material for: Reassessment of Annamocarya sinesis (Carya sinensis) Taxonomy through Concatenation and Coalescence Phylogenetic Analysis
Source: Plants (Basel). 2021 Dec 24;11(1):52. doi: 10.3390/plants11010052 (PMC8747223; doi:10.3390/plants11010052)
Supplement: Supplementary file 1 [file plants-11-00052-s001.zip › supplementary_materials_correction/Supplementary_Figure_2.pdf]

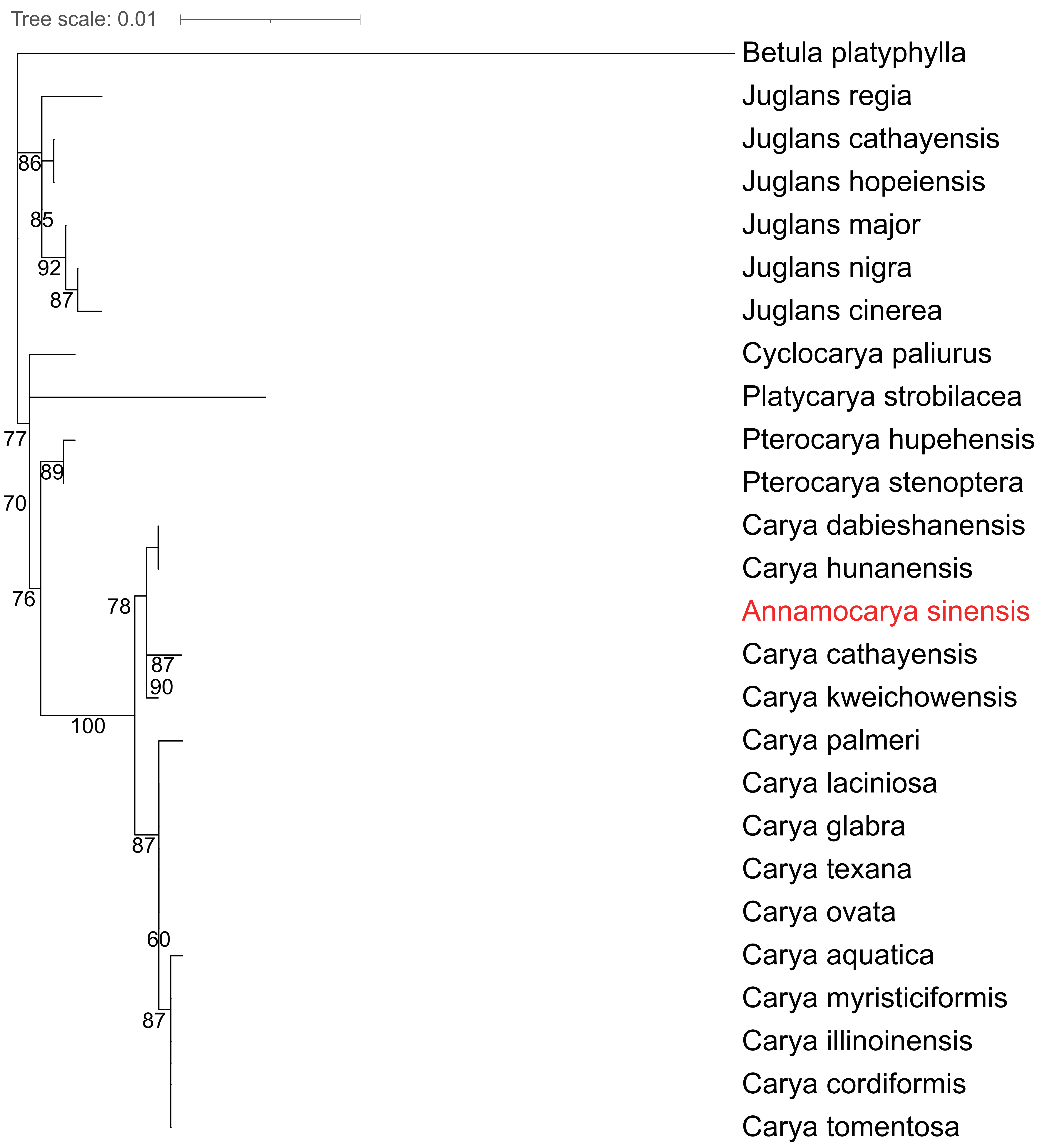

Figure S2A1. Phylogenetic tree constructed using accD

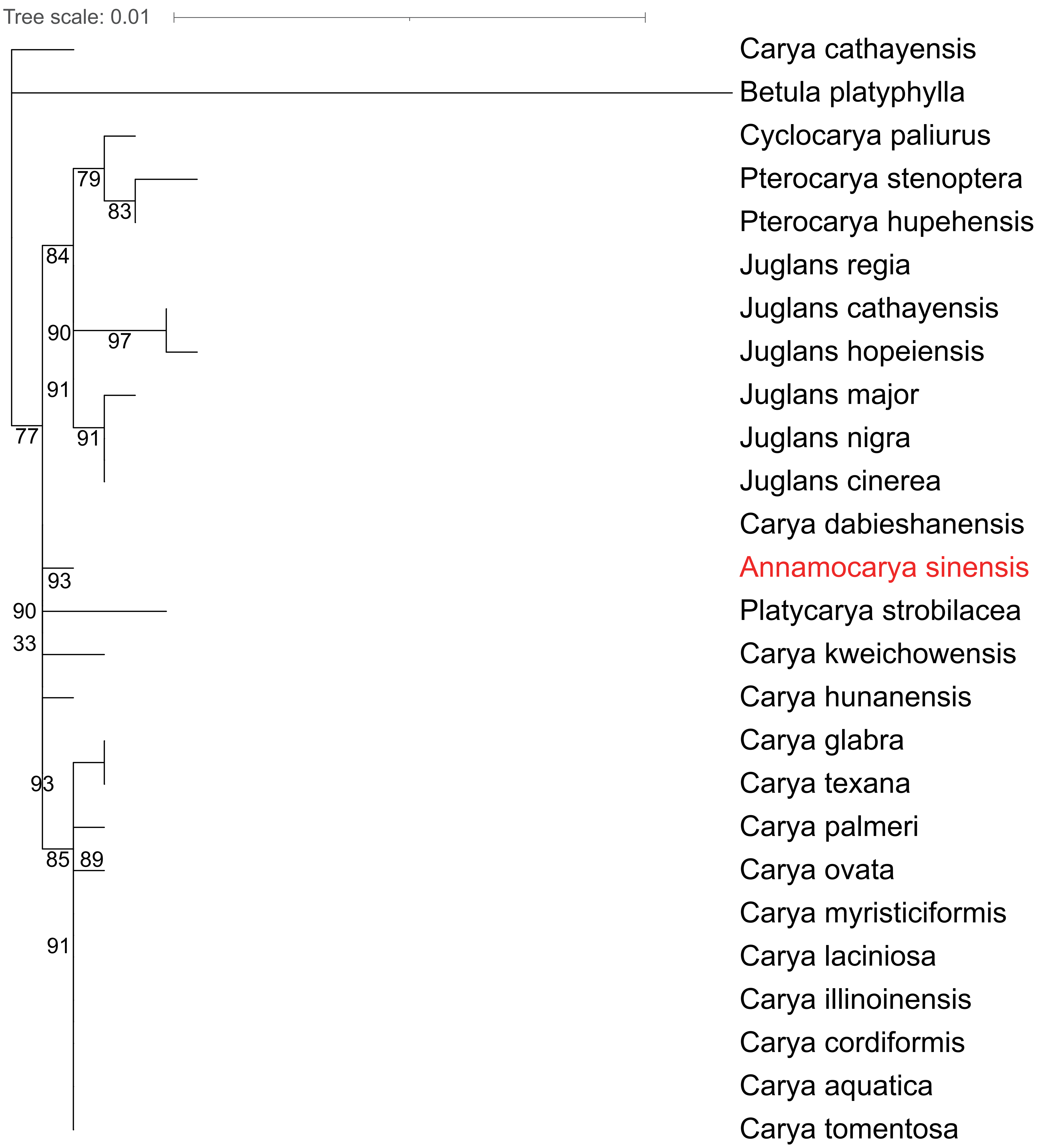

Figure S2A2. Phylogenetic tree constructed using atpA

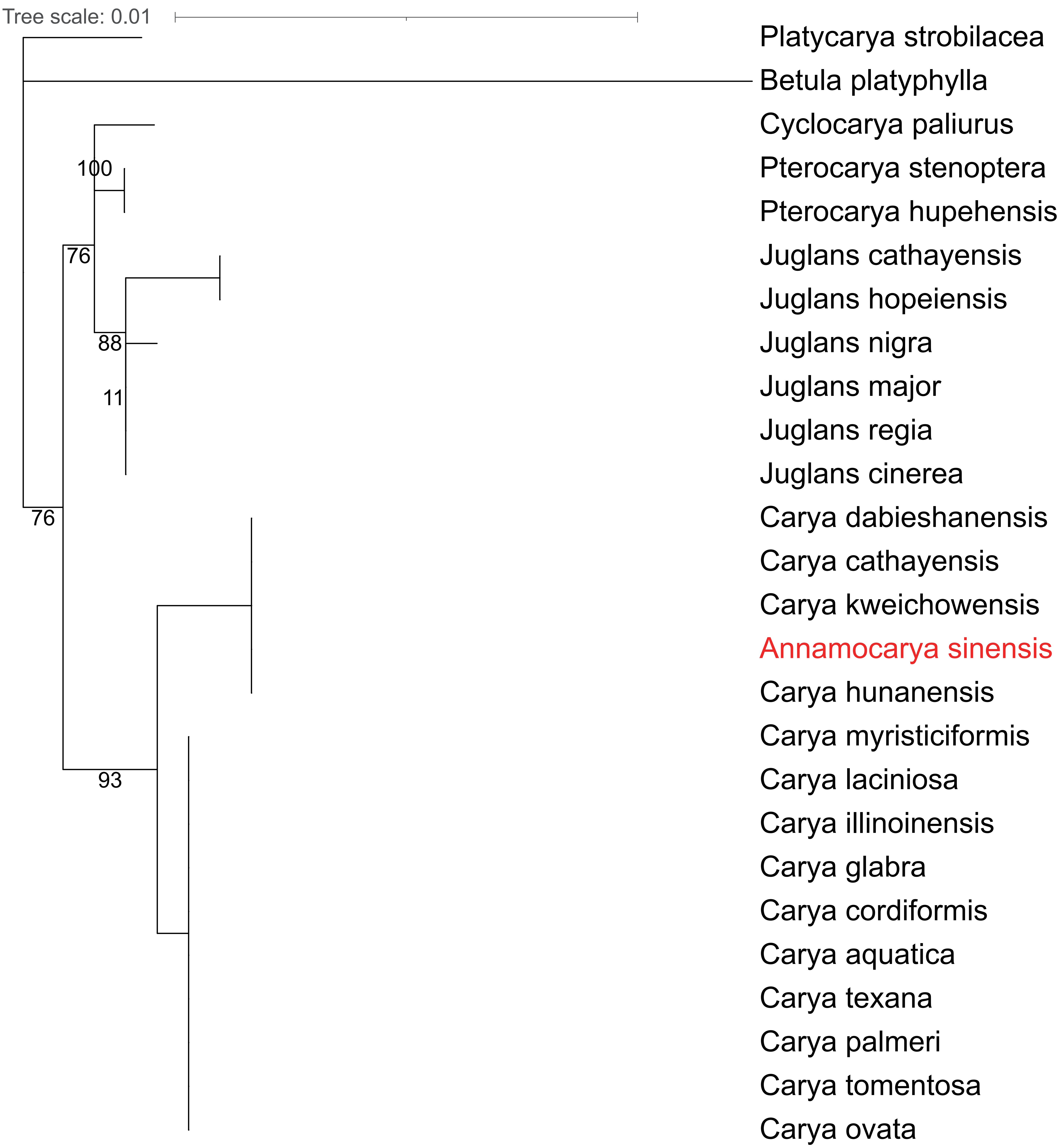

Figure S2A3. Phylogenetic tree constructed using atpB

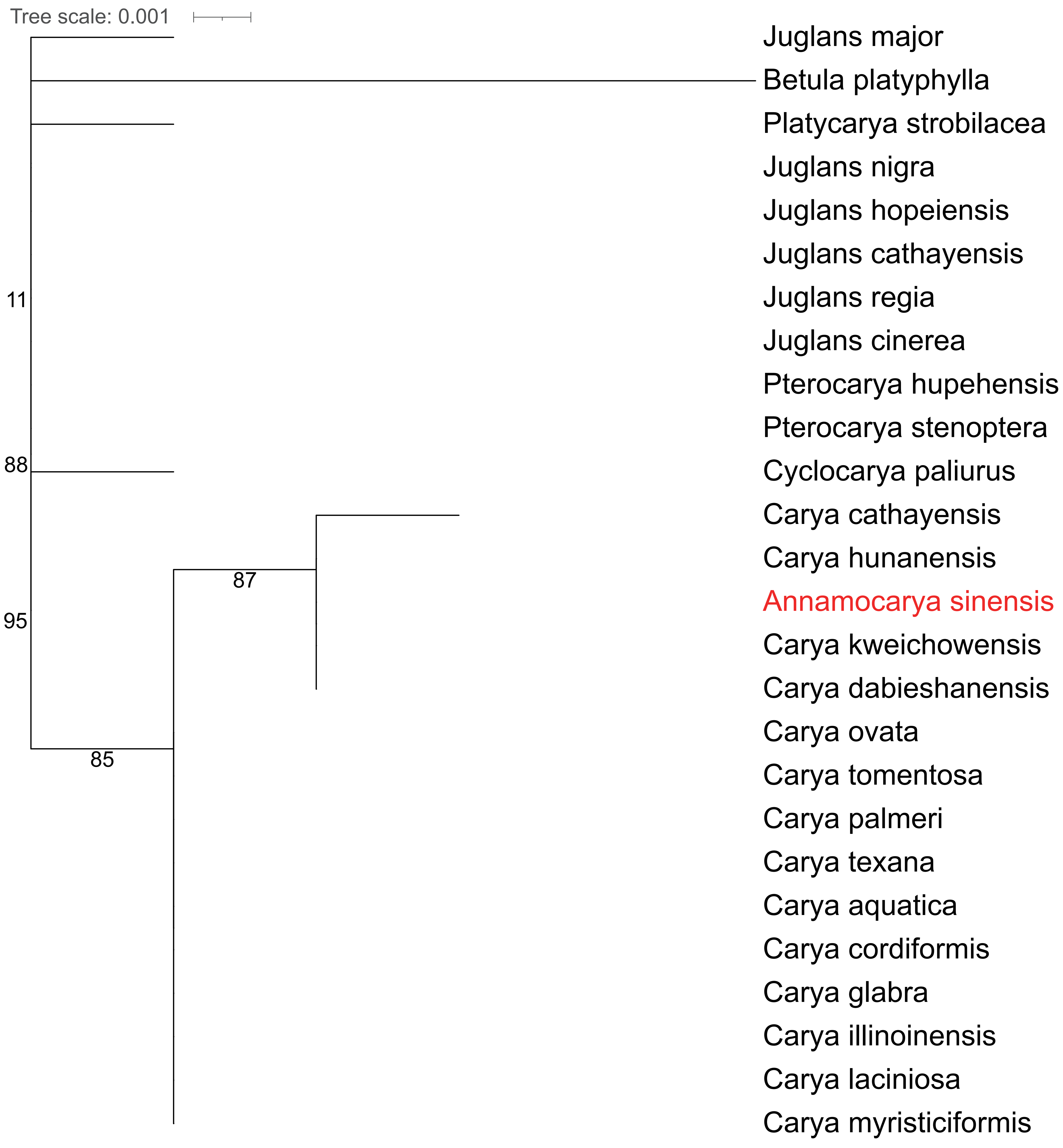

Figure S2A4. Phylogenetic tree constructed using atpE

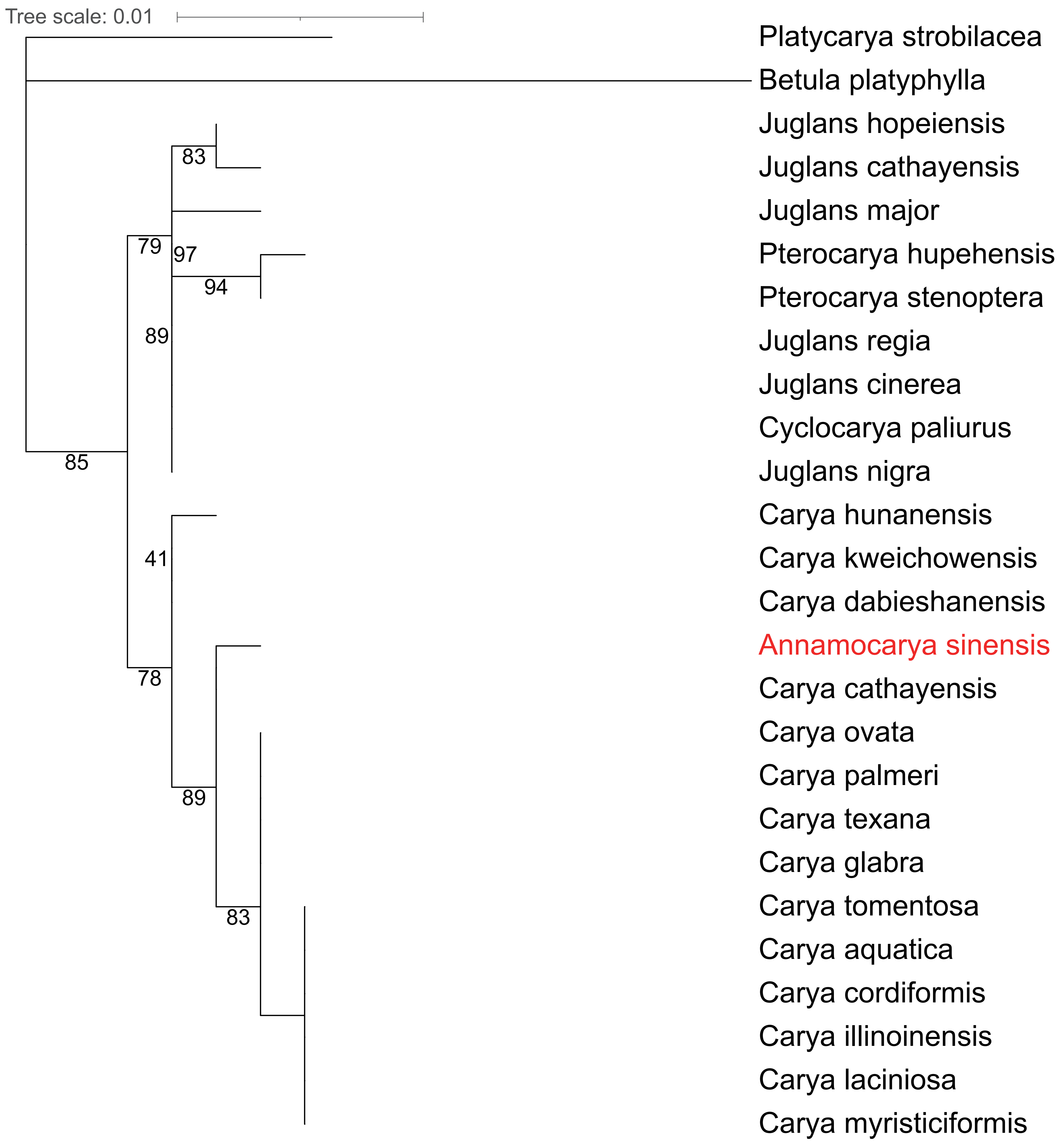

Figure S2A5. Phylogenetic tree constructed using atpF

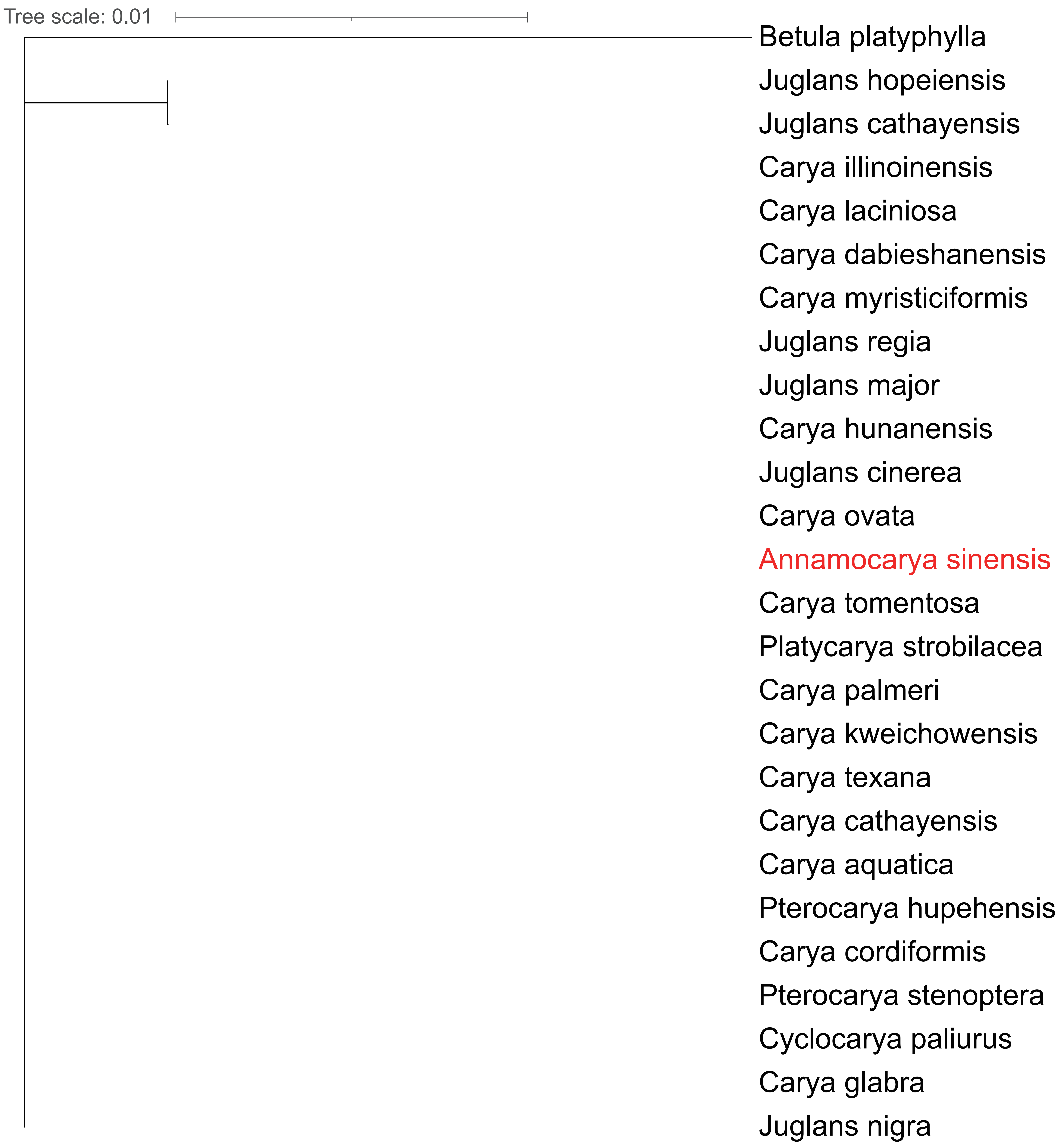

Figure S2A6. Phylogenetic tree constructed using atpH

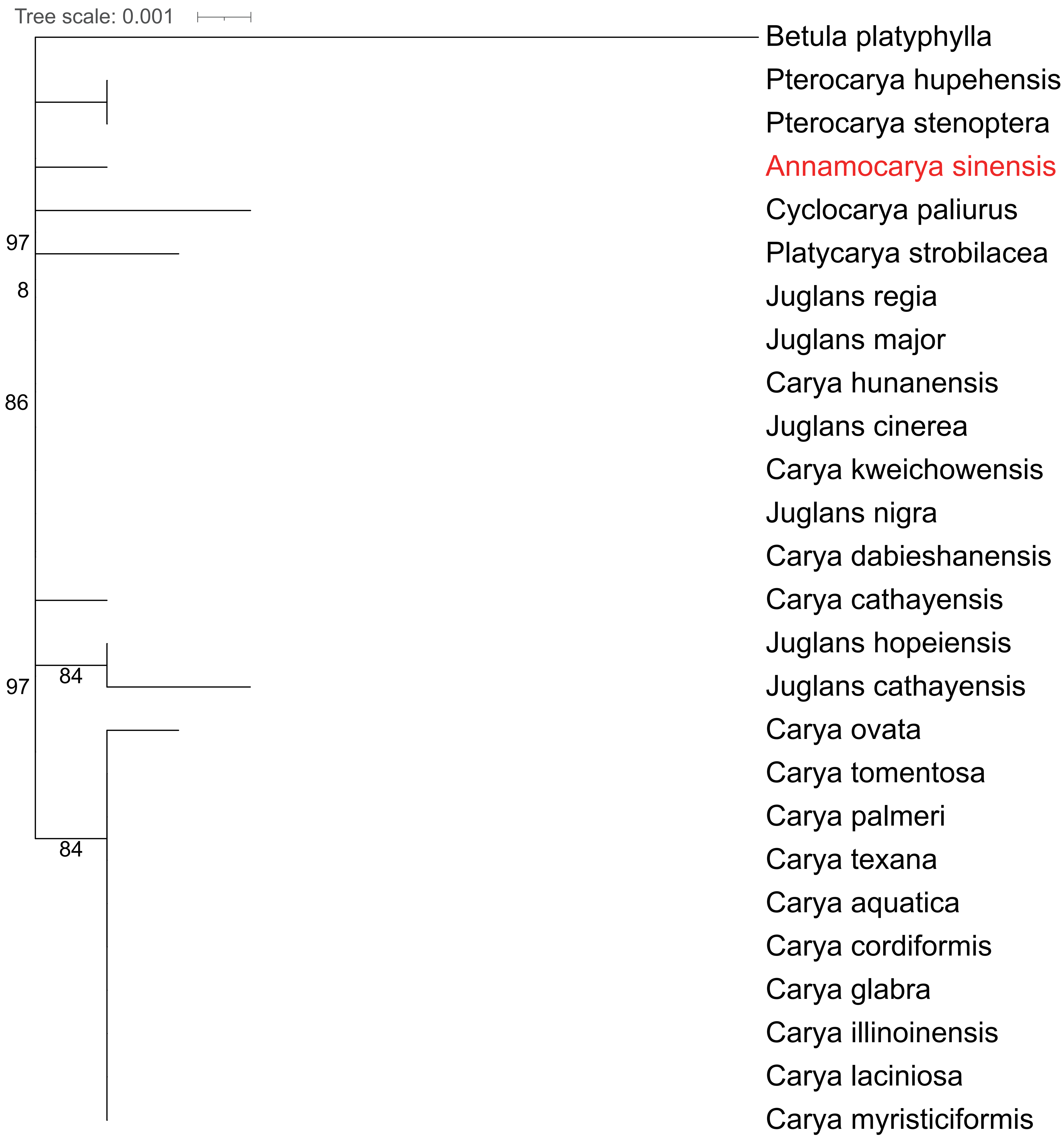

Figure S2A7. Phylogenetic tree constructed using atpI

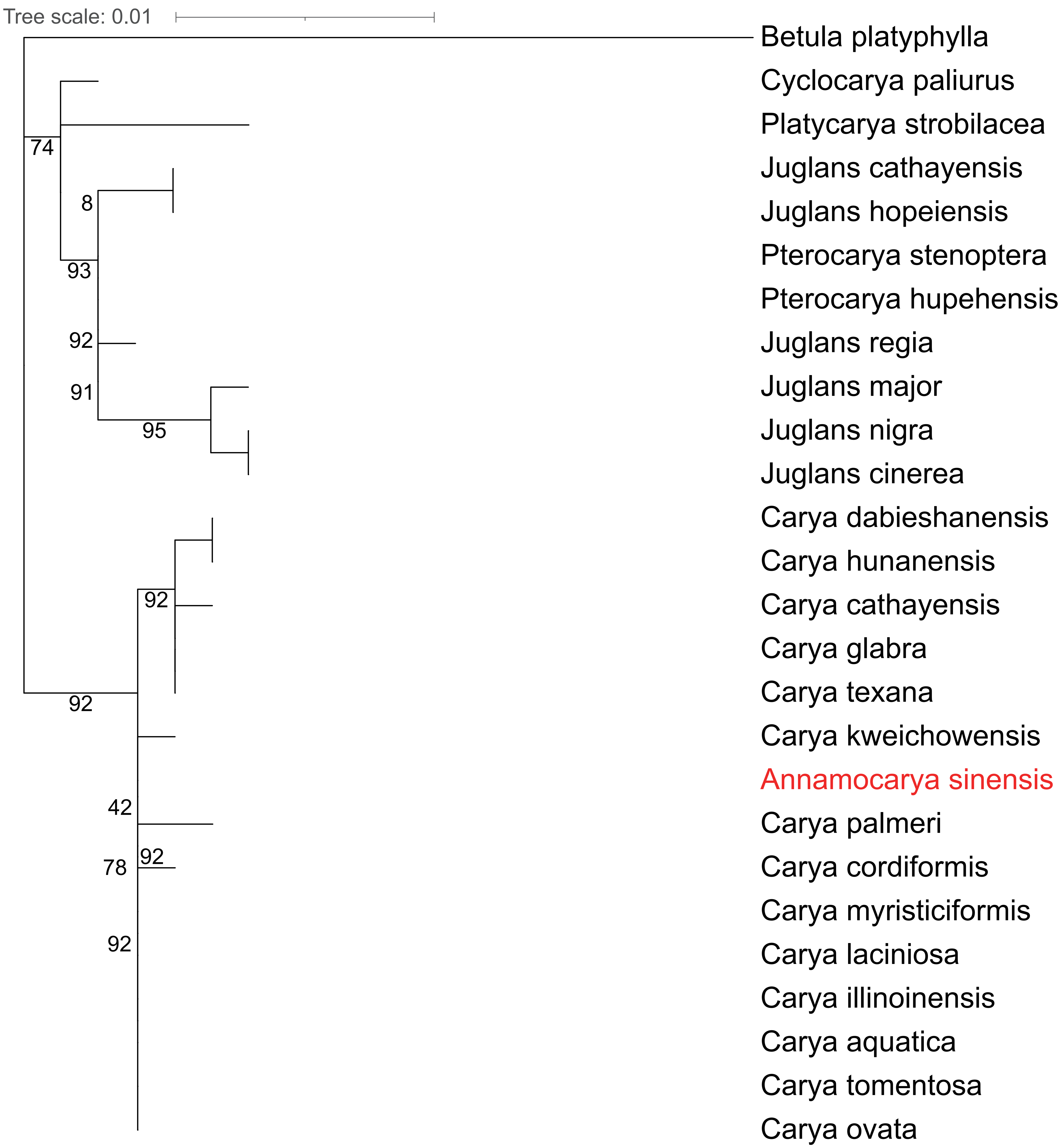

Figure S2A8. Phylogenetic tree constructed using cemA

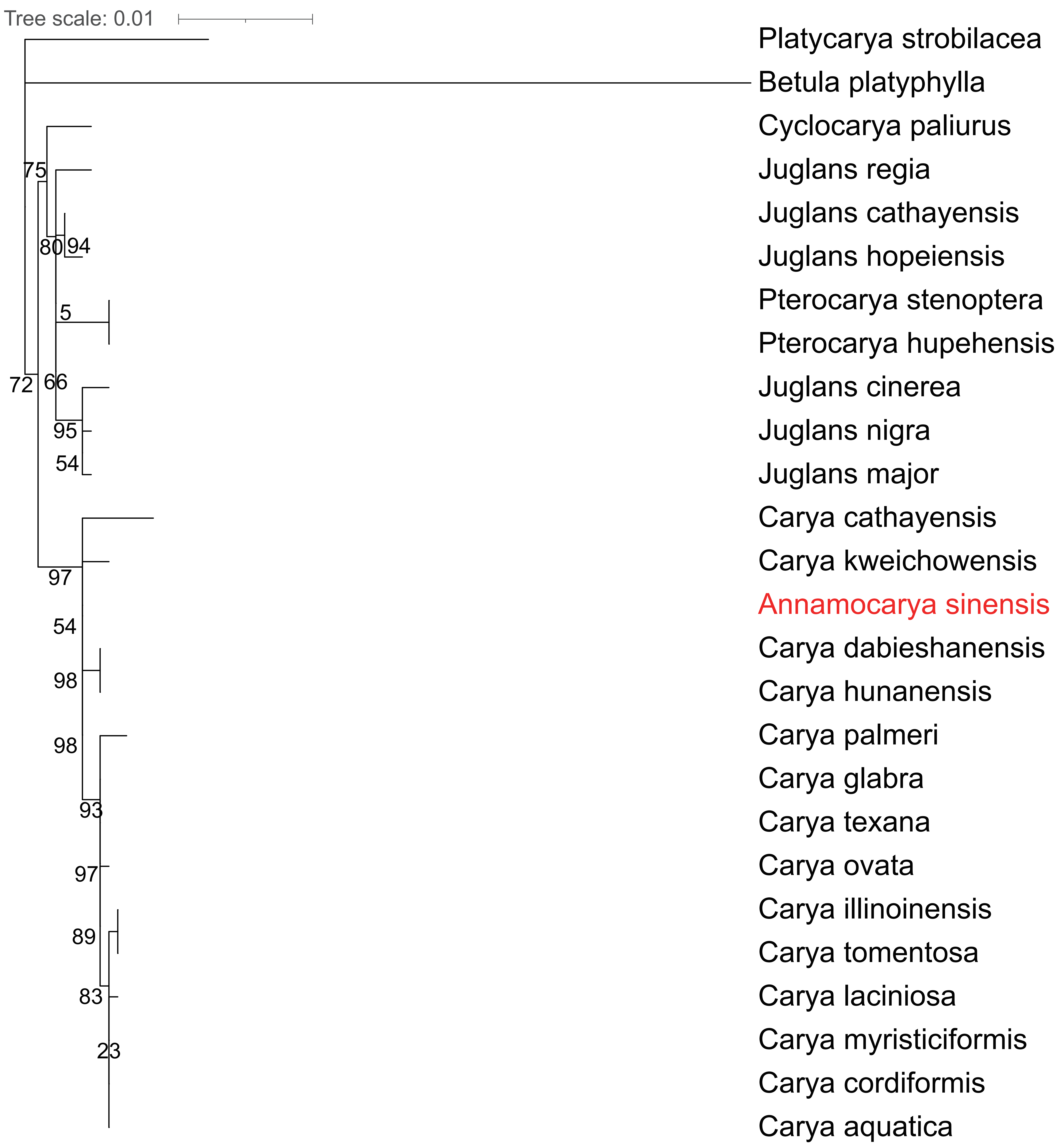

Figure S2A9. Phylogenetic tree constructed using matK

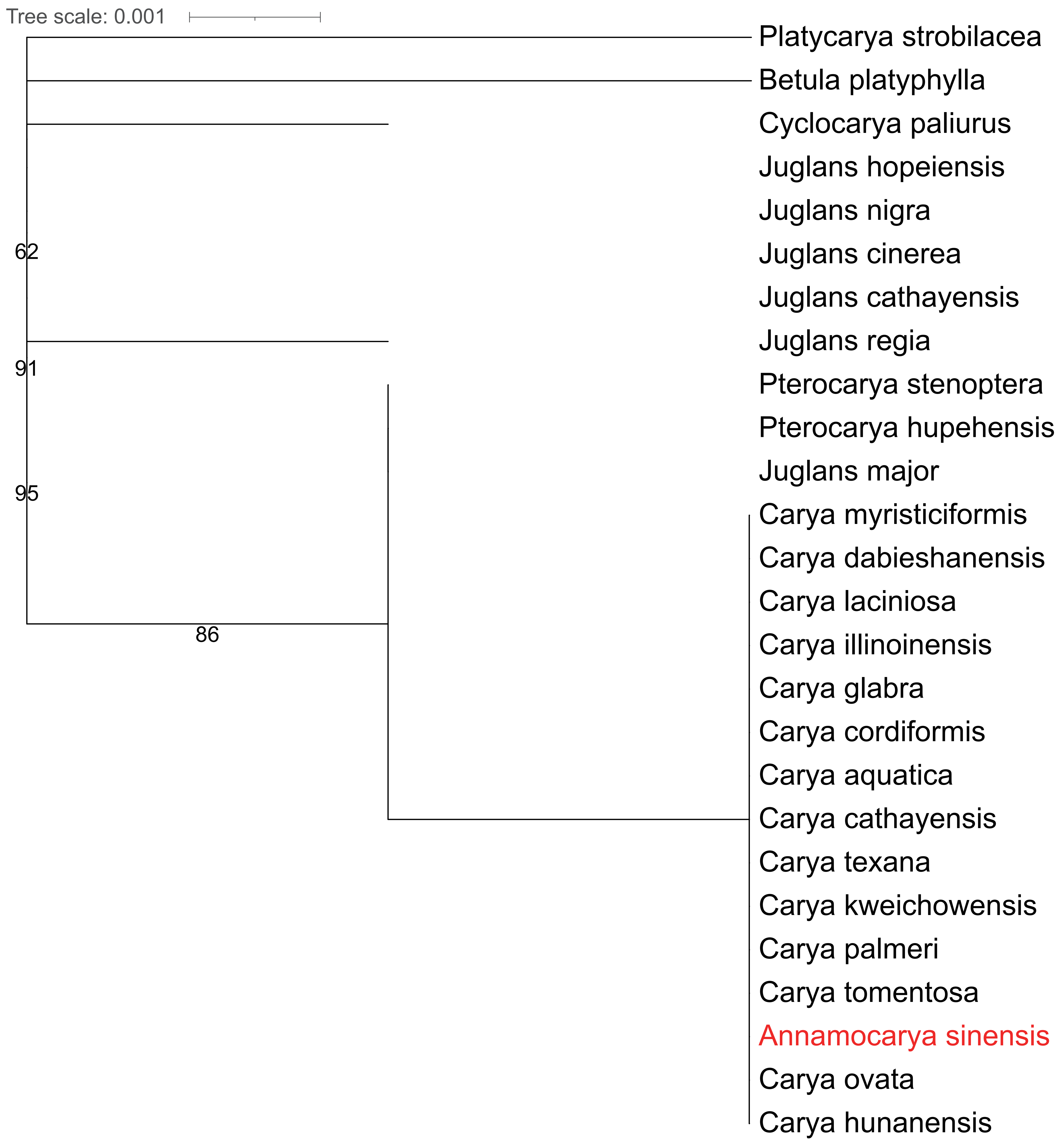

Figure S2A10. Phylogenetic tree constructed using ndhC

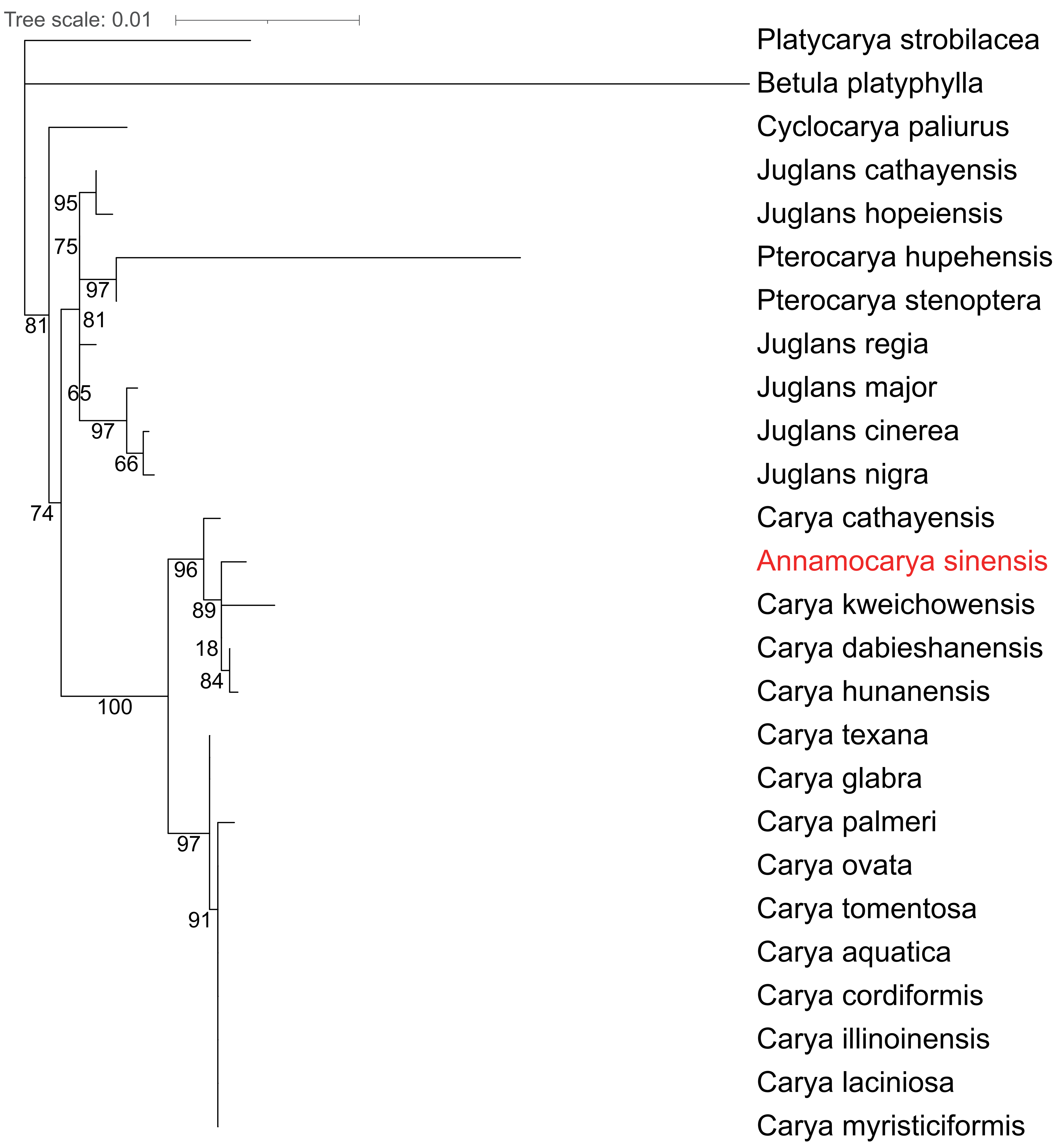

Figure S2A11. Phylogenetic tree constructed using ndhF

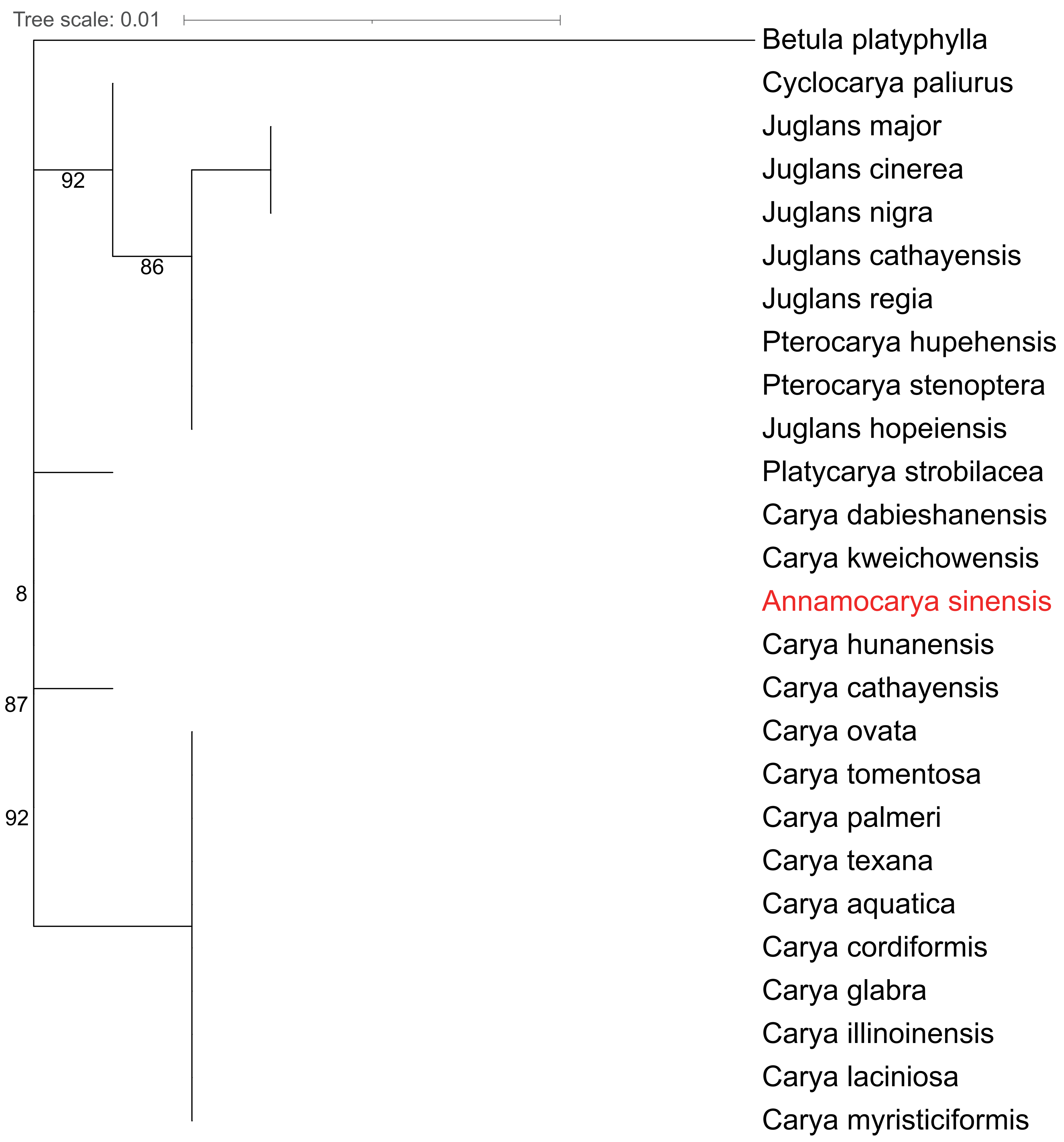

Figure S2A12. Phylogenetic tree constructed using ndhJ

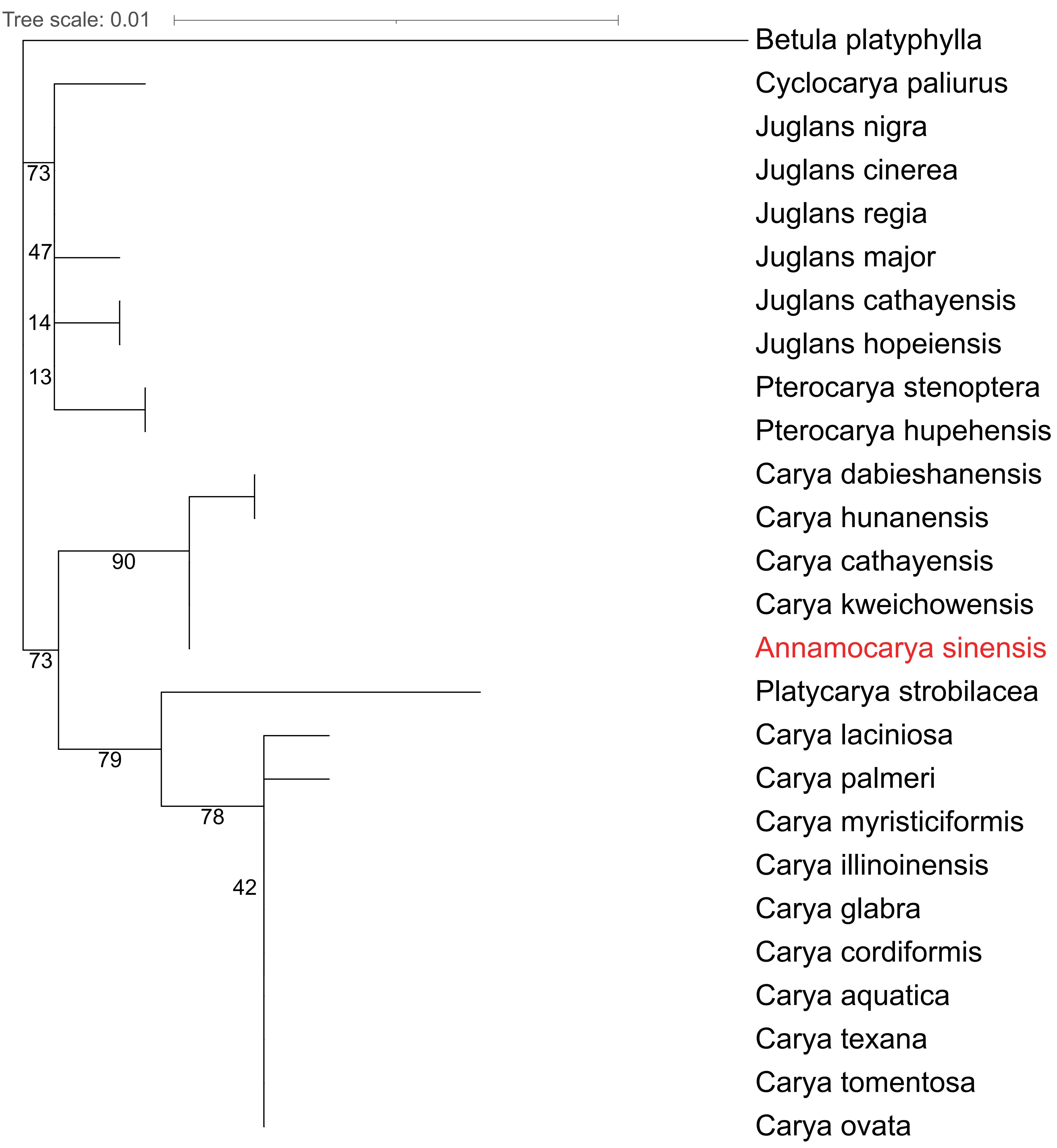

Figure S2A13. Phylogenetic tree constructed using ndhK

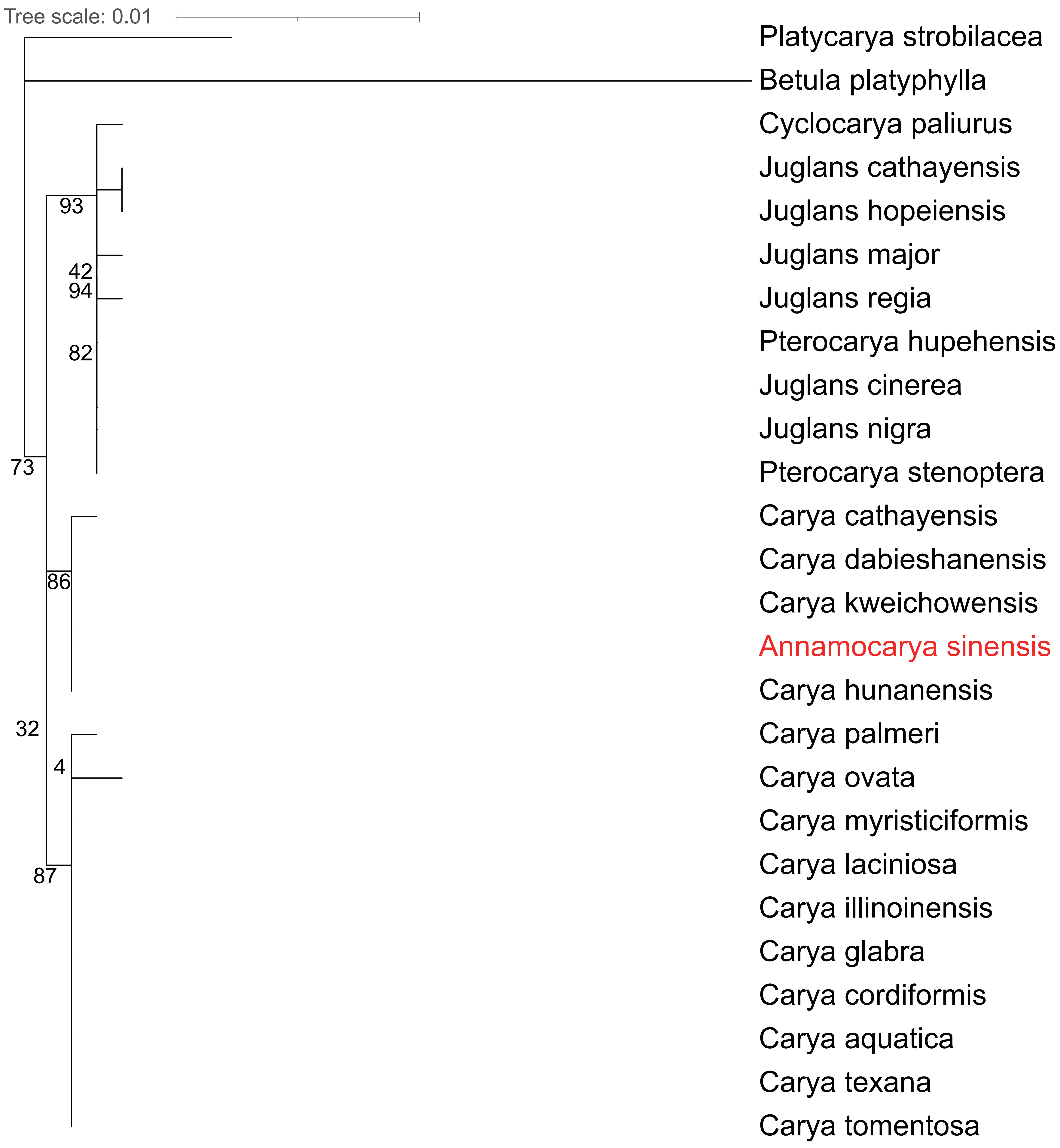

Figure S2A14. Phylogenetic tree constructed using petA

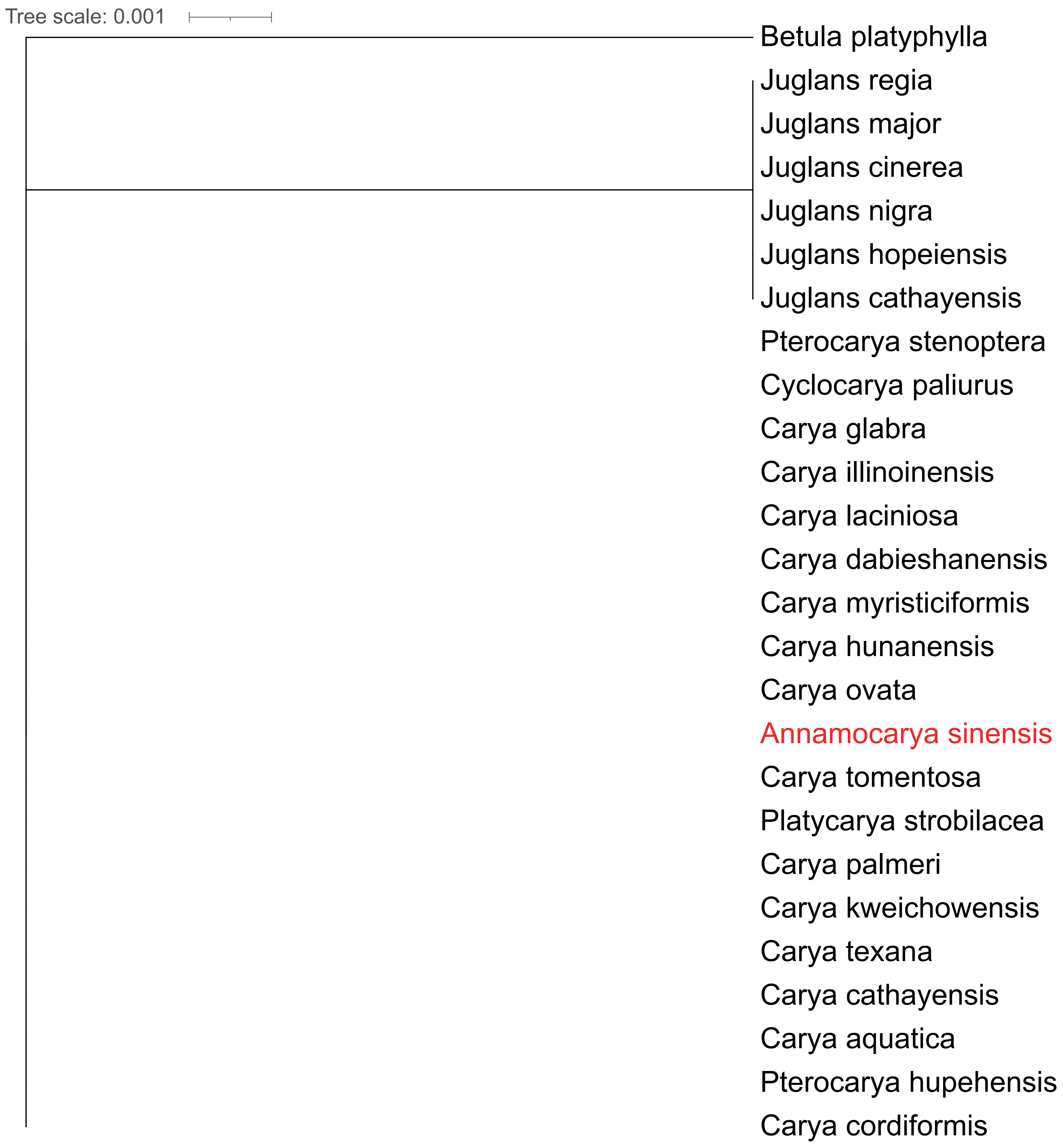

Figure S2A15. Phylogenetic tree constructed using petG

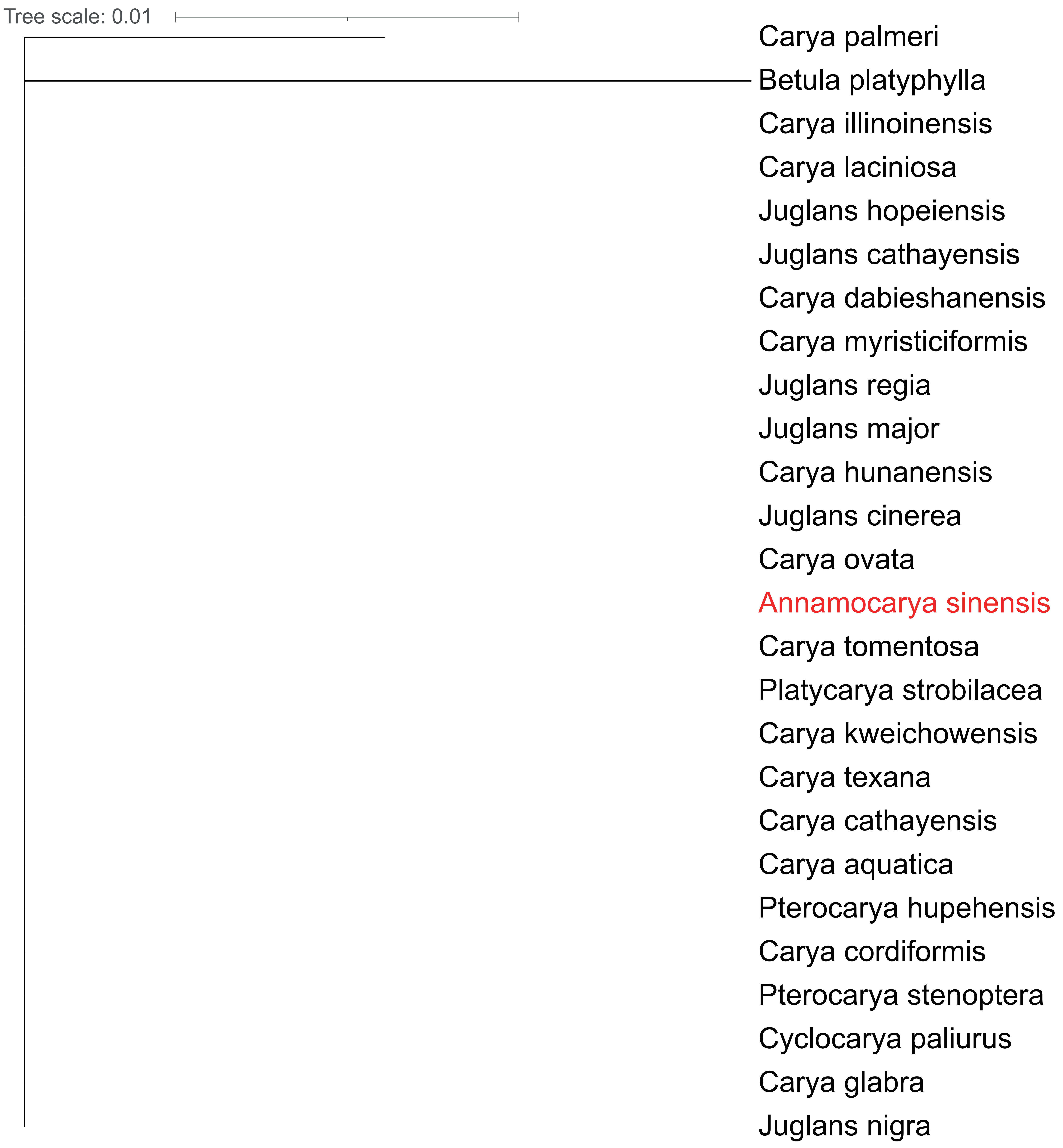

Figure S2A16. Phylogenetic tree constructed using petL

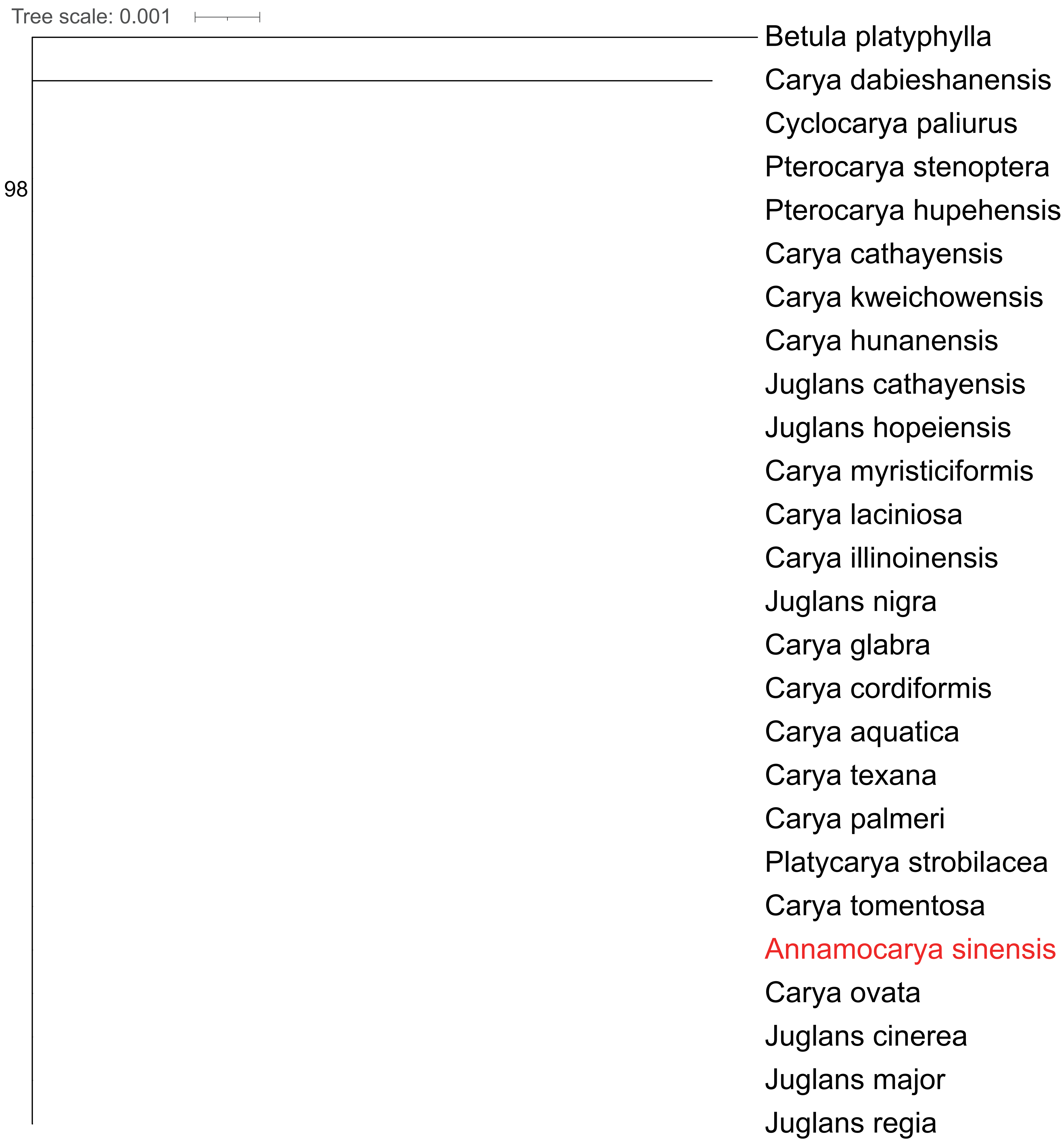

Figure S2A17. Phylogenetic tree constructed using petN

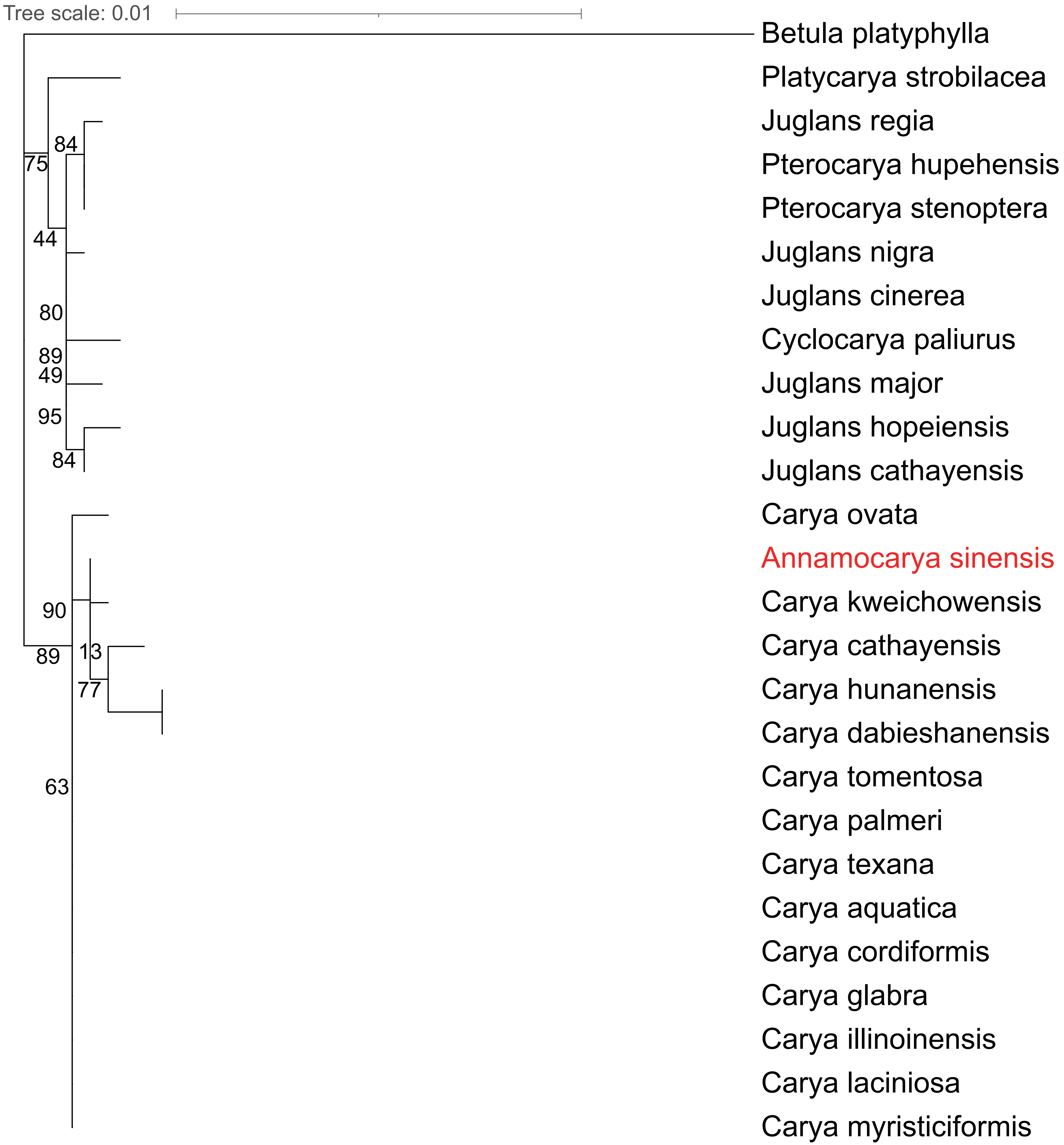

Figure S2A18. Phylogenetic tree constructed using psaA

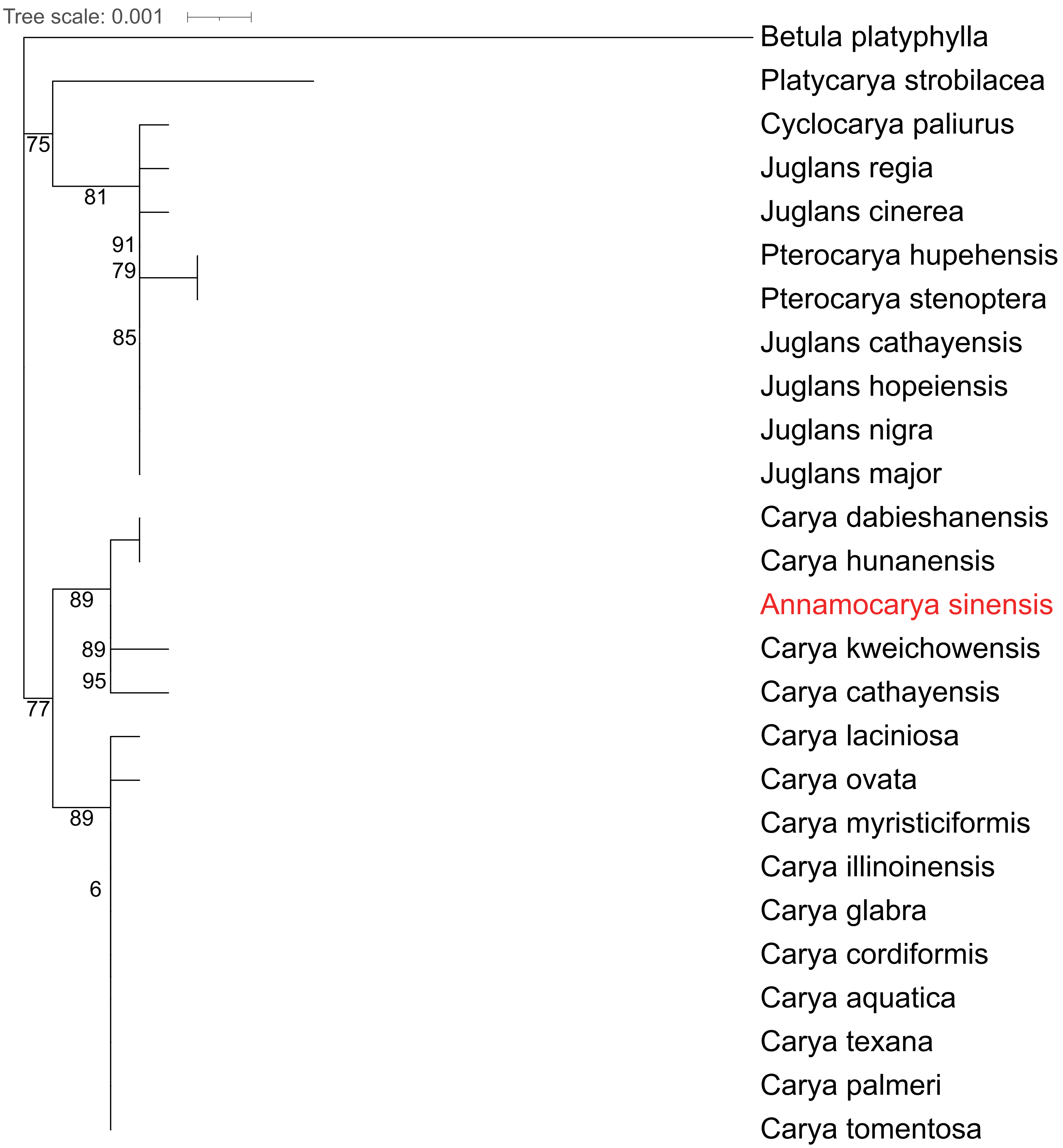

Figure S2A19. Phylogenetic tree constructed using psaB

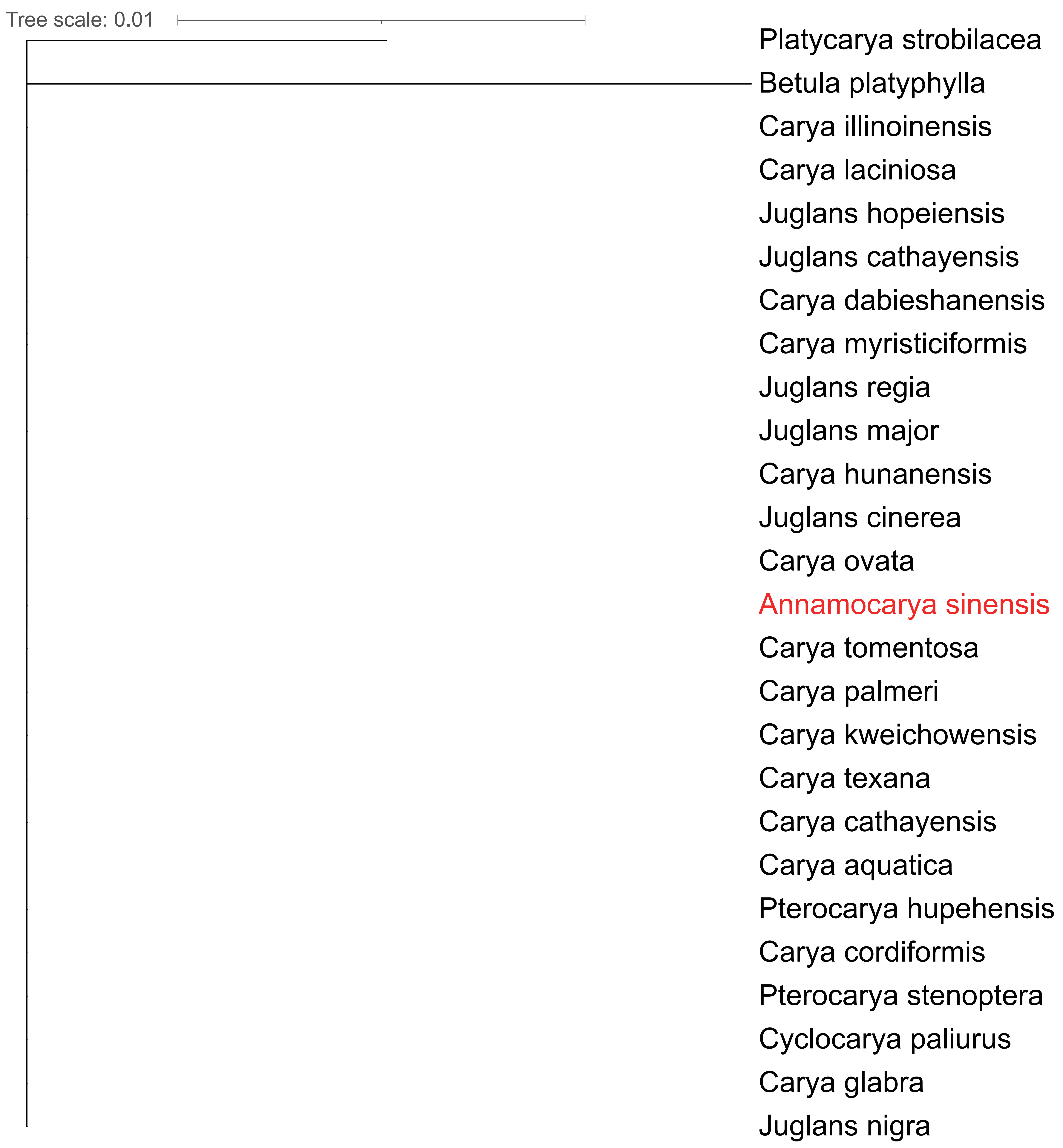

Figure S2A20. Phylogenetic tree constructed using psaI

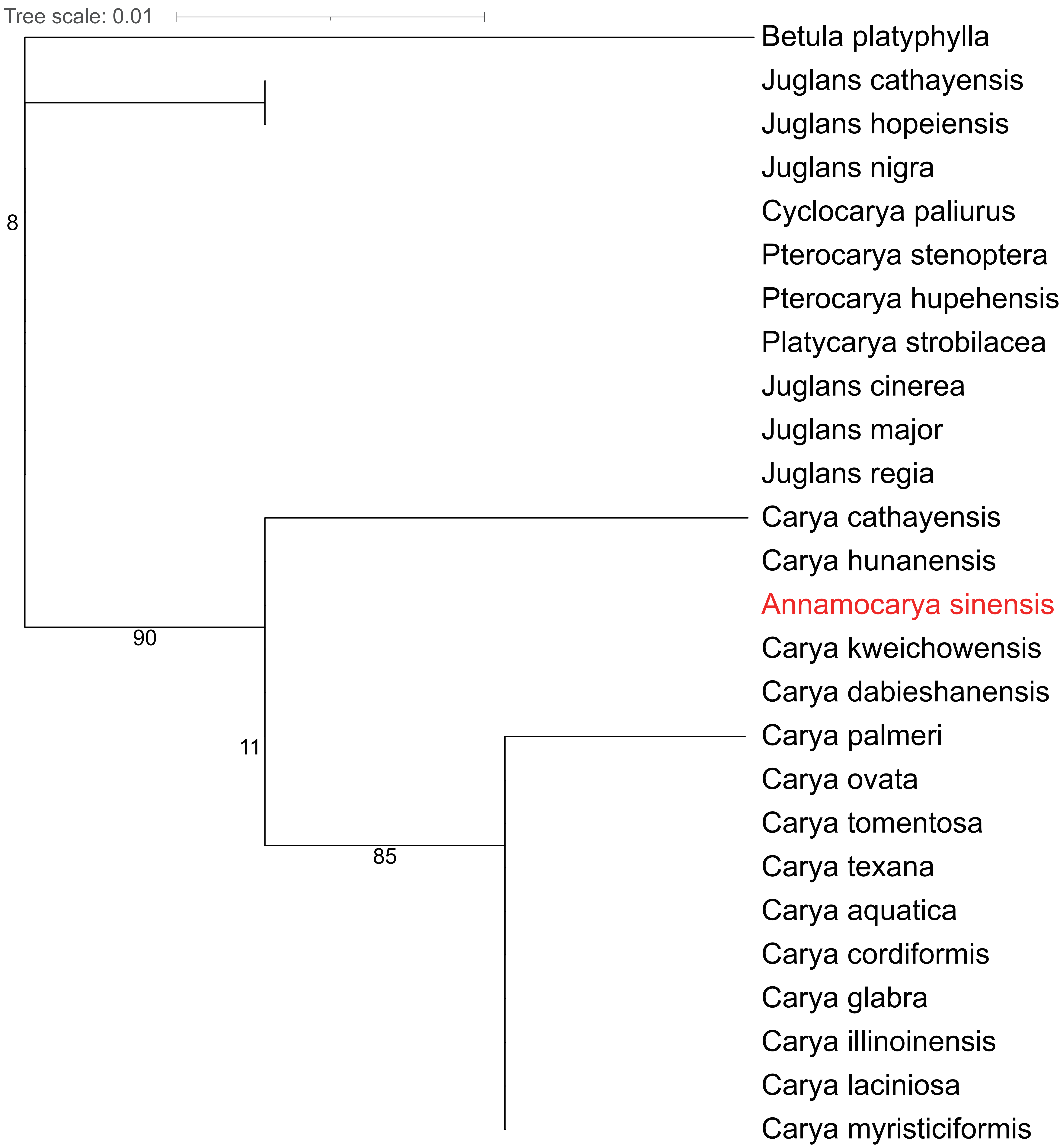

Figure S2A21. Phylogenetic tree constructed using psaJ

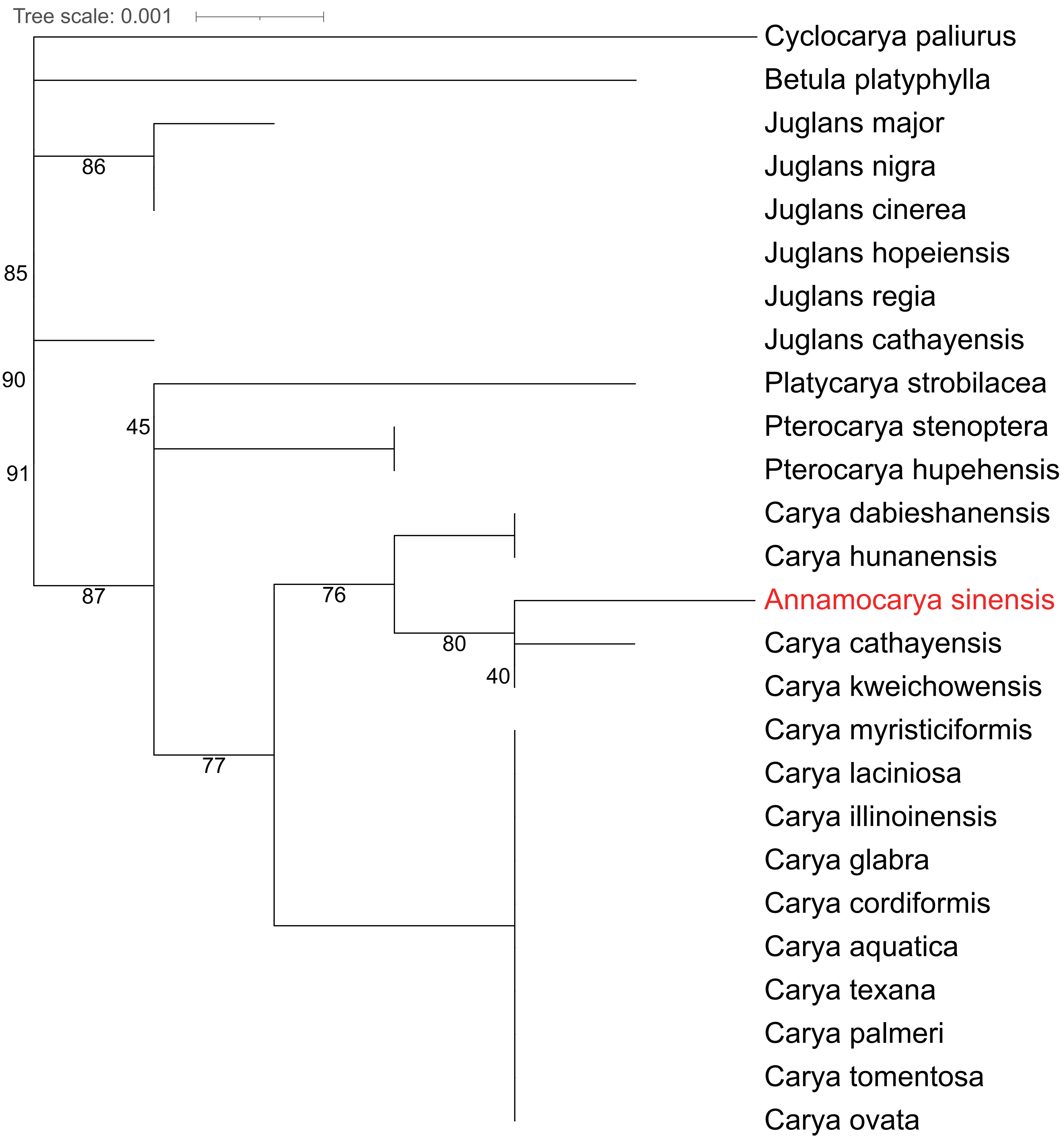

Figure S2A22. Phylogenetic tree constructed using psbA

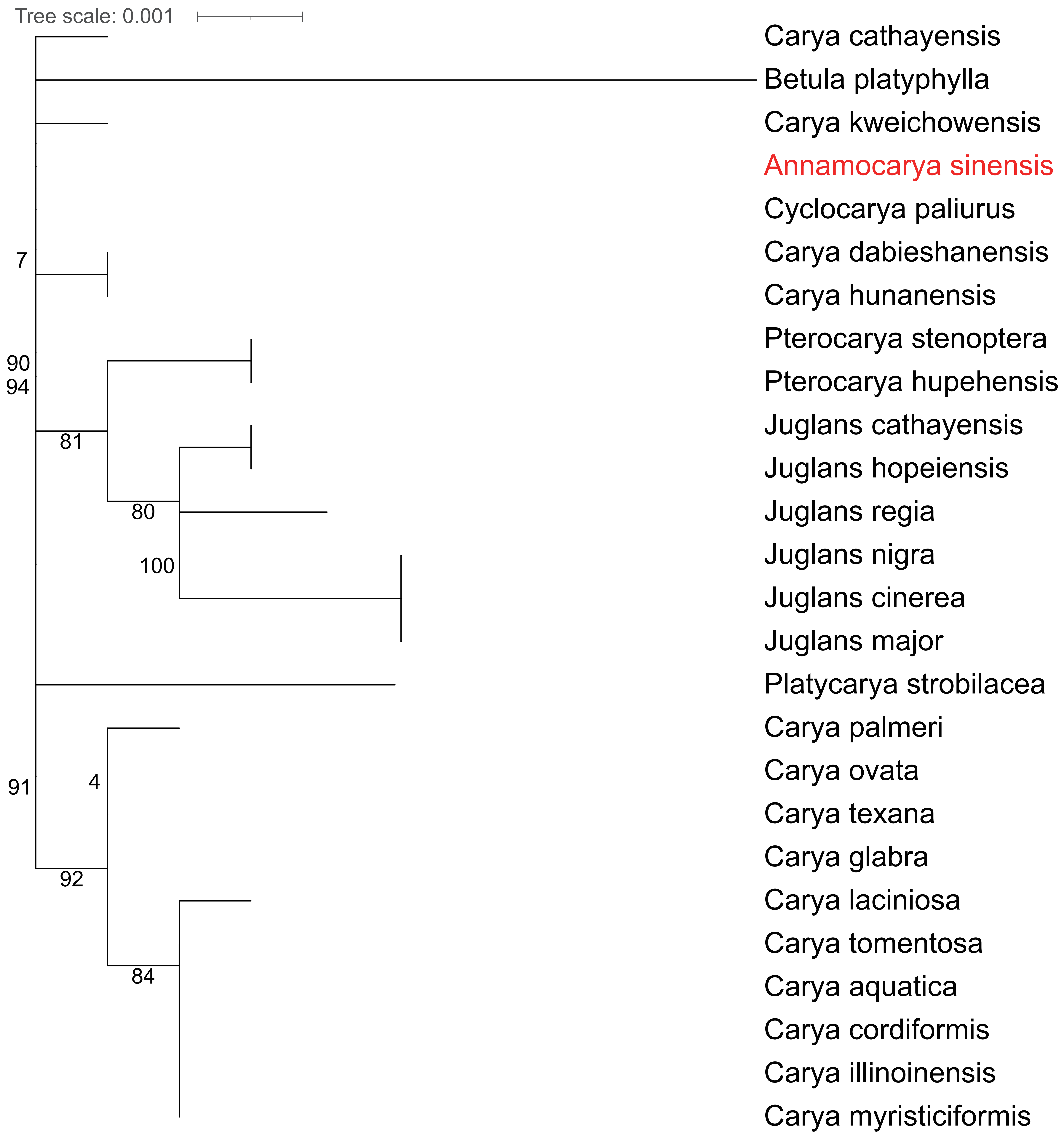

Figure S2A23. Phylogenetic tree constructed using psbC

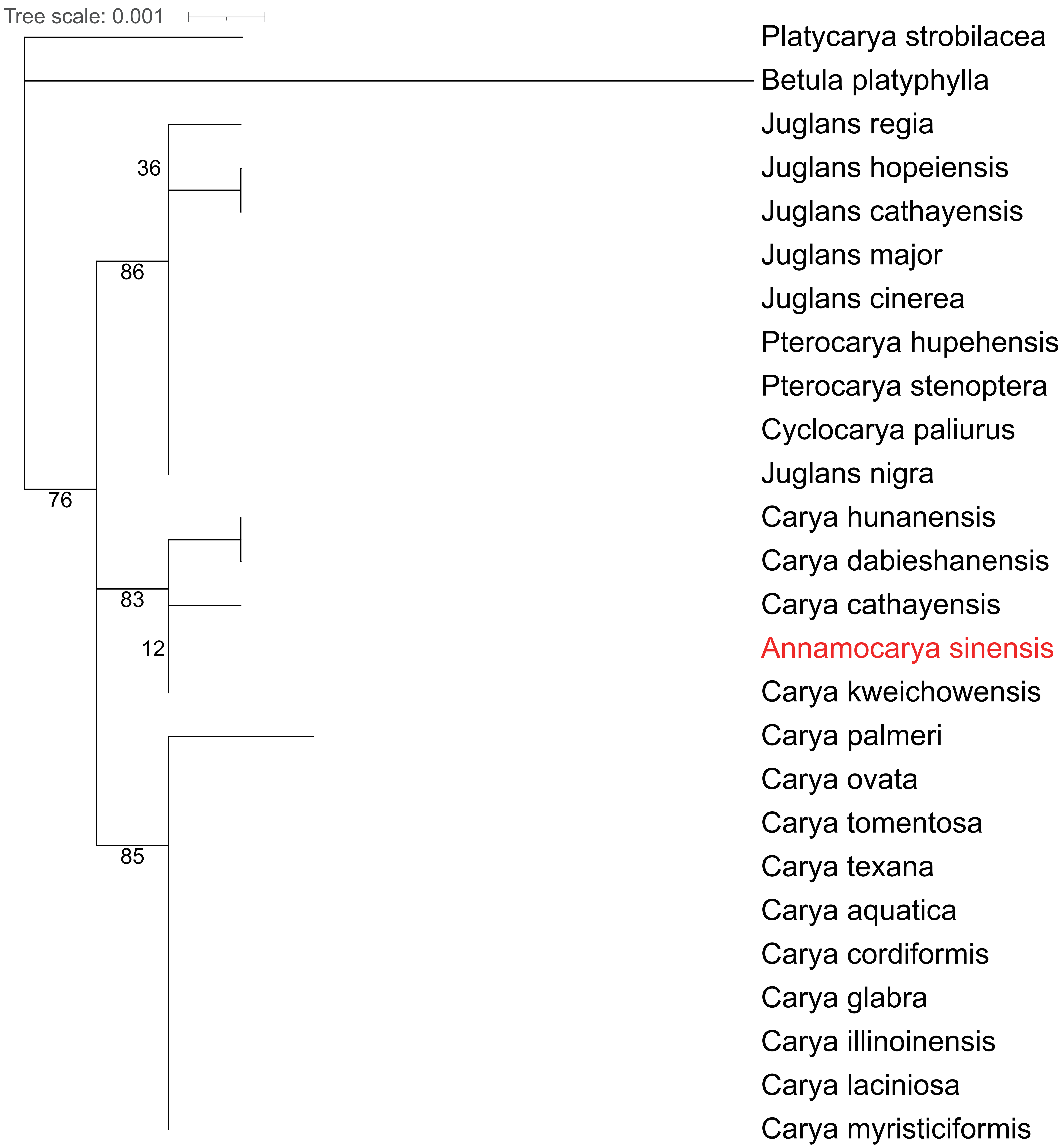

Figure S2A24. Phylogenetic tree constructed using psbD

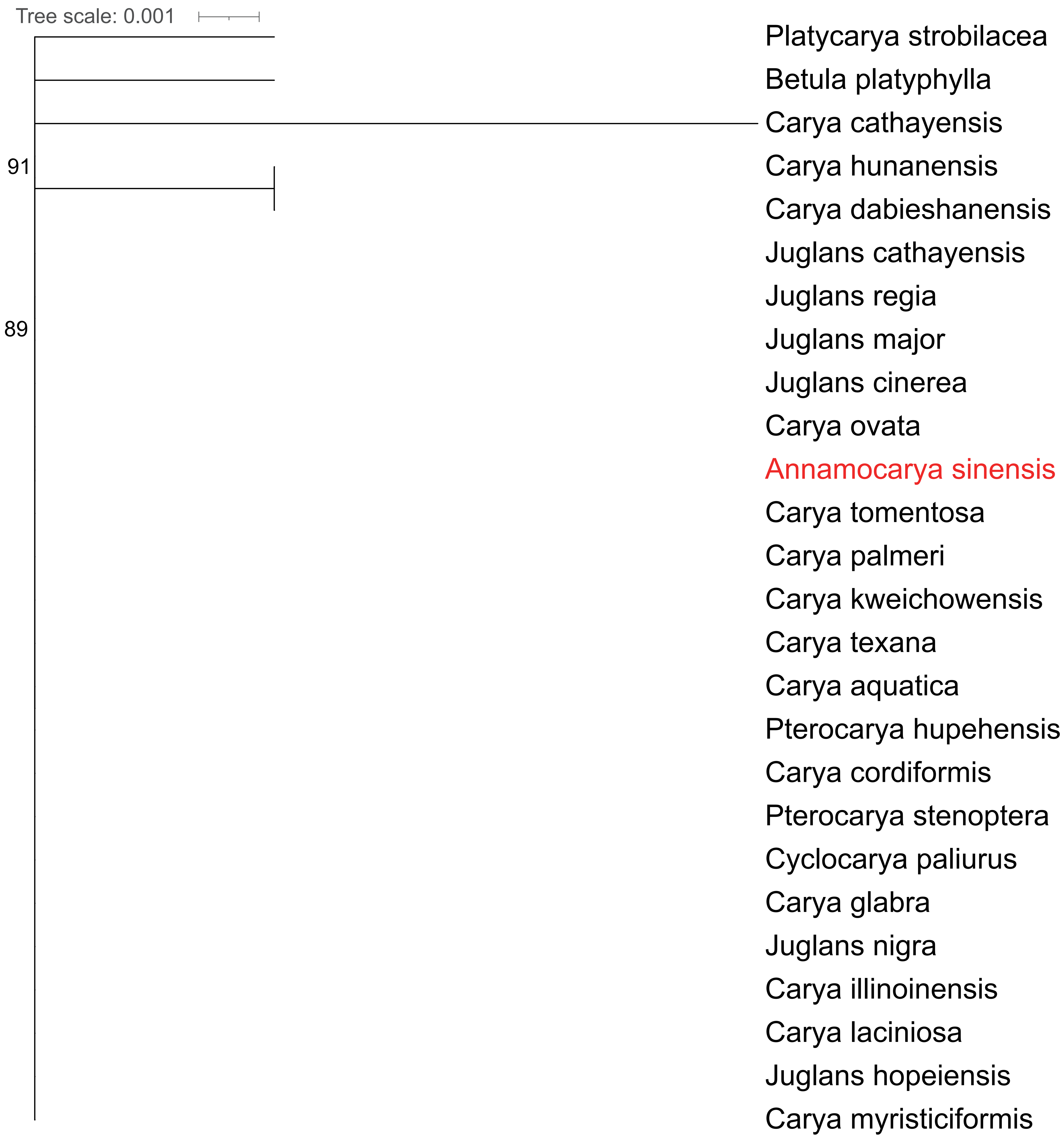

Figure S2A25. Phylogenetic tree constructed using psbE

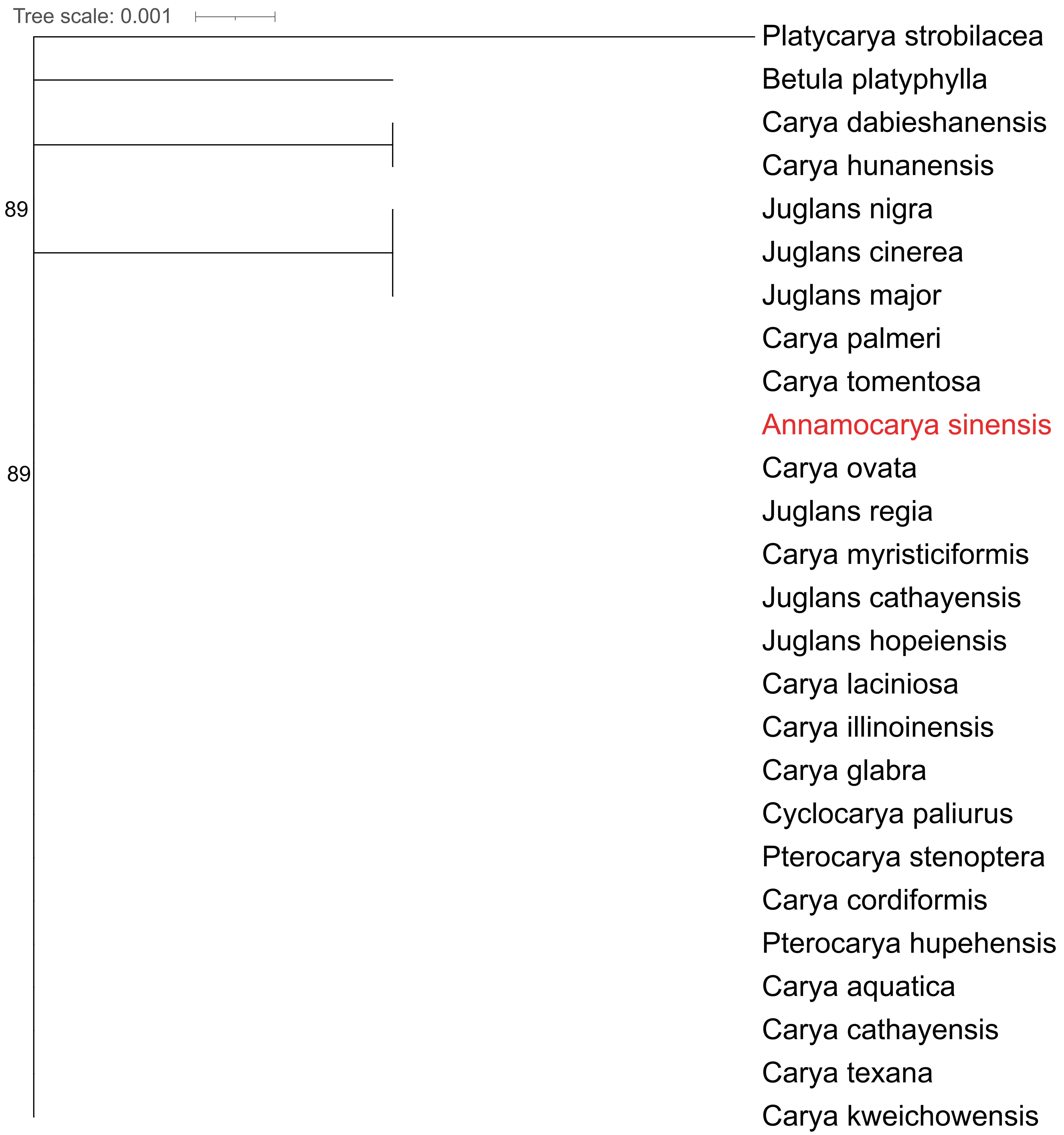

Figure S2A26. Phylogenetic tree constructed using psbH

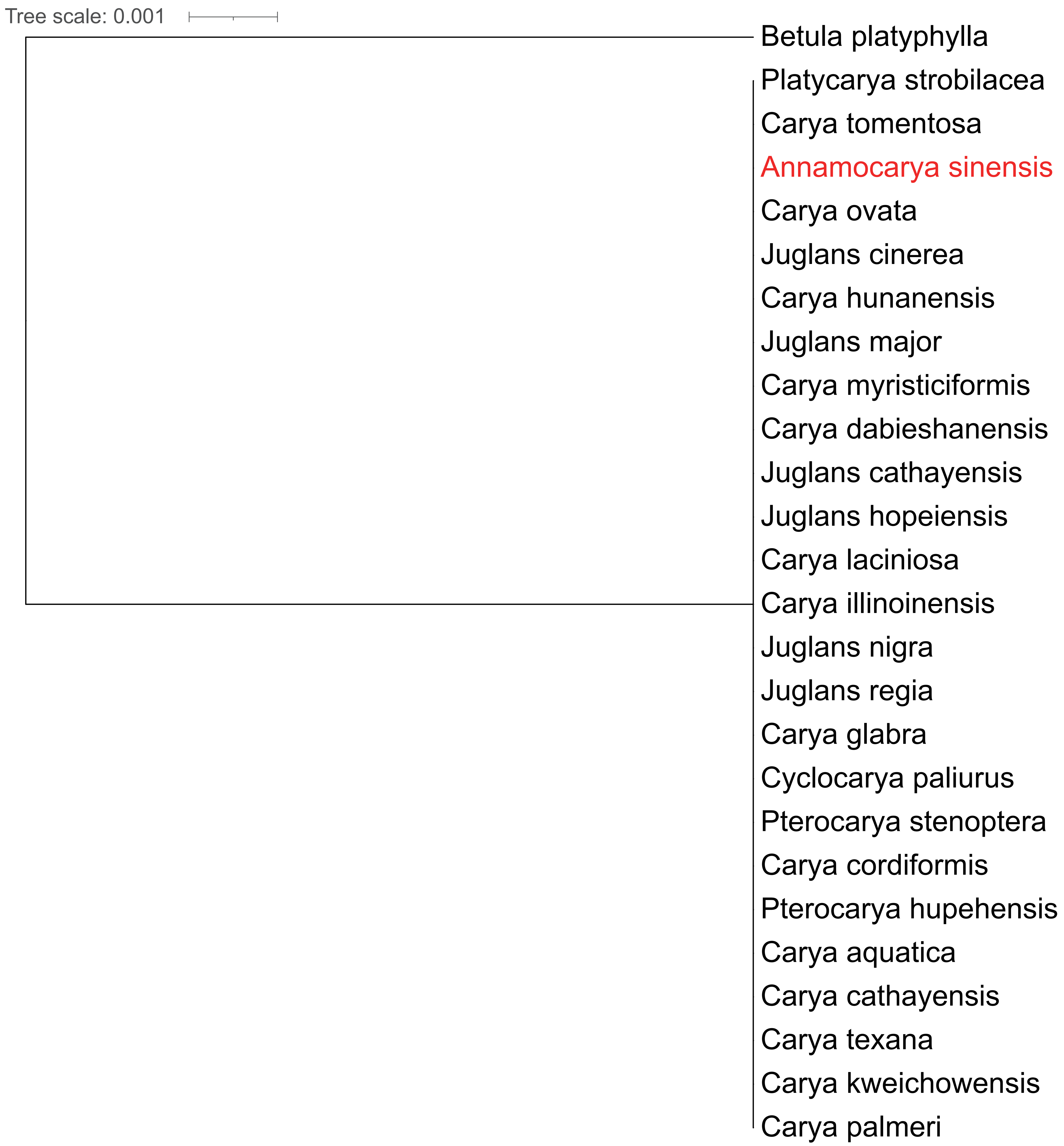

Figure S2A27. Phylogenetic tree constructed using psbJ

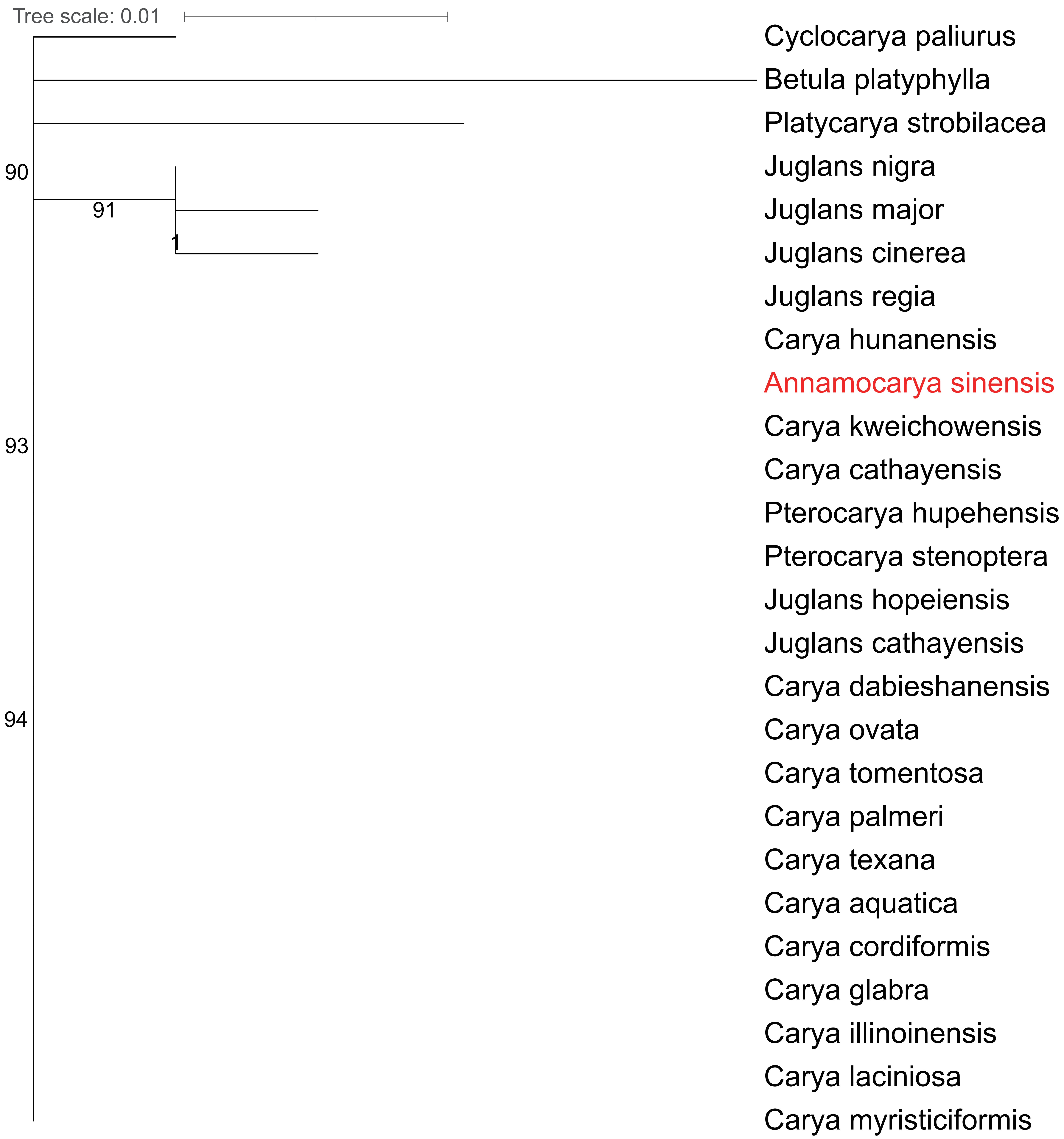

Figure S2A28. Phylogenetic tree constructed using psbK

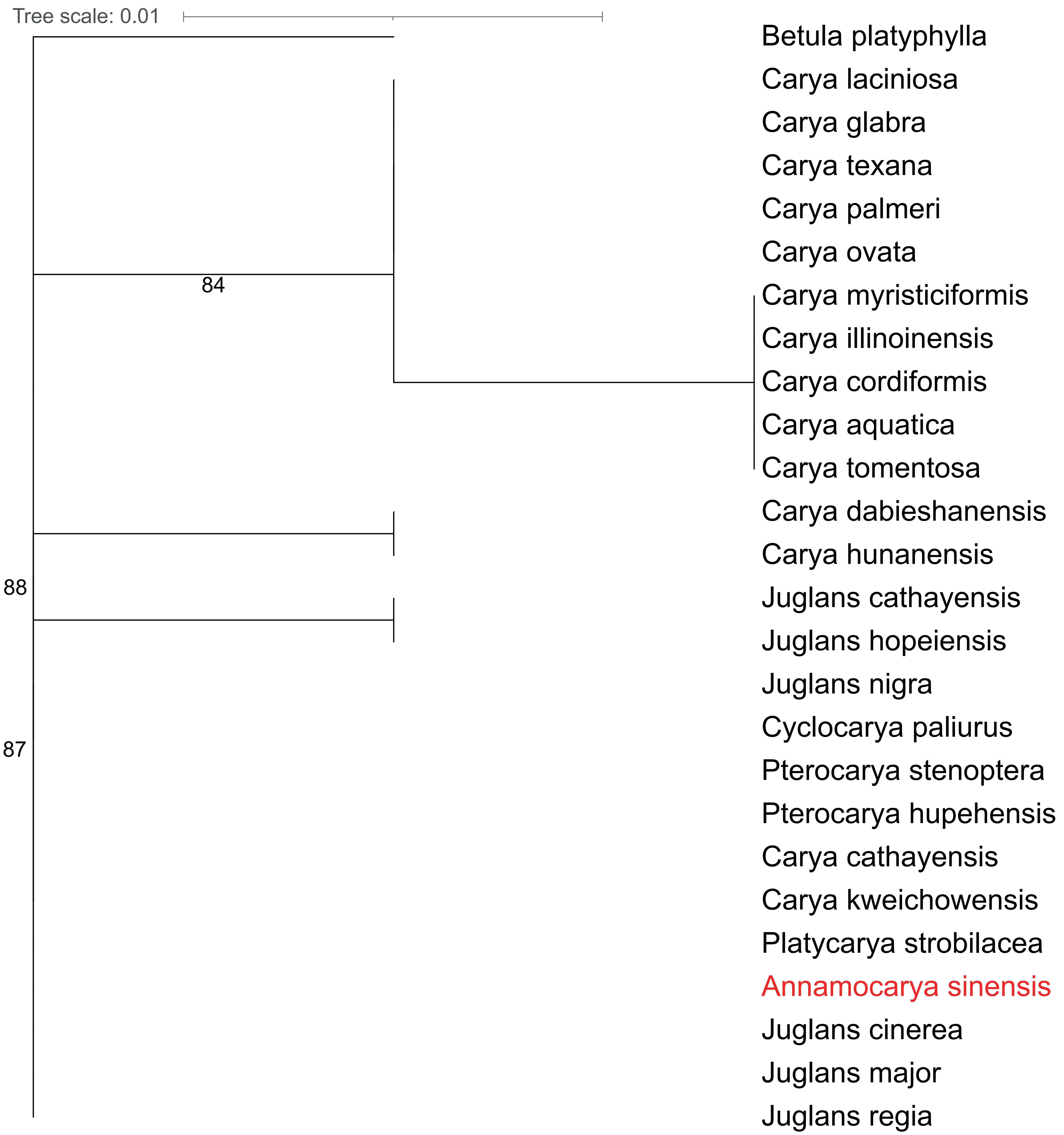

Figure S2A29. Phylogenetic tree constructed using psbL

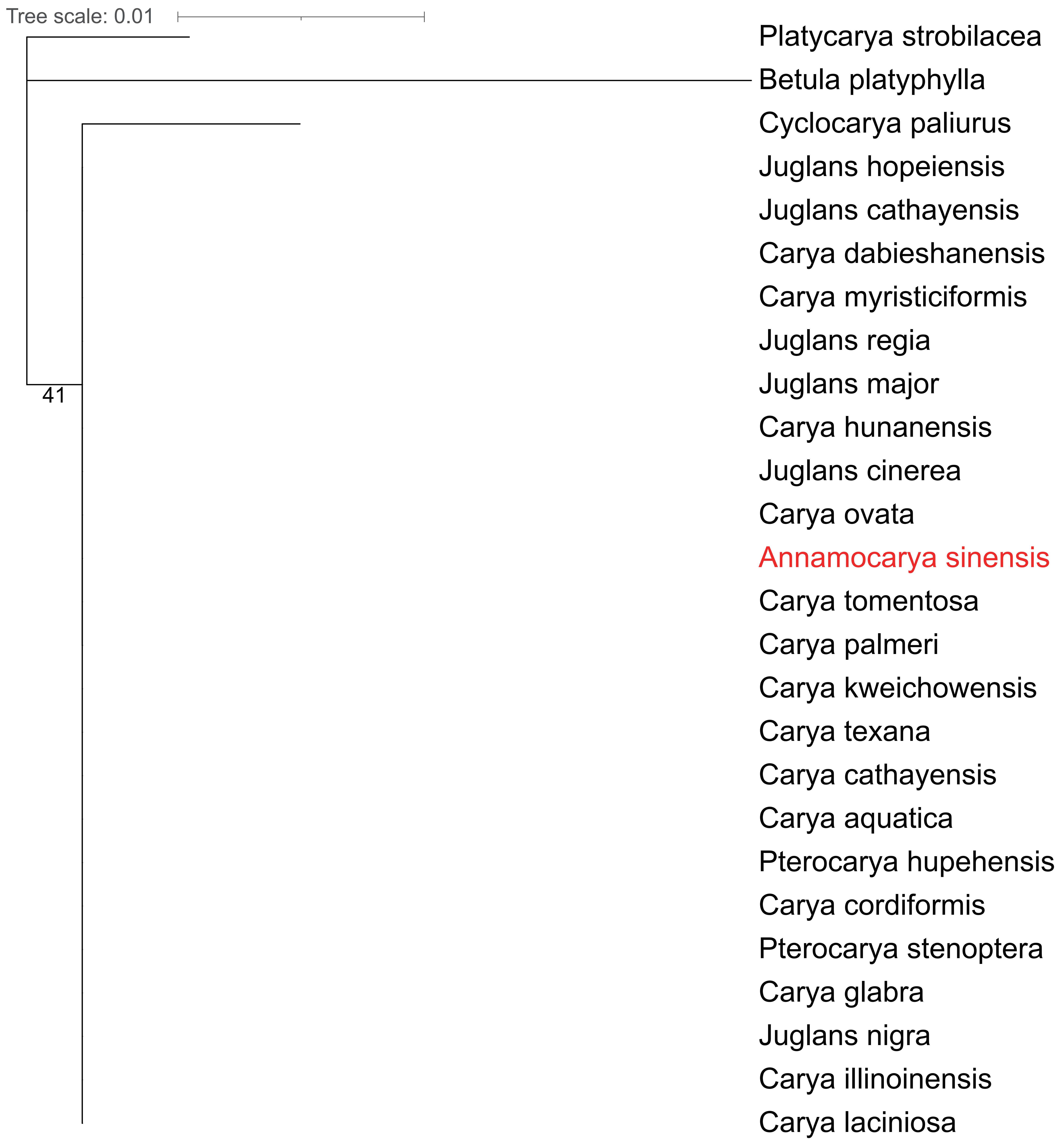

Figure S2A30. Phylogenetic tree constructed using psbM

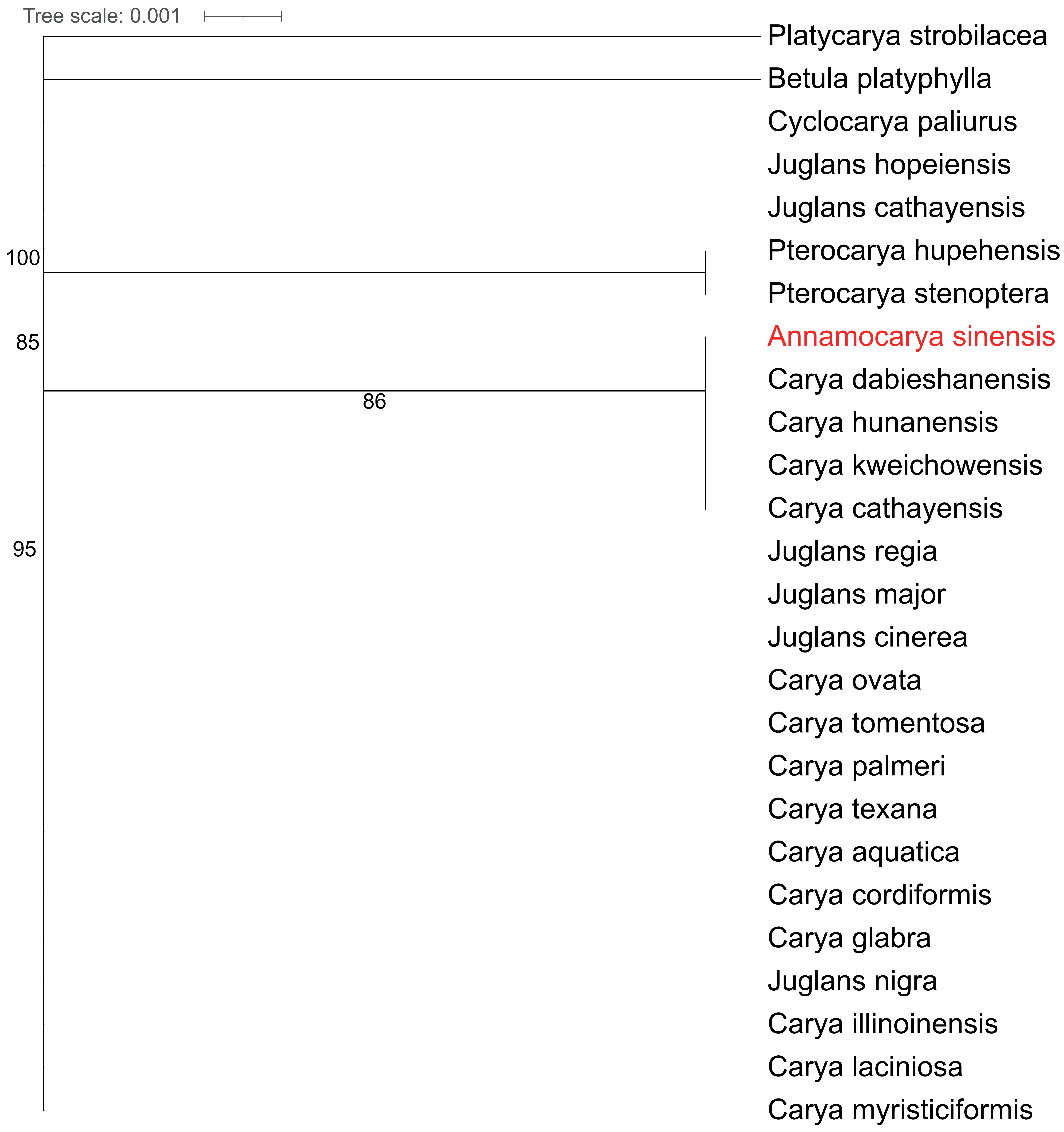

Figure S2A31. Phylogenetic tree constructed using psbT

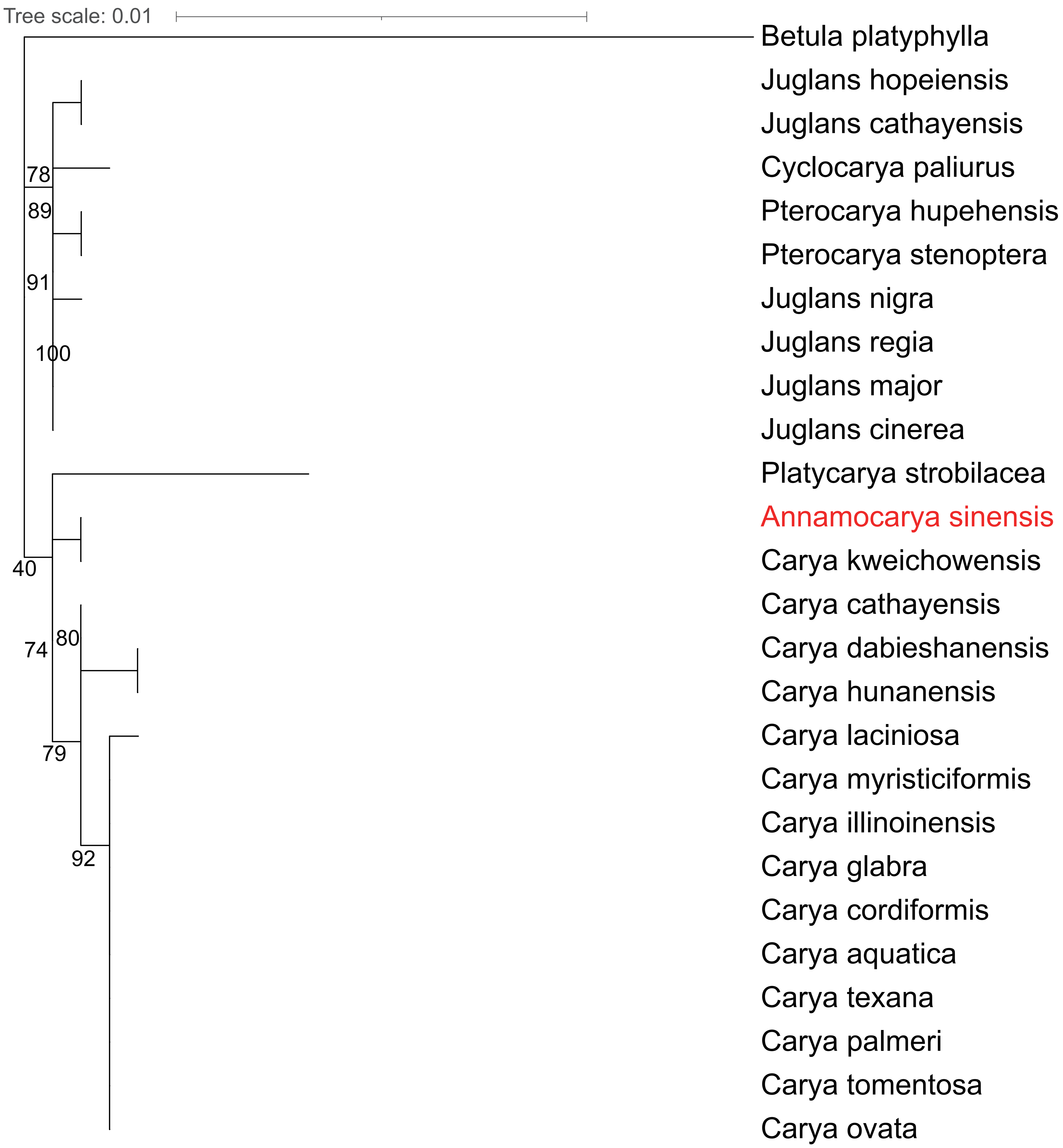

Figure S2A32. Phylogenetic tree constructed using rbcL

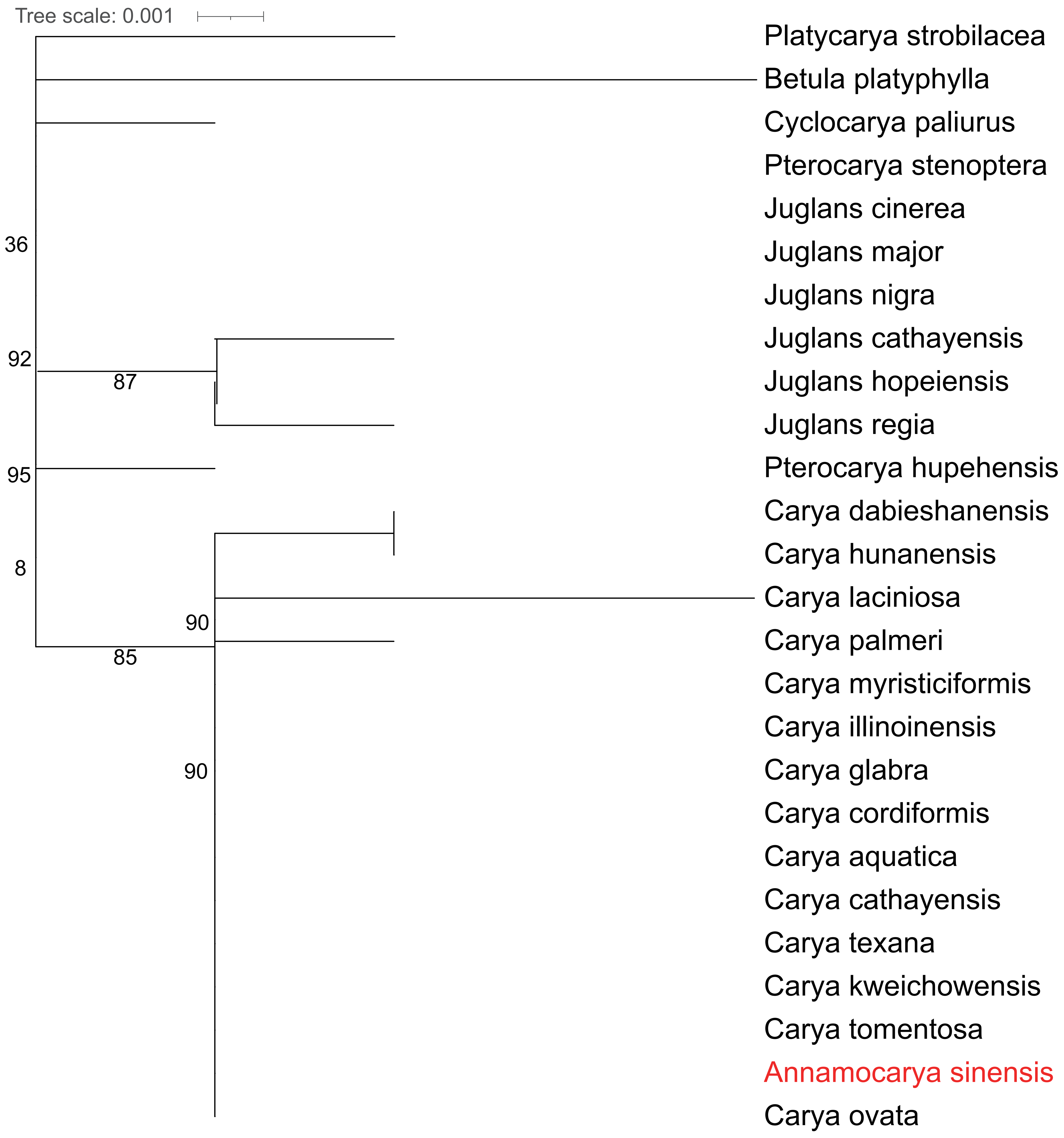

Figure S2A33. Phylogenetic tree constructed using rpl14

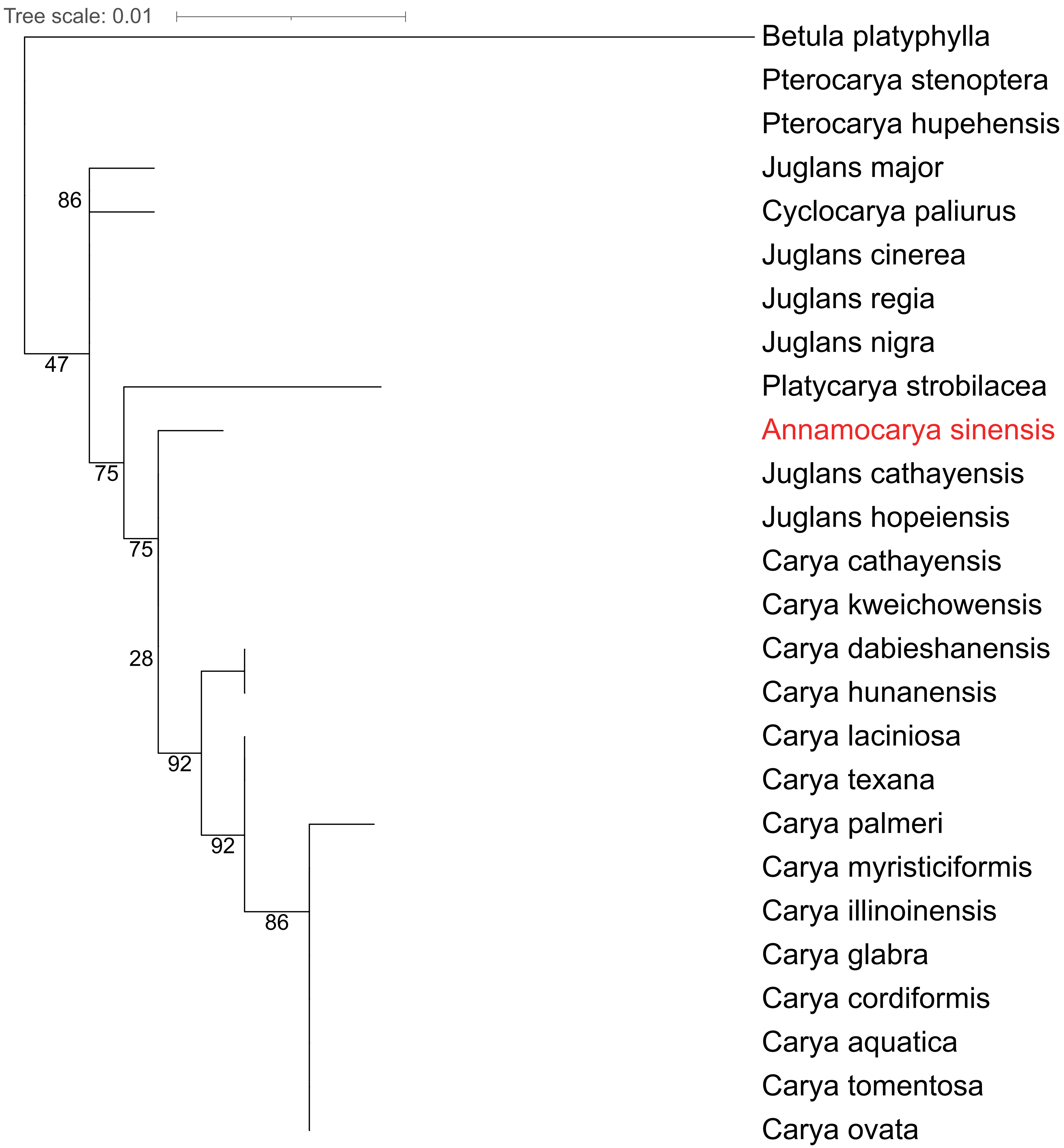

Figure S2A34. Phylogenetic tree constructed using rpl20

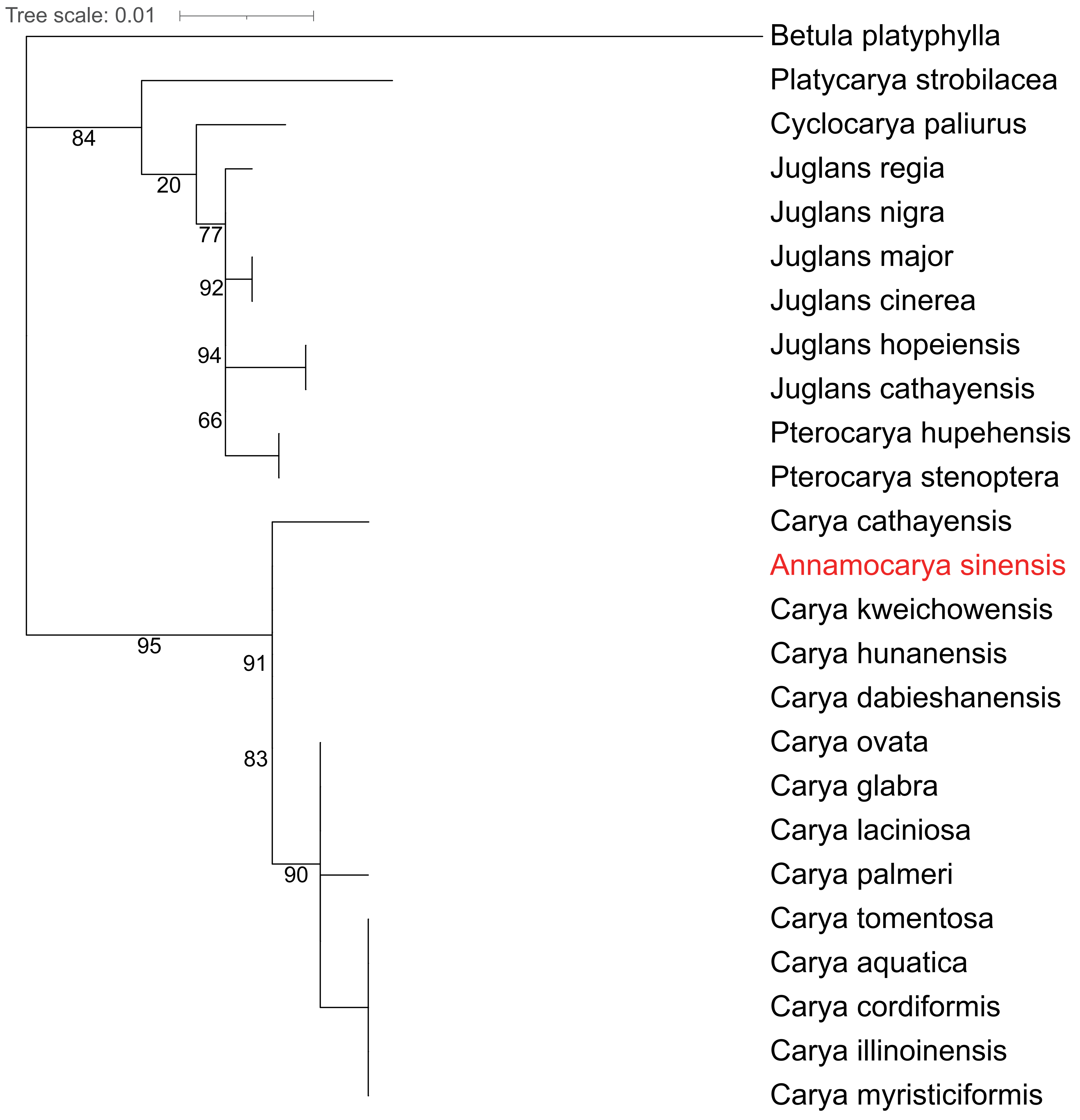

Figure S2A35. Phylogenetic tree constructed using rpl22

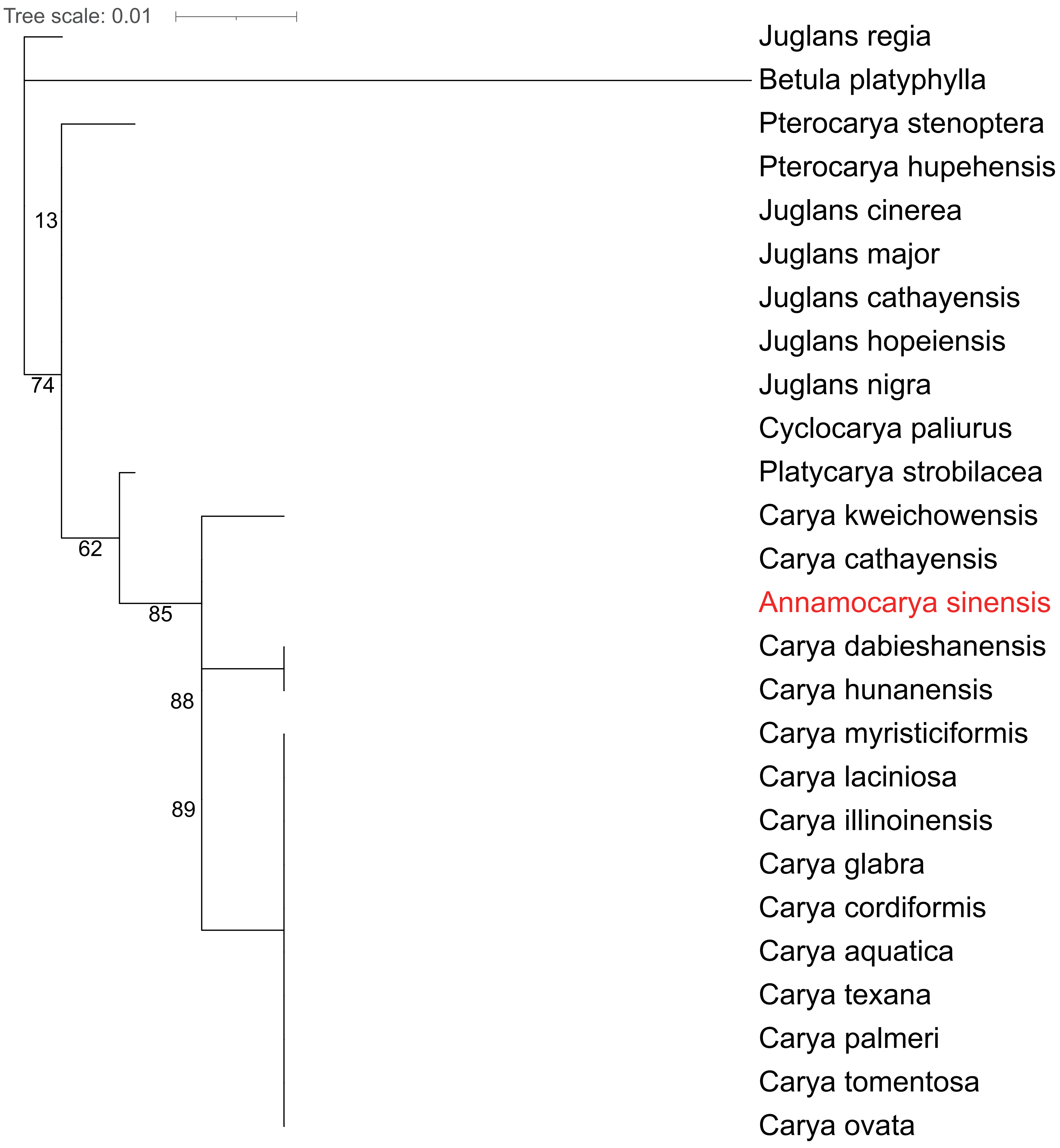

Figure S2A36. Phylogenetic tree constructed using rpl32

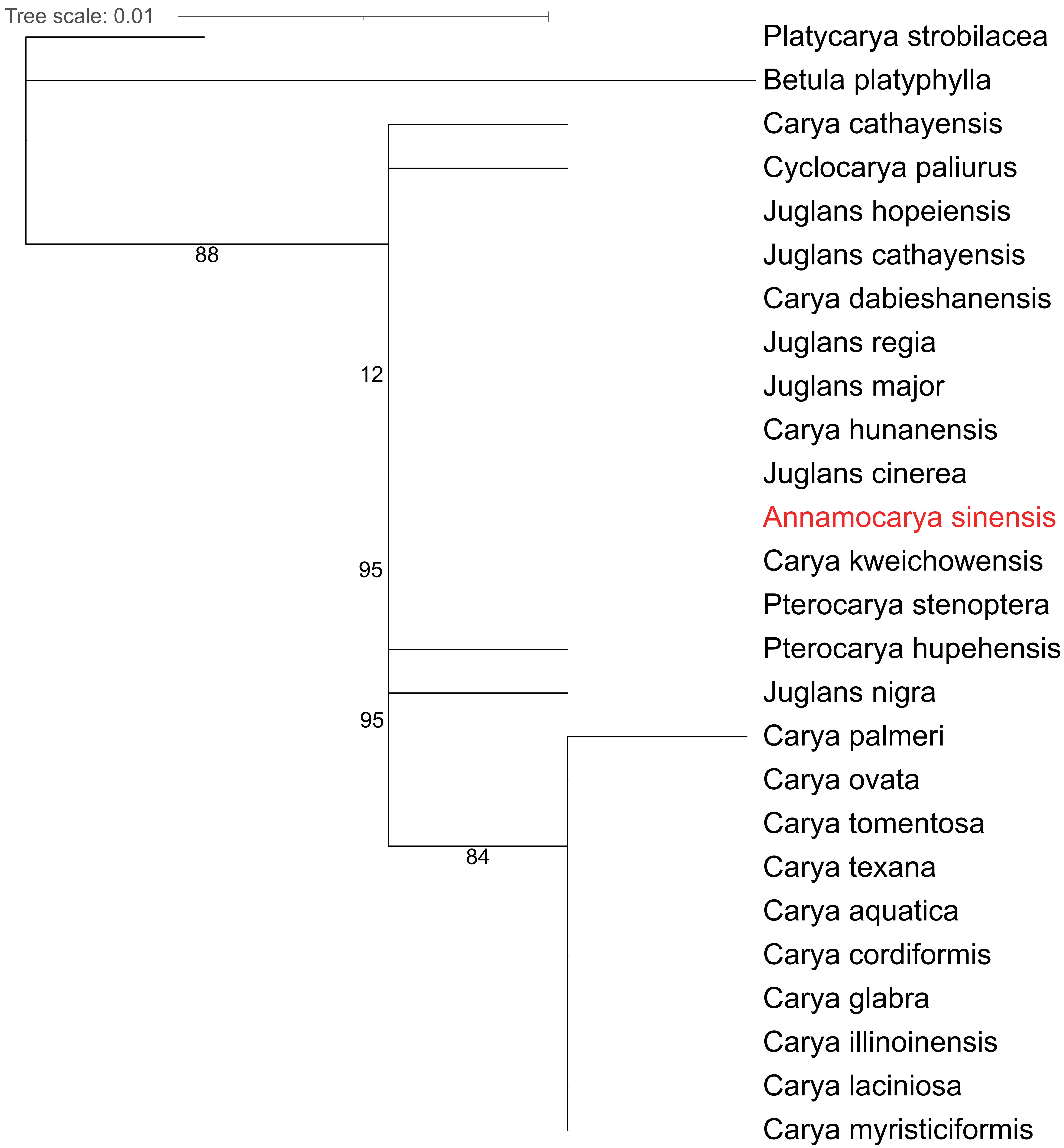

Figure S2A37. Phylogenetic tree constructed using rpl33

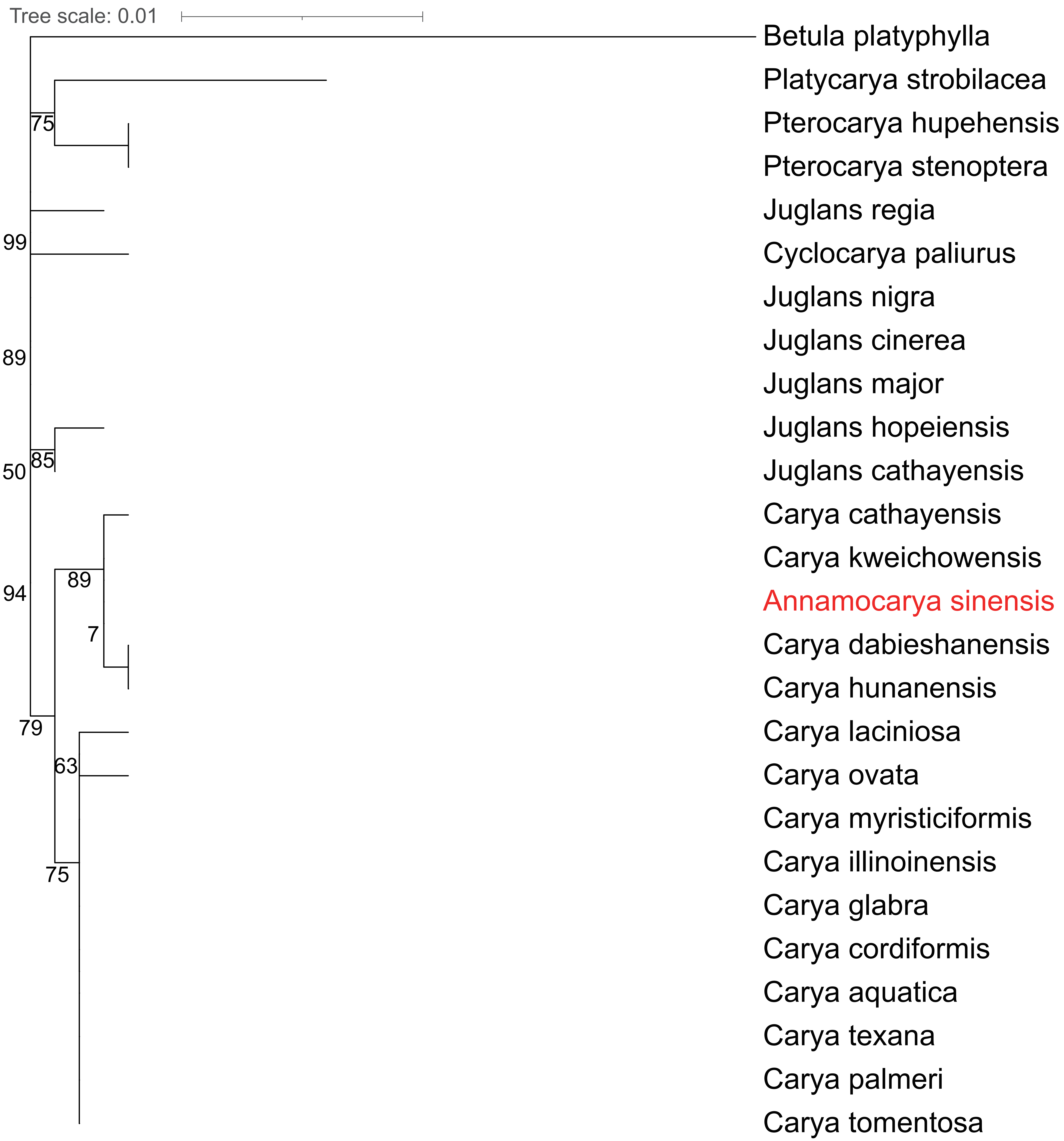

Figure S2A38. Phylogenetic tree constructed using rpoA

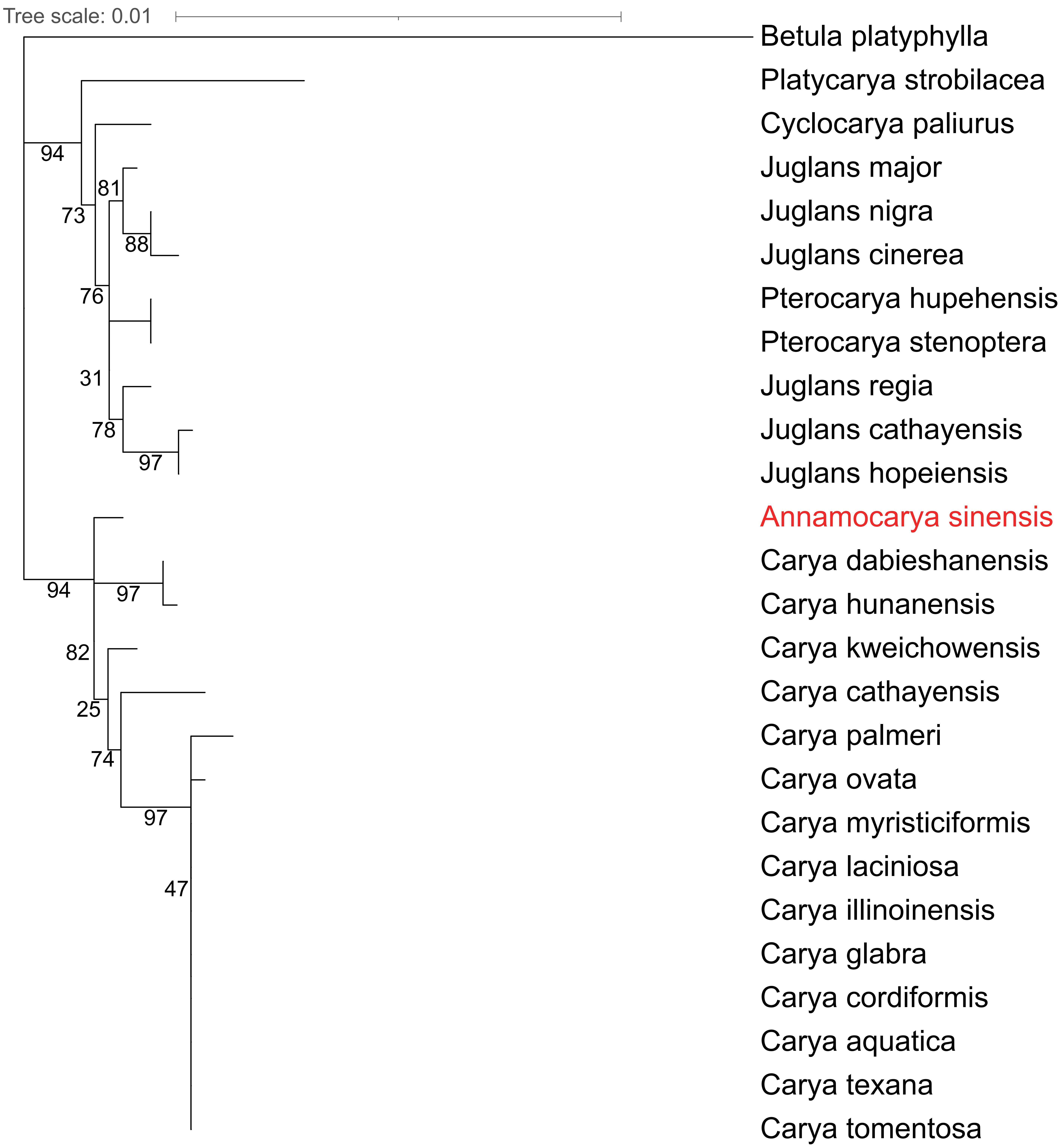

Figure S2A39. Phylogenetic tree constructed using rpoB

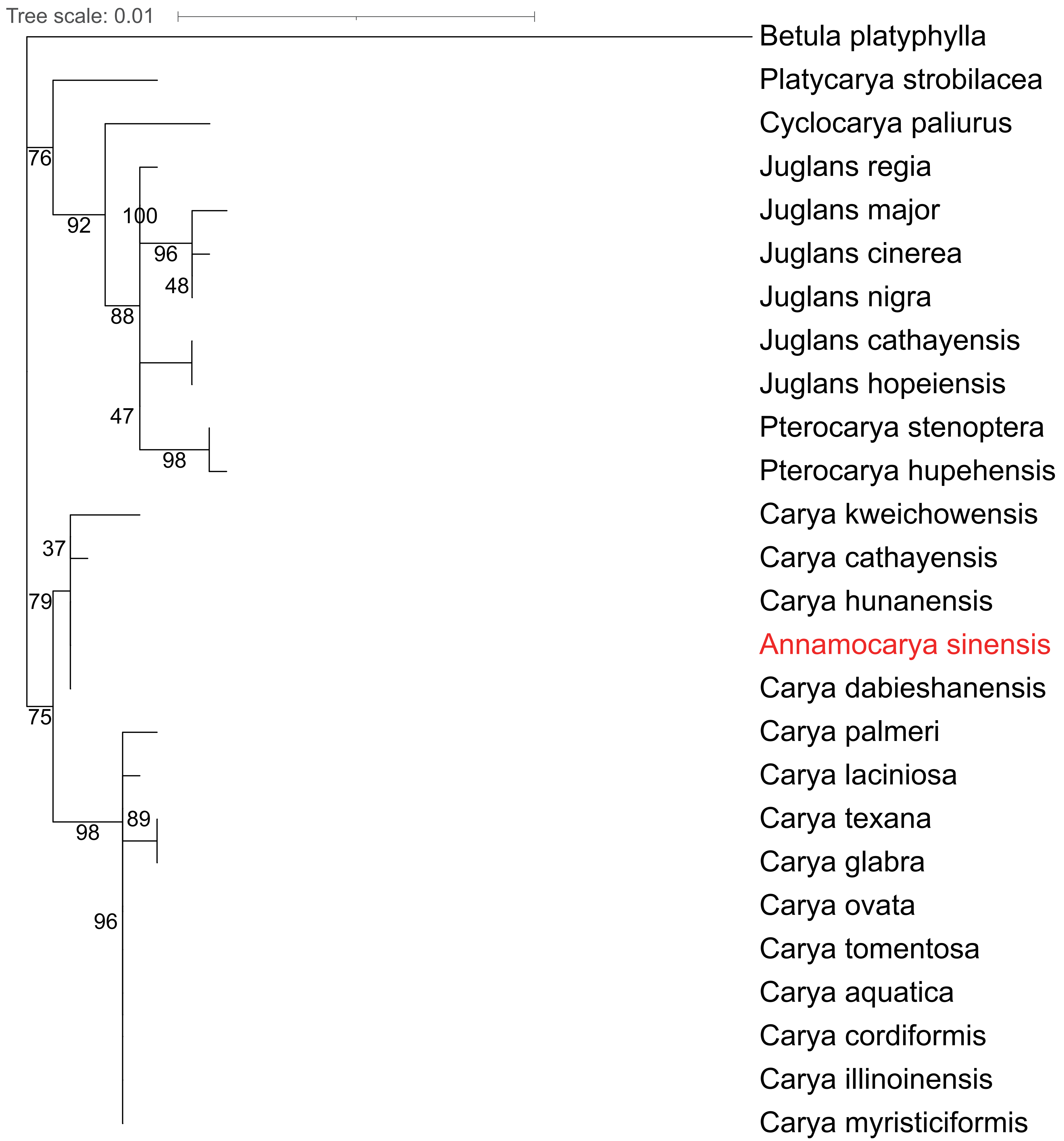

Figure S2A40. Phylogenetic tree constructed using rpoC1

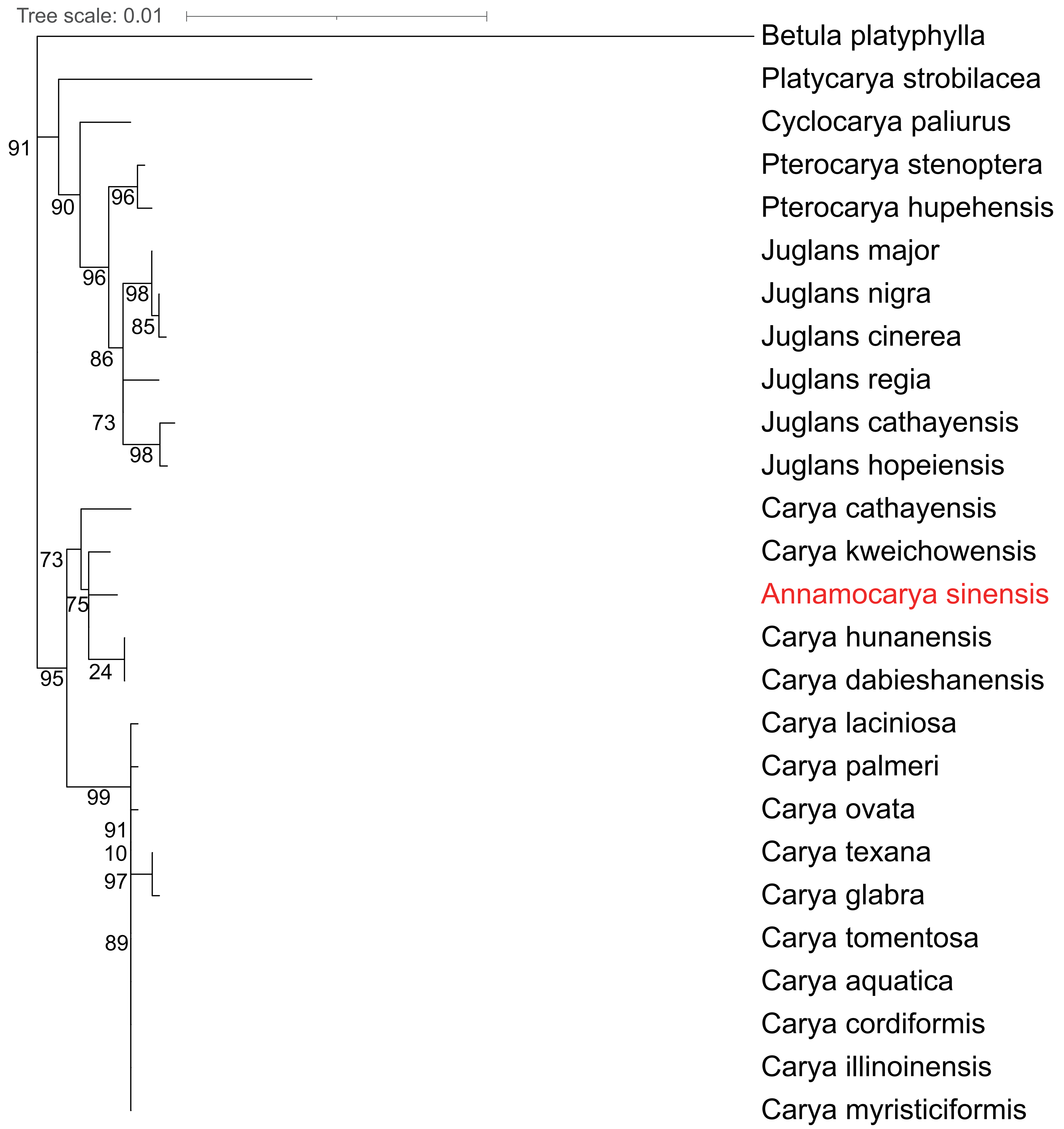

Figure S2A41. Phylogenetic tree constructed using rpoC2

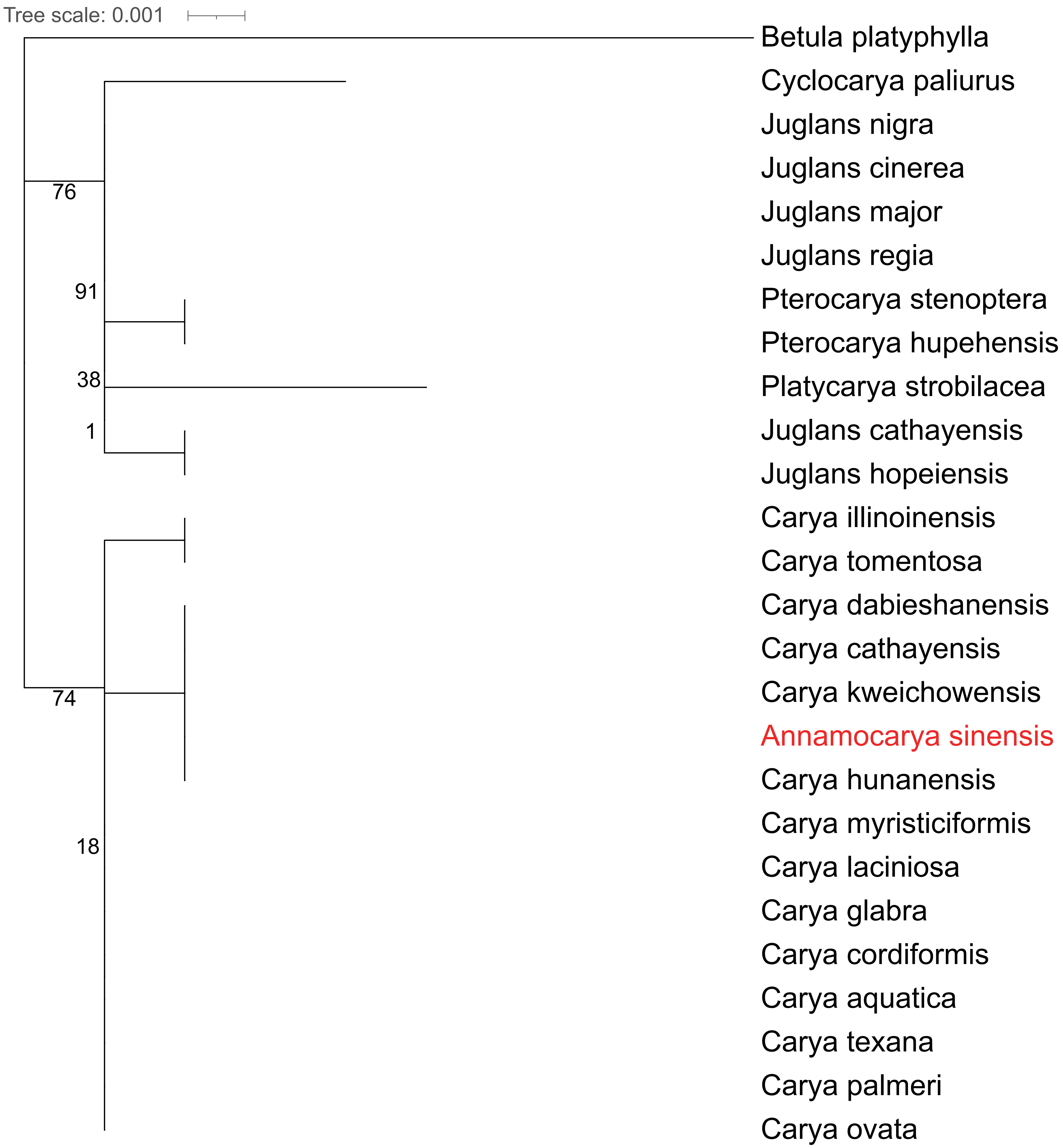

Figure S2A42. Phylogenetic tree constructed using rps11

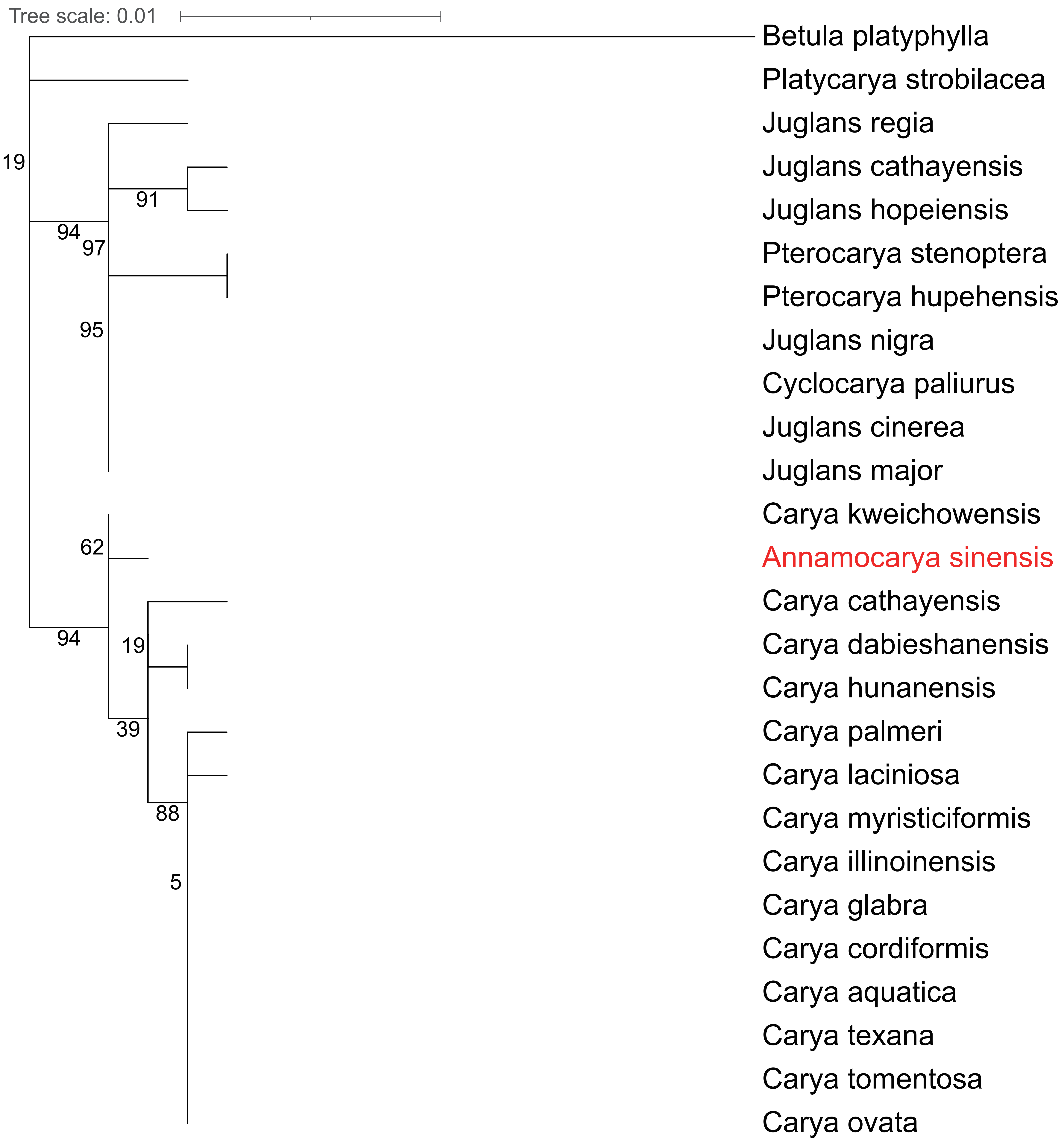

Figure S2A43. Phylogenetic tree constructed using rps14

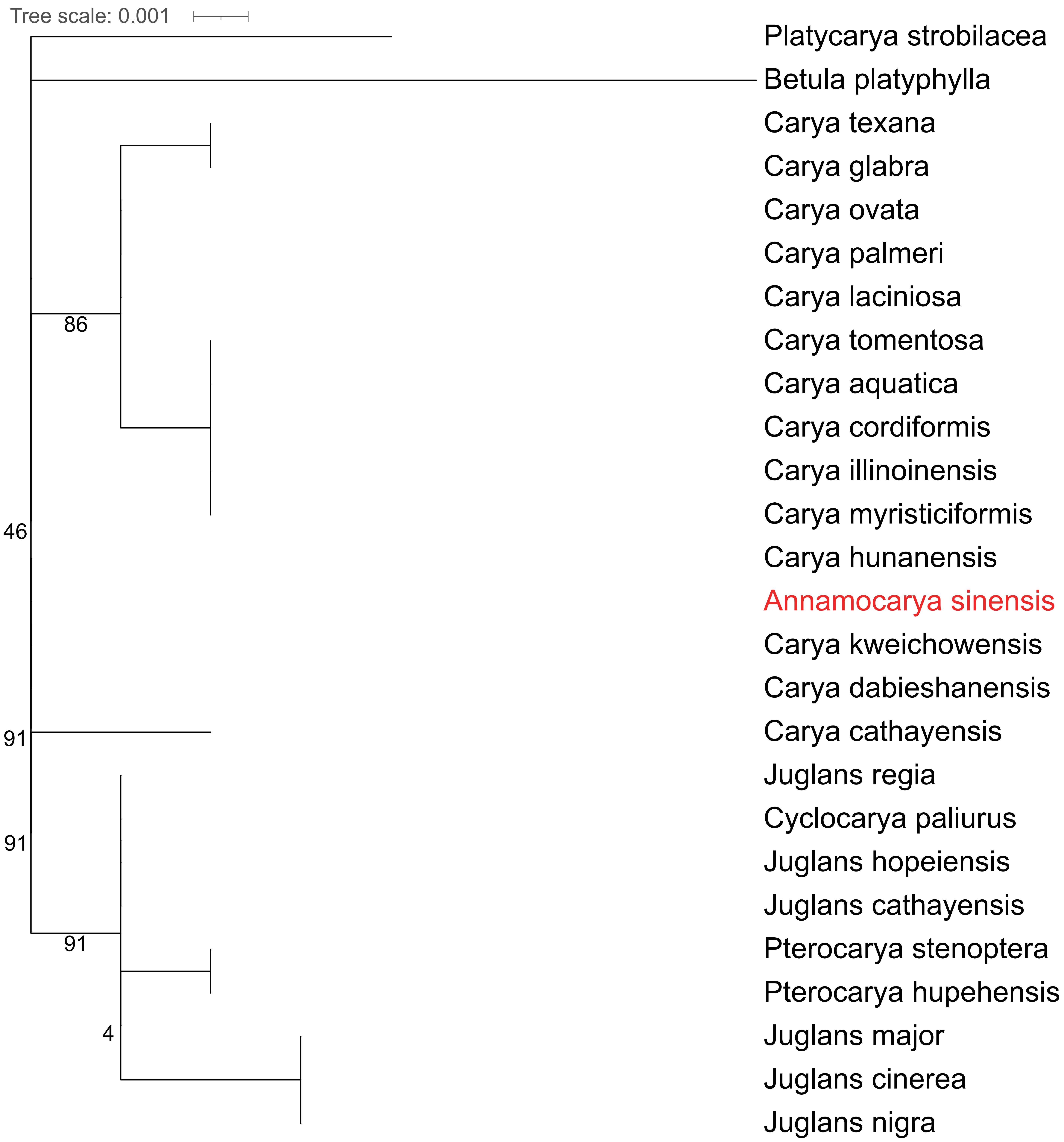

Figure S2A44. Phylogenetic tree constructed using rps18

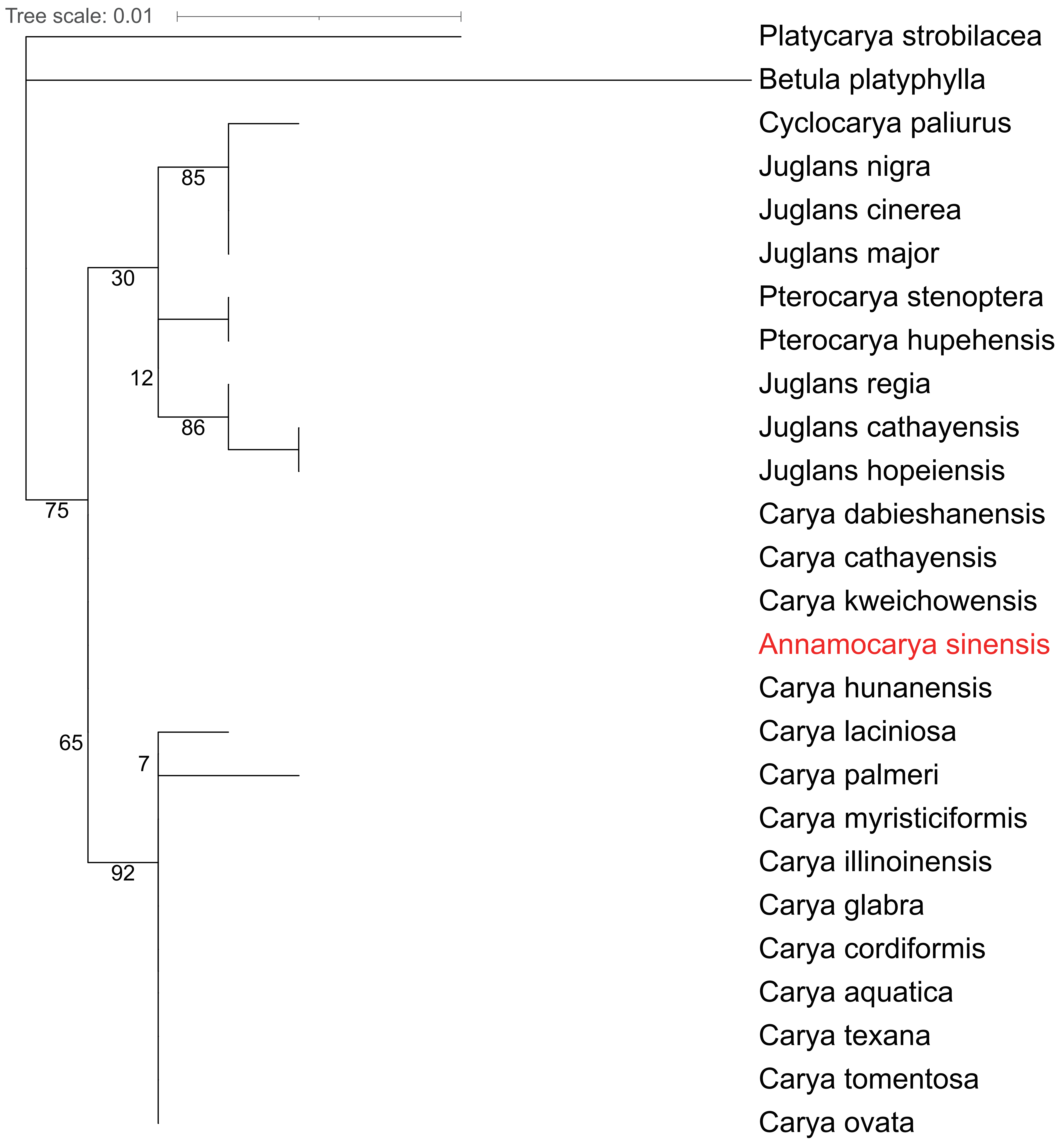

Figure S2A45. Phylogenetic tree constructed using rps2

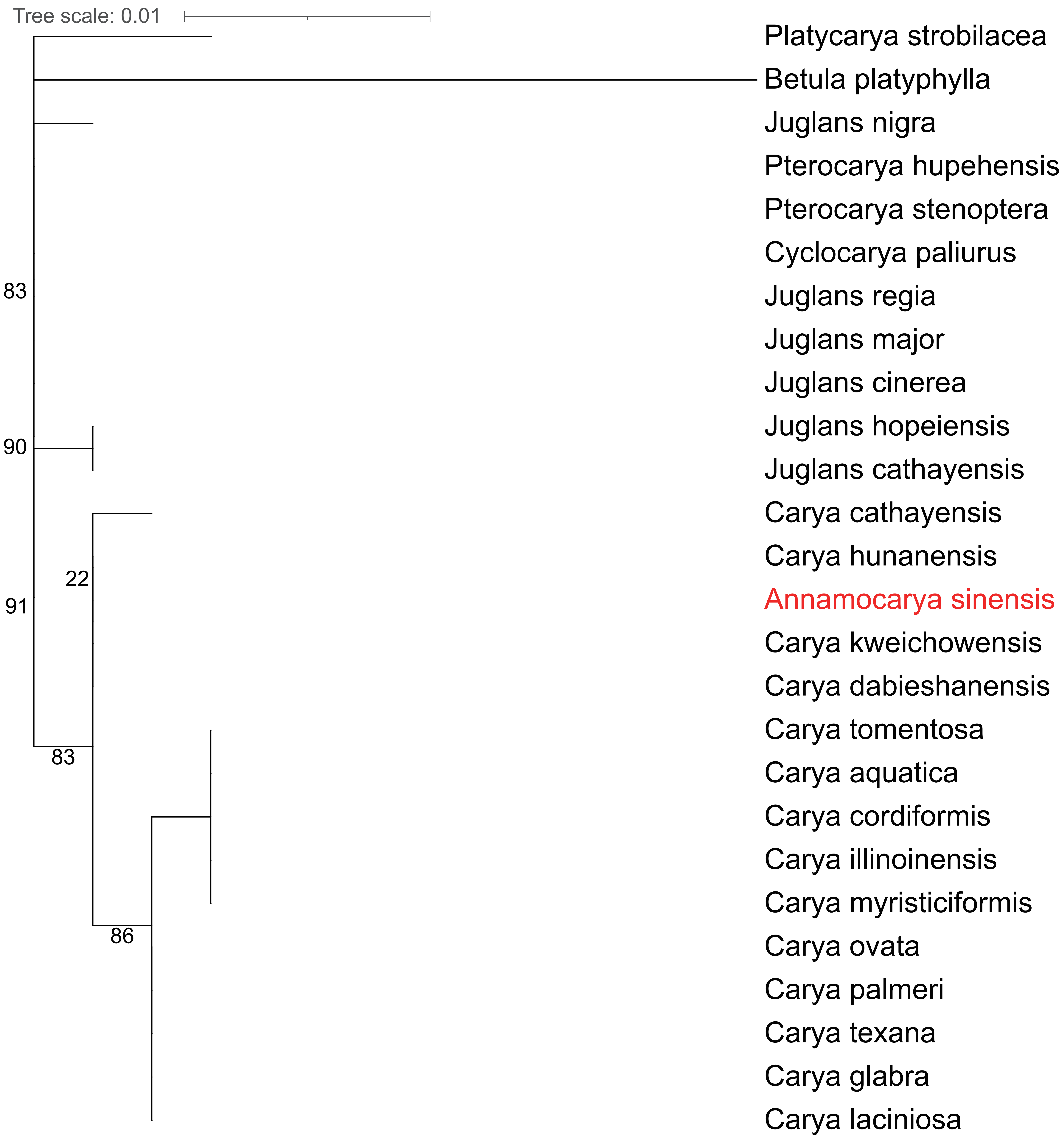

Figure S2A46. Phylogenetic tree constructed using rps3

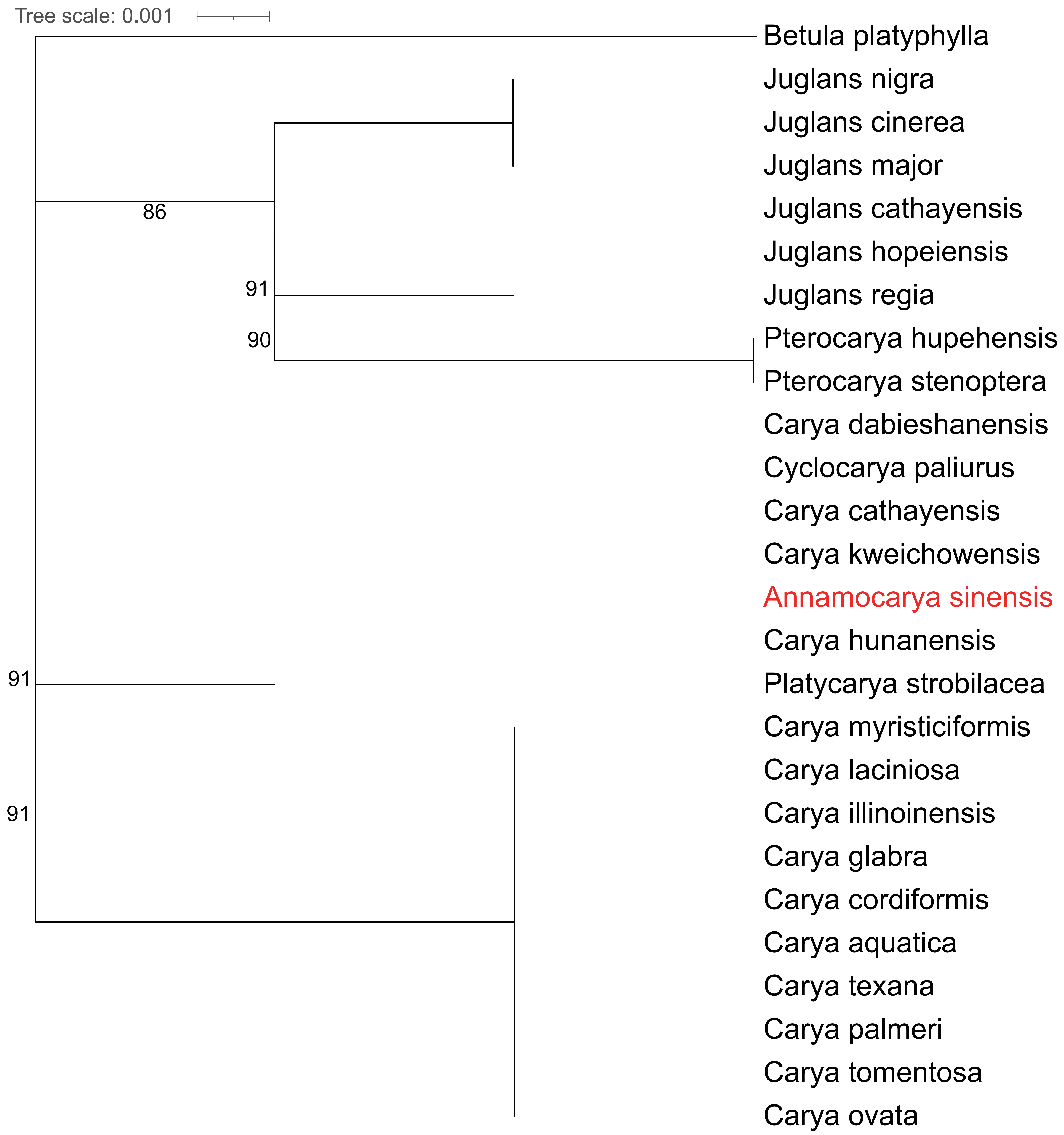

Figure S2A47. Phylogenetic tree constructed using rps4

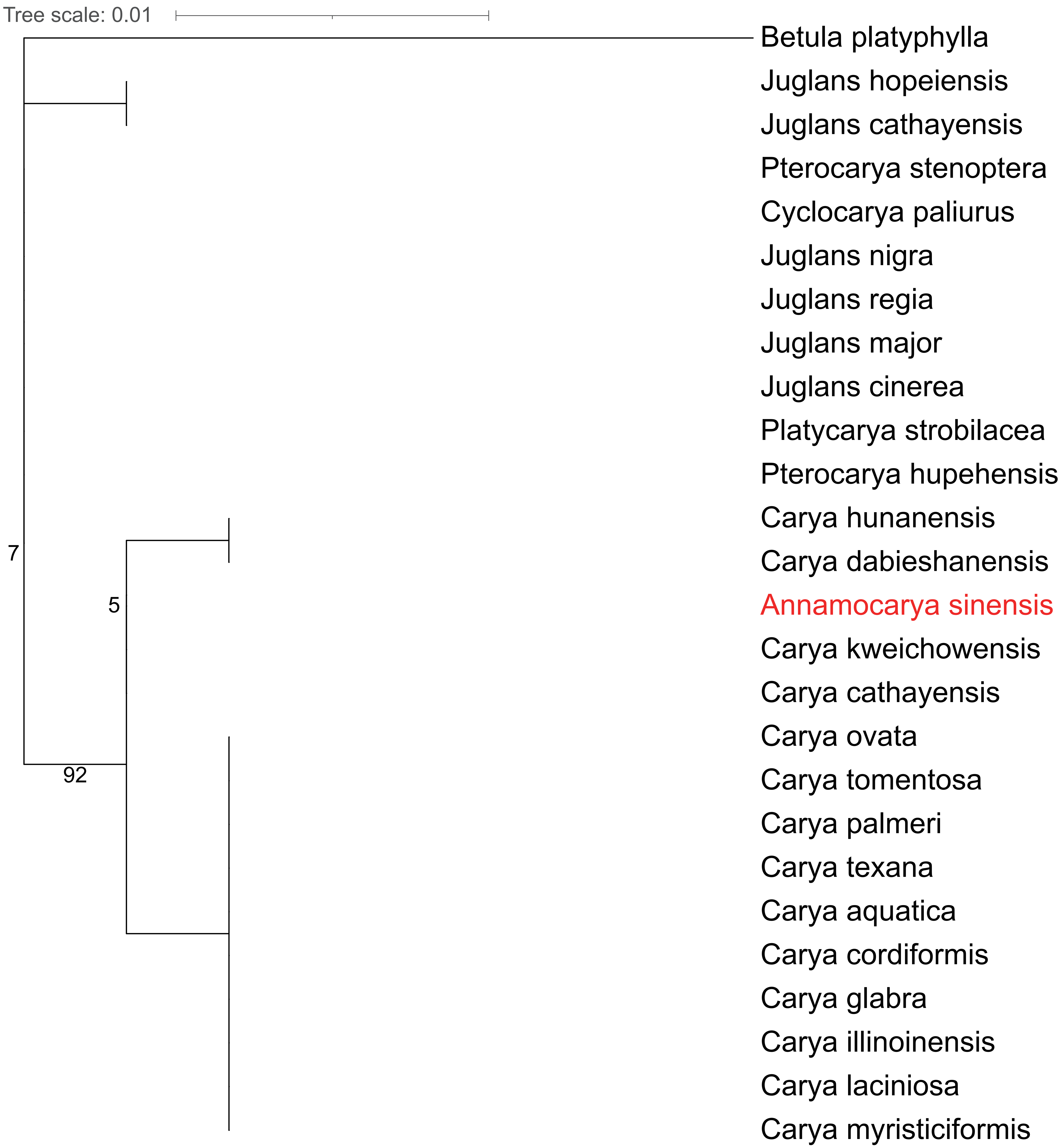

Figure S2A48. Phylogenetic tree constructed using rps8

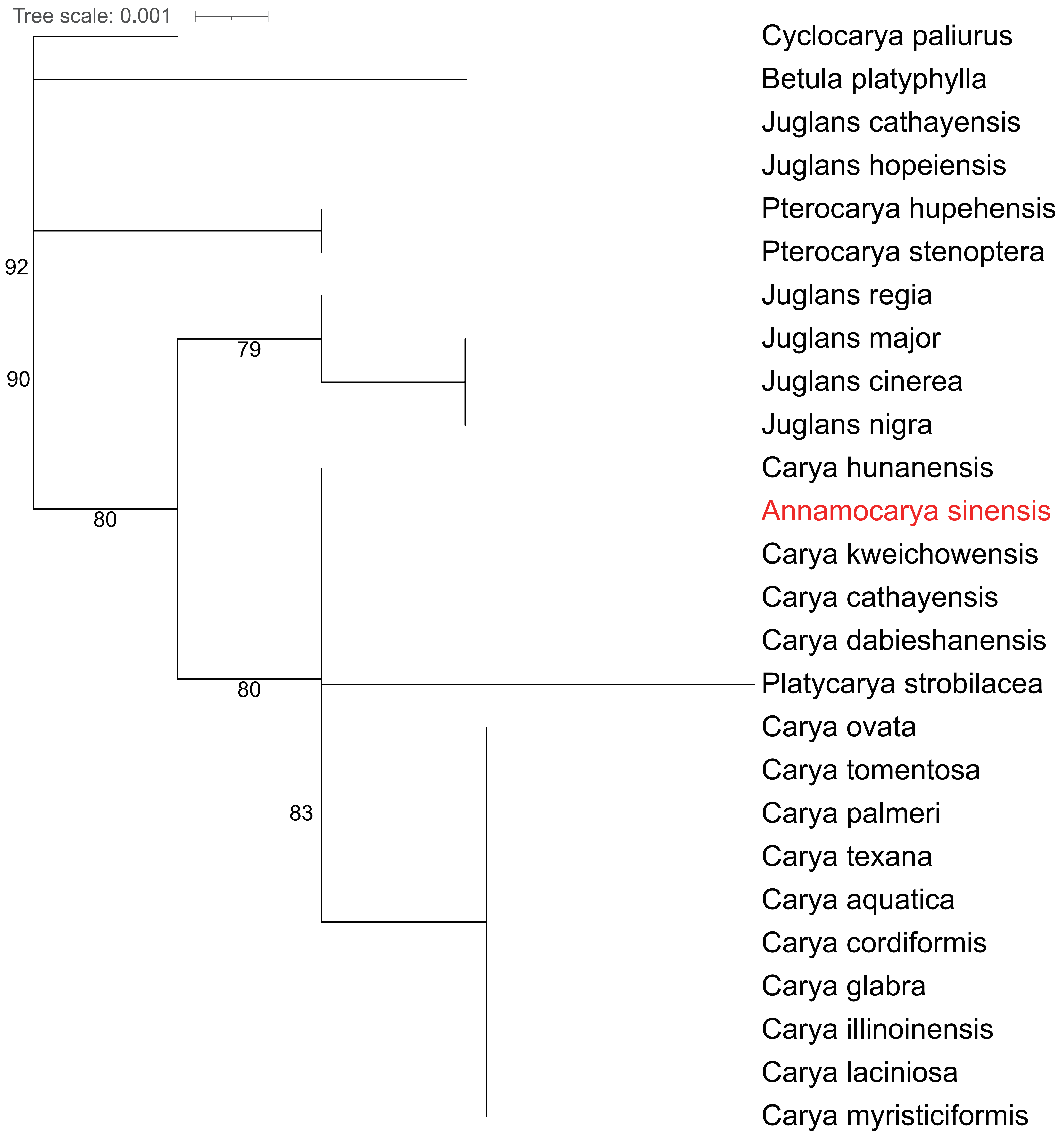

Figure S2A49. Phylogenetic tree constructed using ycf3

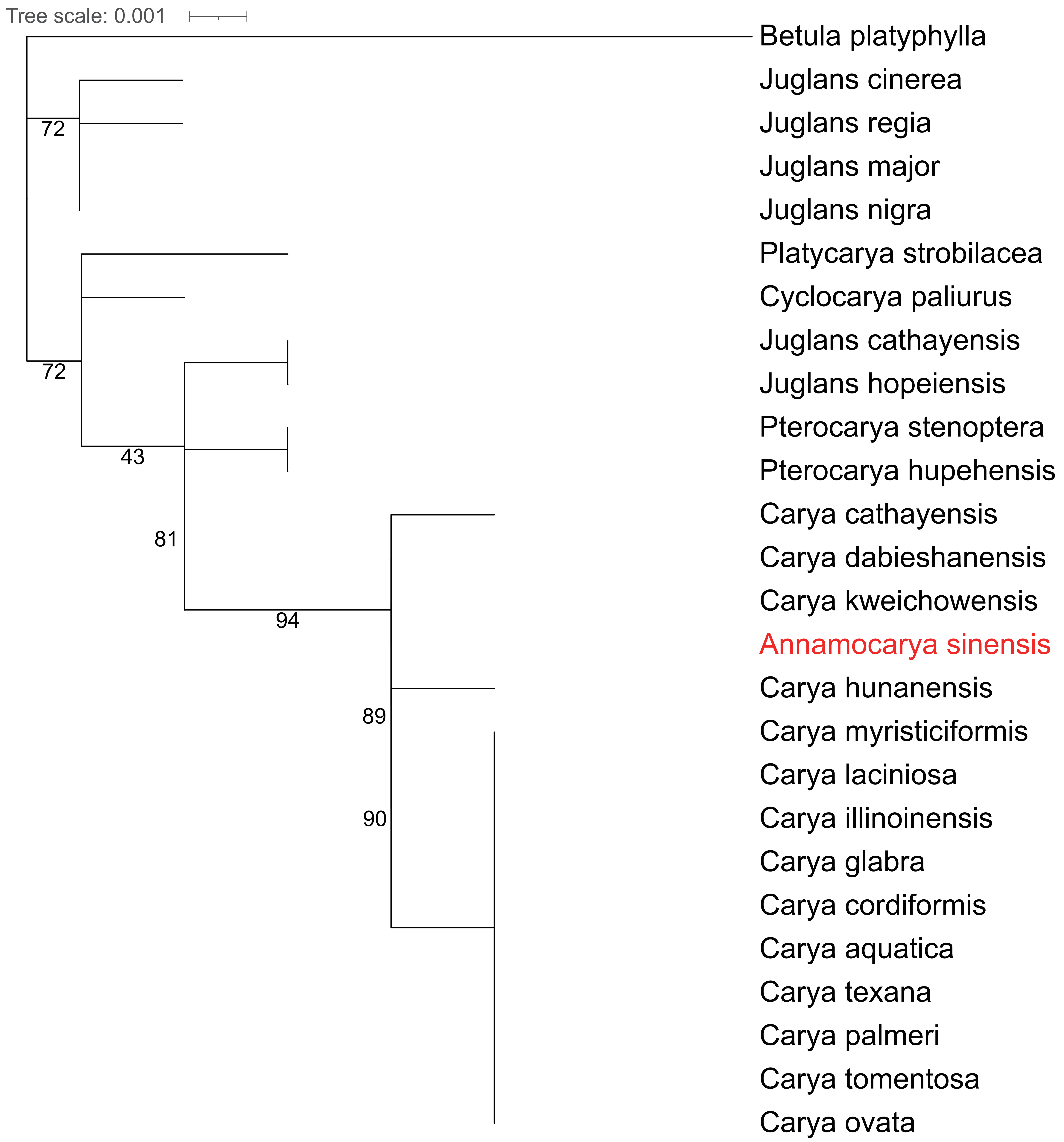

Figure S2A50. Phylogenetic tree constructed using ycf4
