# Supplementary material for: Reassessment of Annamocarya sinesis (Carya sinensis) Taxonomy through Concatenation and Coalescence Phylogenetic Analysis
Source: Plants (Basel). 2021 Dec 24;11(1):52. doi: 10.3390/plants11010052 (PMC8747223; doi:10.3390/plants11010052)
Supplement: Supplementary file 1 [file plants-11-00052-s001.zip › supplementary_materials_correction/Supplementary_Figure_1.pdf]

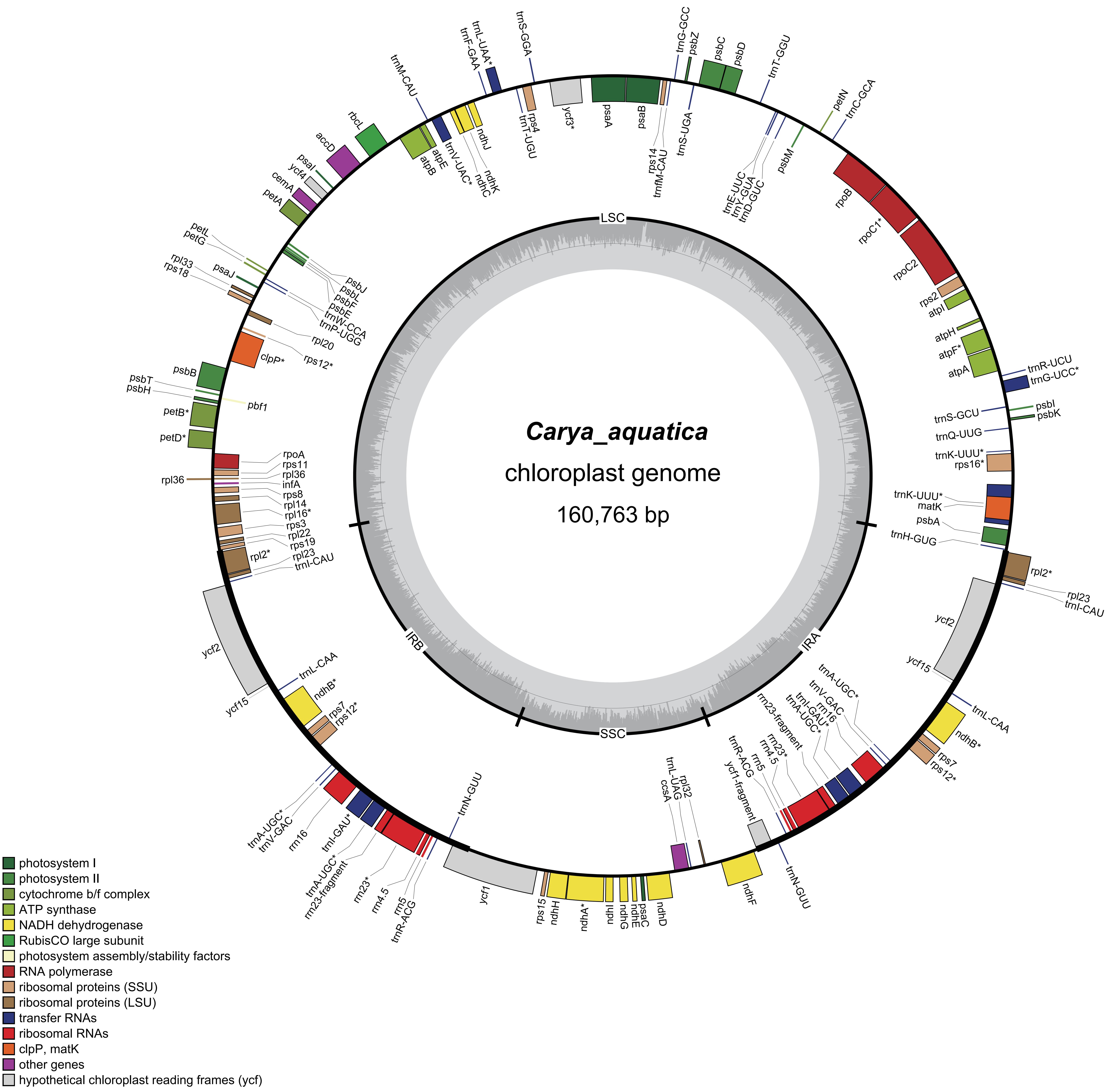

Figure S1A. Circular chloroplast genome map of *Carya aquatica*. The genes showninside and outside of the circle are transcribed in clockwise and counterclockwise directions,respectively. Genes from different functional groups are shown in different colors. The thick darklines in inner circle show the extent of the Inverted repeats (IRA and IRB) separating the LargeSingle-Copy (LSC) and the Small Single-Copy (SSC) regions. The gray ring represents the GC-content.

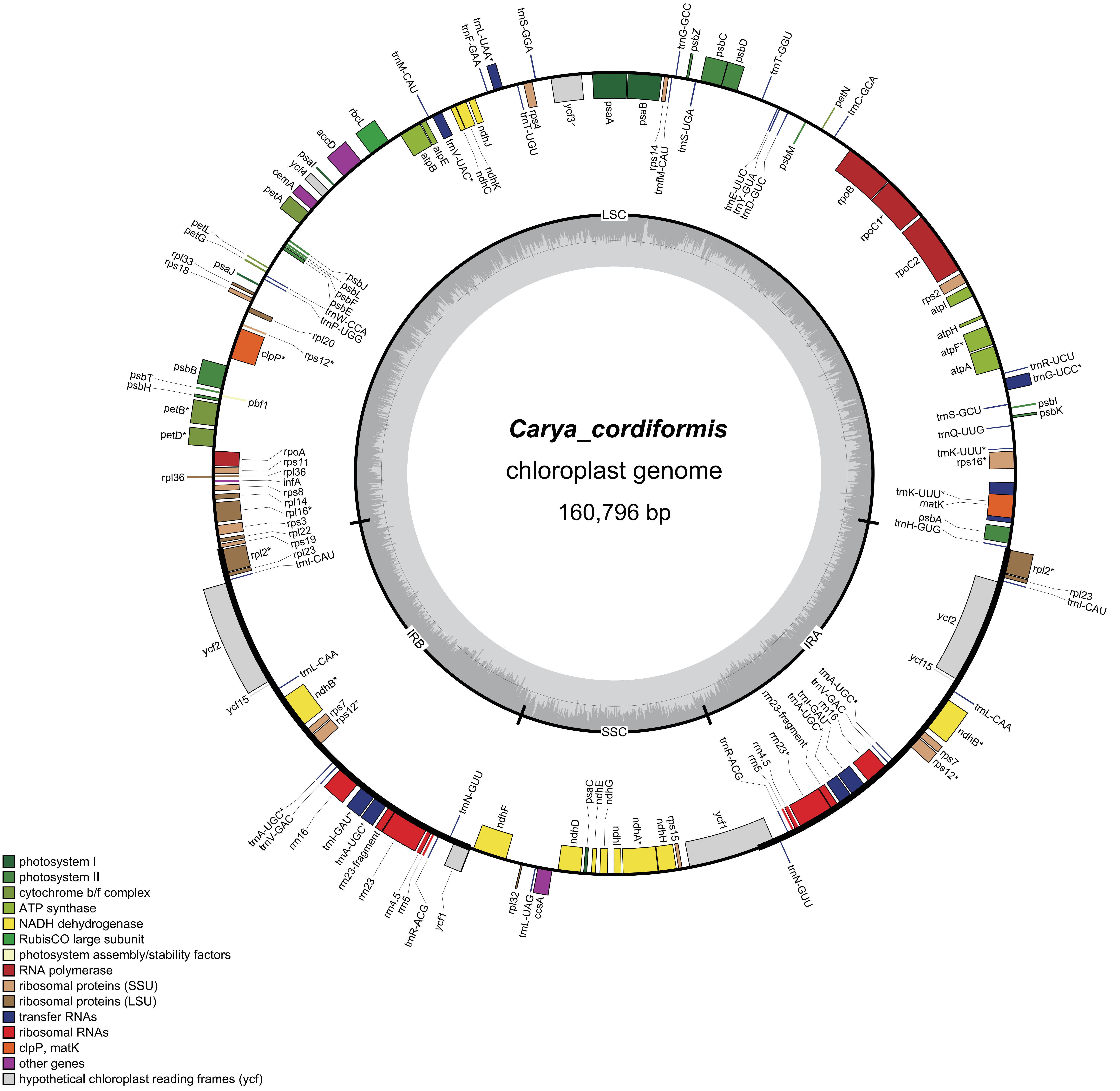

Figure S1B. Circular chloroplast genome map of *Carya cordiformis*. The genes showninside and outside of the circle are transcribed in clockwise and counterclockwise directions,respectively. Genes from different functional groups are shown in different colors. The thick darklines in inner circle show the extent of the Inverted repeats (IRA and IRB) separating the LargeSingle-Copy (LSC) and the Small Single-Copy (SSC) regions. The gray ring represents the GC-content.

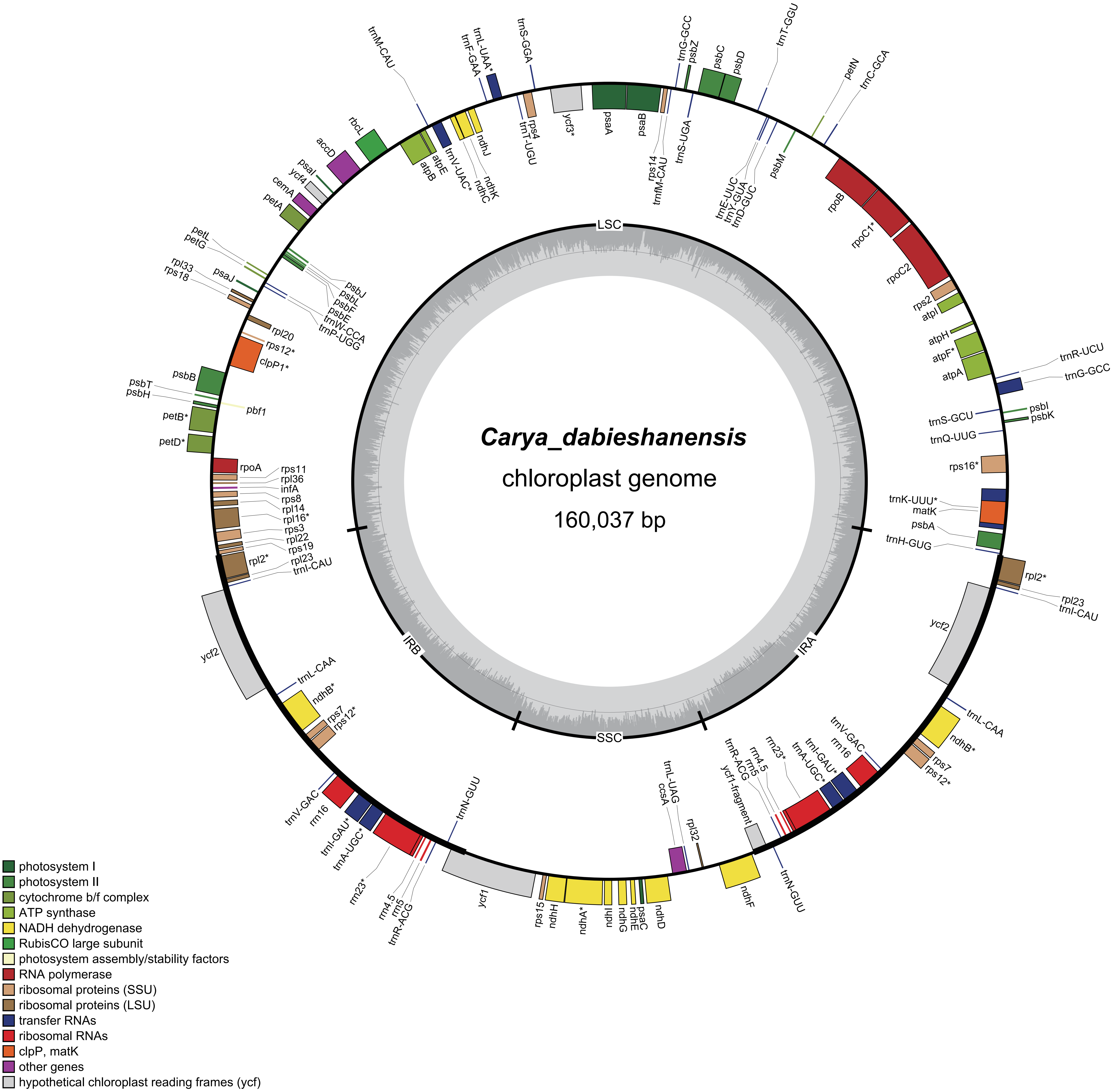

Figure S1C. Circular chloroplast genome map of *Carya dabieshanensis*. The genes shown inside and outside of the circle are transcribed in clockwise and counterclockwise directions,respectively. Genes from different functional groups are shown in different colors. The thick darklines in inner circle show the extent of the Inverted repeats (IRA and IRB) separating the LargeSingle-Copy (LSC) and the Small Single-Copy (SSC) regions. The gray ring represents the GC-content.

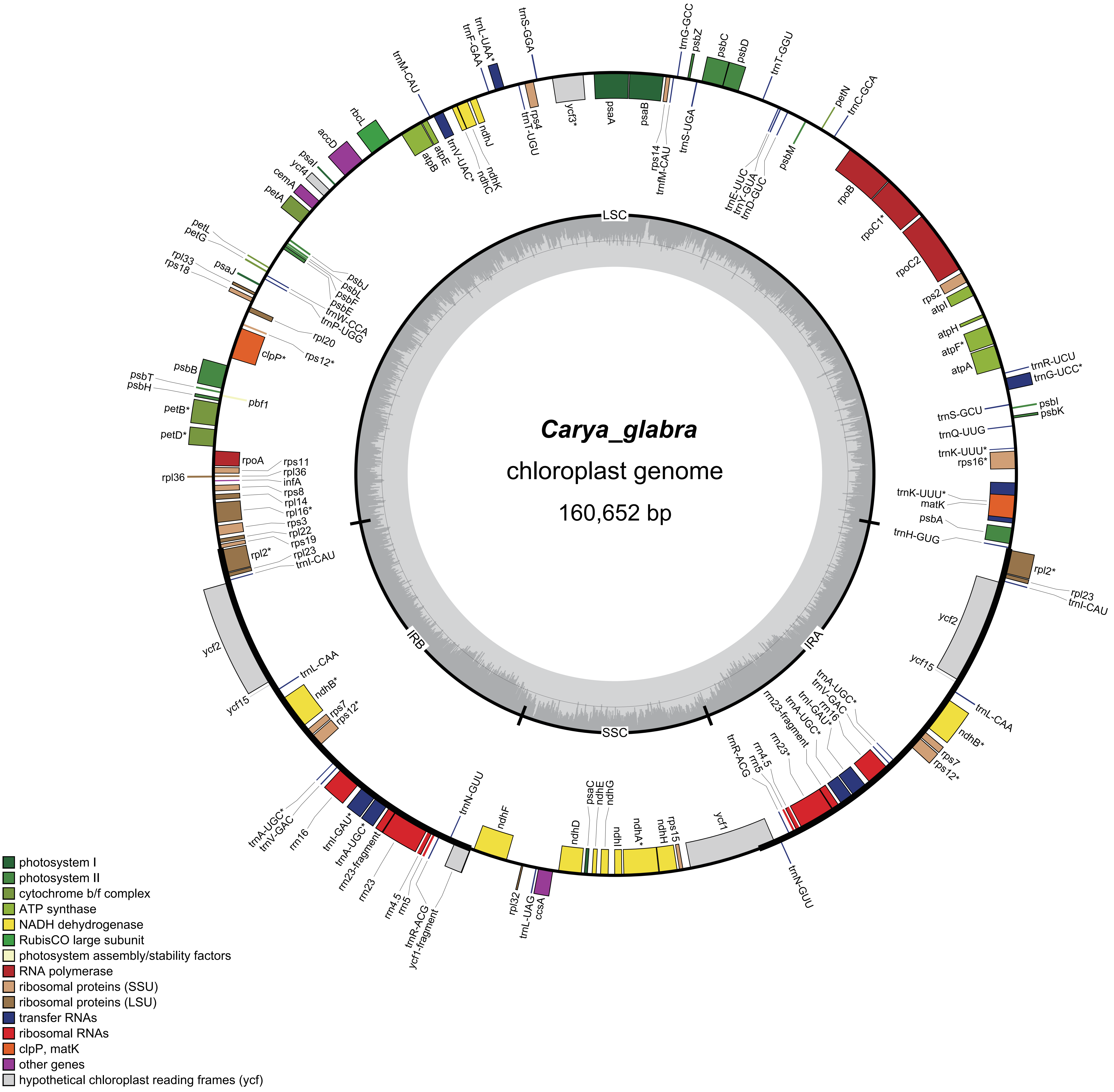

Figure S1D. Circular chloroplast genome map of *Carya glabra*. The genes showninside and outside of the circle are transcribed in clockwise and counterclockwise directions,respectively. Genes from different functional groups are shown in different colors. The thick darklines in inner circle show the extent of the Inverted repeats (IRA and IRB) separating the LargeSingle-Copy (LSC) and the Small Single-Copy (SSC) regions. The gray ring represents the GC-content.

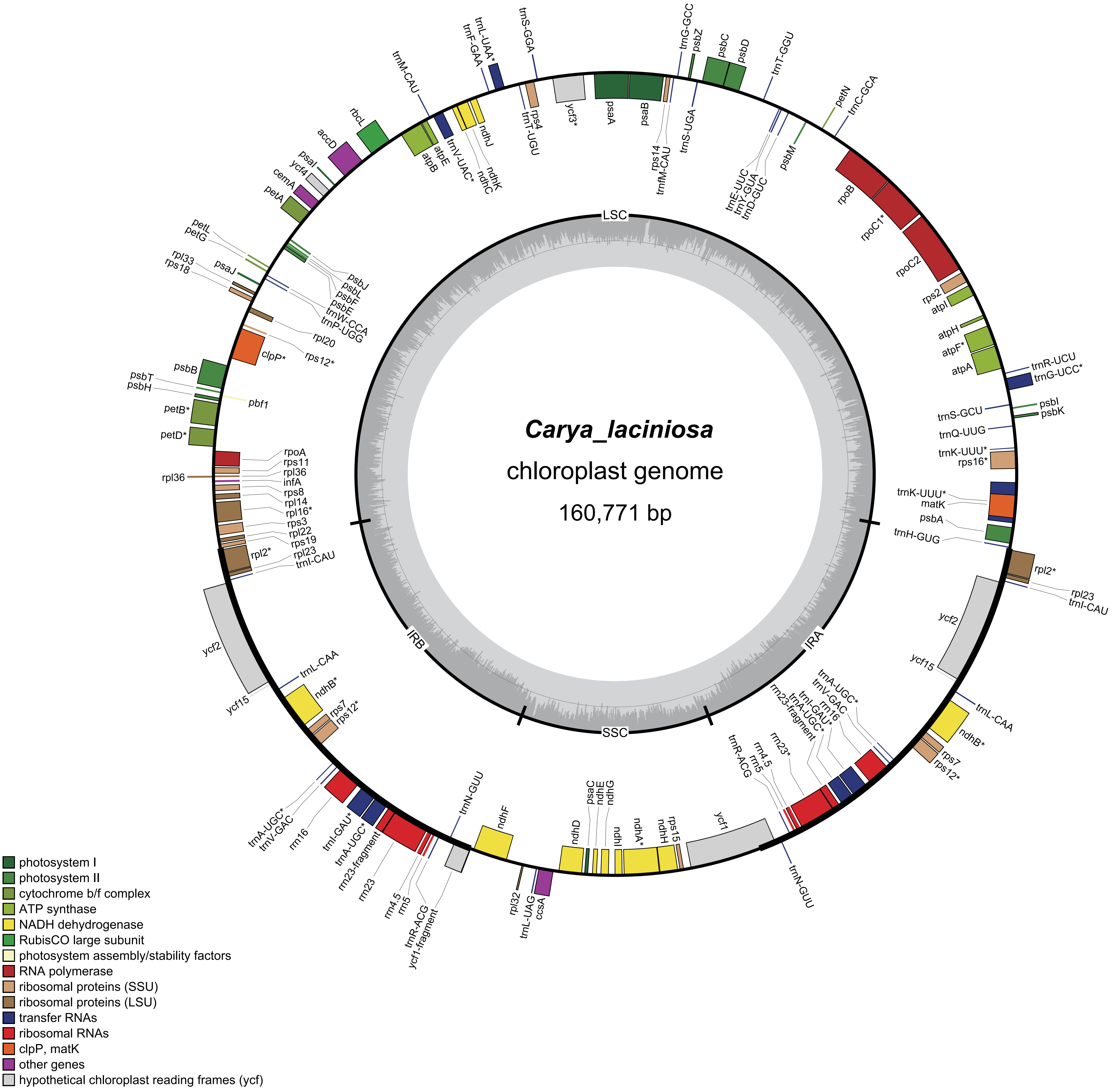

Figure S1E. Circular chloroplast genome map of *Carya laciniosa*. The genes showninside and outside of the circle are transcribed in clockwise and counterclockwise directions,respectively. Genes from different functional groups are shown in different colors. The thick darklines in inner circle show the extent of the Inverted repeats (IRA and IRB) separating the LargeSingle-Copy (LSC) and the Small Single-Copy (SSC) regions. The gray ring represents the GC-content.

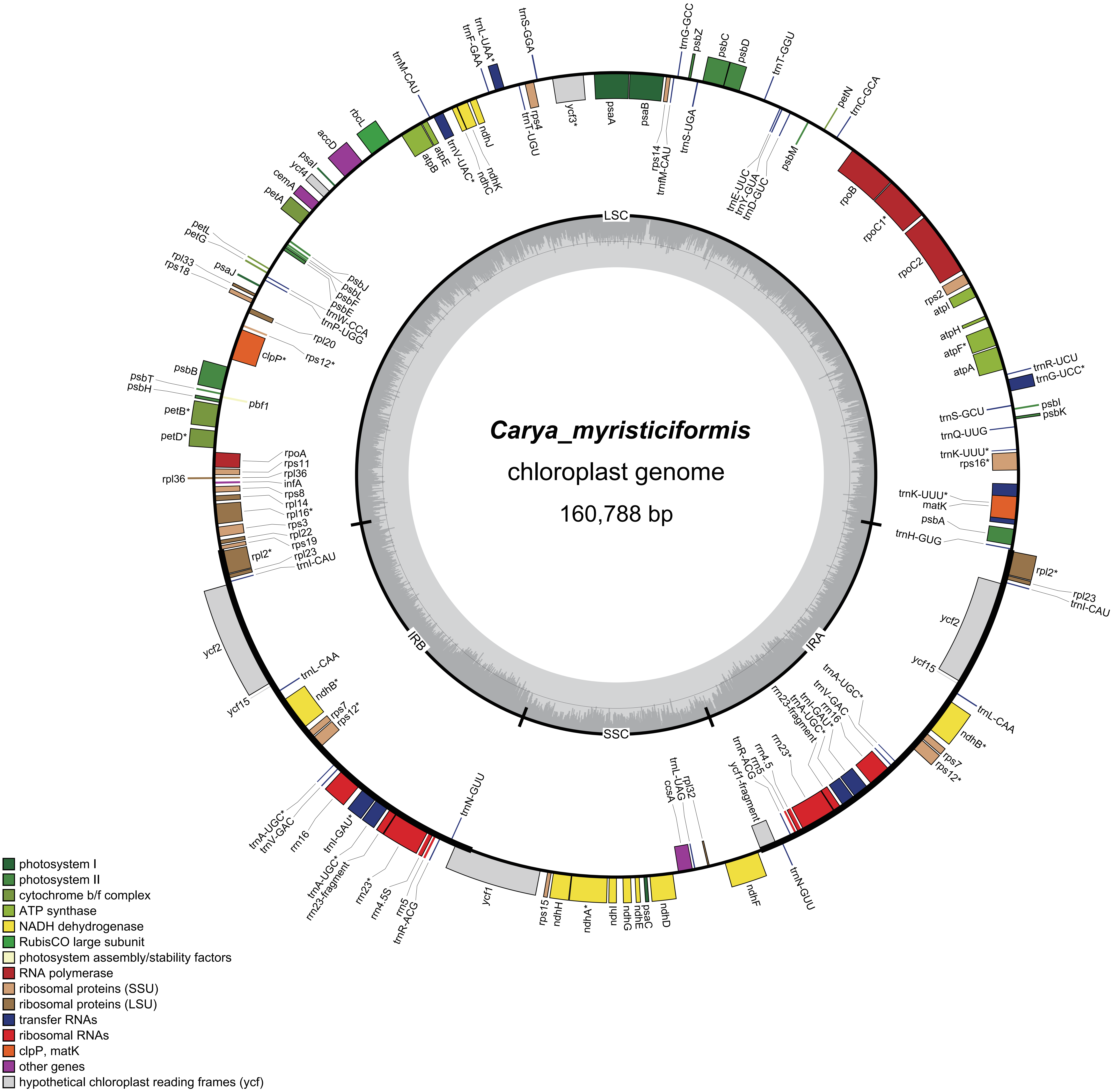

Figure S1F. Circular chloroplast genome map of *Carya myristiciformis*. The genes shownin-side and outside of the circle are transcribed in clockwise and counterclockwise directions,respectively. Genes from different functional groups are shown in different colors. The thick darklines in inner circle show the extent of the Inverted repeats (IRA and IRB) separating the LargeSingle-Copy (LSC) and the Small Single-Copy (SSC) regions. The gray ring represents the GC-content.

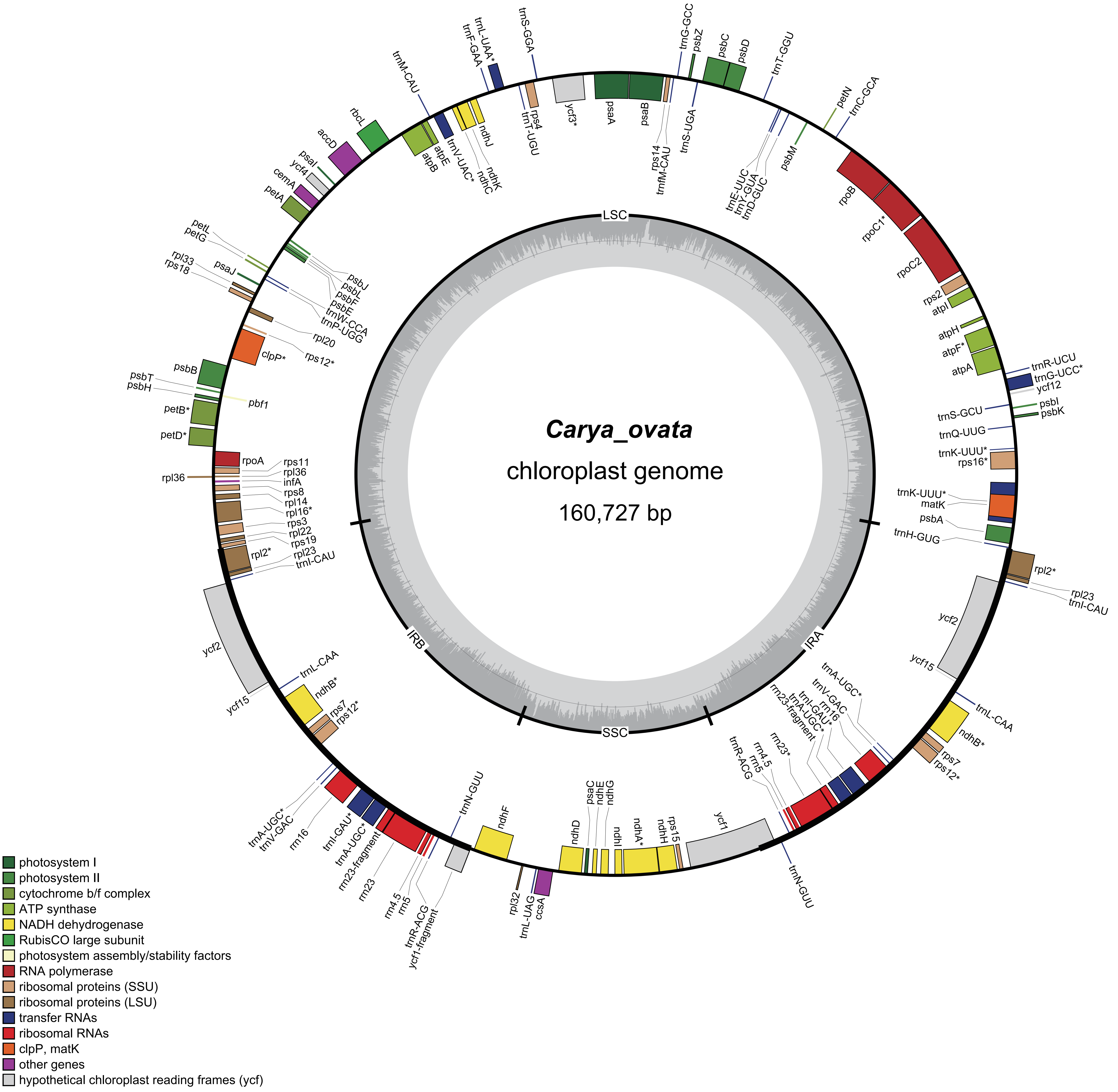

Figure S1G. Circular chloroplast genome map of *Carya ovata*. The genes showninside and outside of the circle are transcribed in clockwise and counterclockwise directions,respectively. Genes from different functional groups are shown in different colors. The thick darklines in inner circle show the extent of the Inverted repeats (IRA and IRB) separating the LargeSingle-Copy (LSC) and the Small Single-Copy (SSC) regions. The gray ring represents the GC-content.

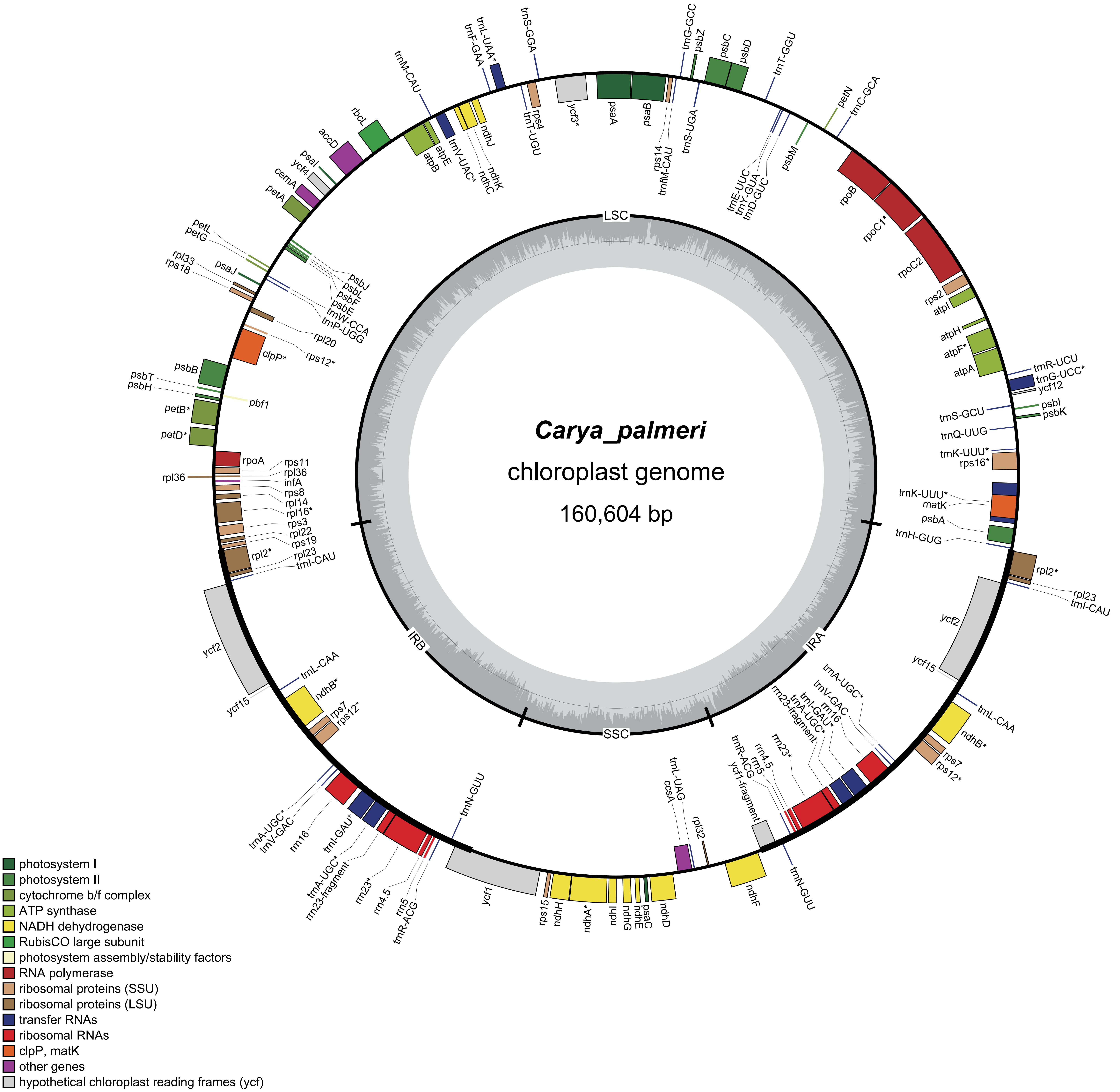

Figure S1H. Circular chloroplast genome map of *Carya palmeri*. The genes showninside and outside of the circle are transcribed in clockwise and counterclockwise directions,respectively. Genes from different functional groups are shown in different colors. The thick darklines in inner circle show the extent of the Inverted repeats (IRA and IRB) separating the LargeSingle-Copy (LSC) and the Small Single-Copy (SSC) regions. The gray ring represents the GC-content.

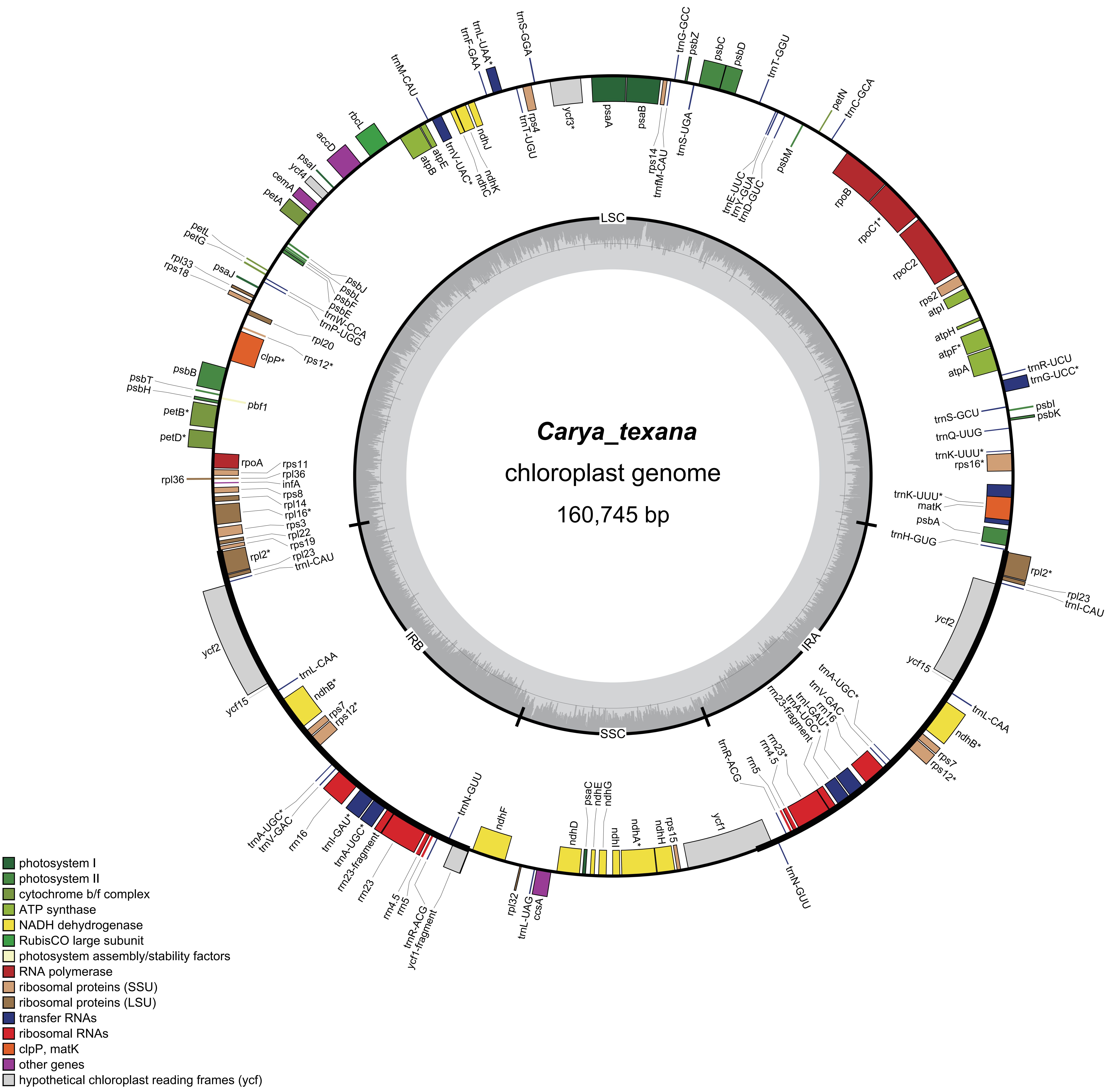

Figure S1I. Circular chloroplast genome map of *Carya texana*. The genes showninside and outside of the circle are transcribed in clockwise and counterclockwise directions,respectively. Genes from different functional groups are shown in different colors. The thick darklines in inner circle show the extent of the Inverted repeats (IRA and IRB) separating the LargeSingle-Copy (LSC) and the Small Single-Copy (SSC) regions. The gray ring represents the GC-content.

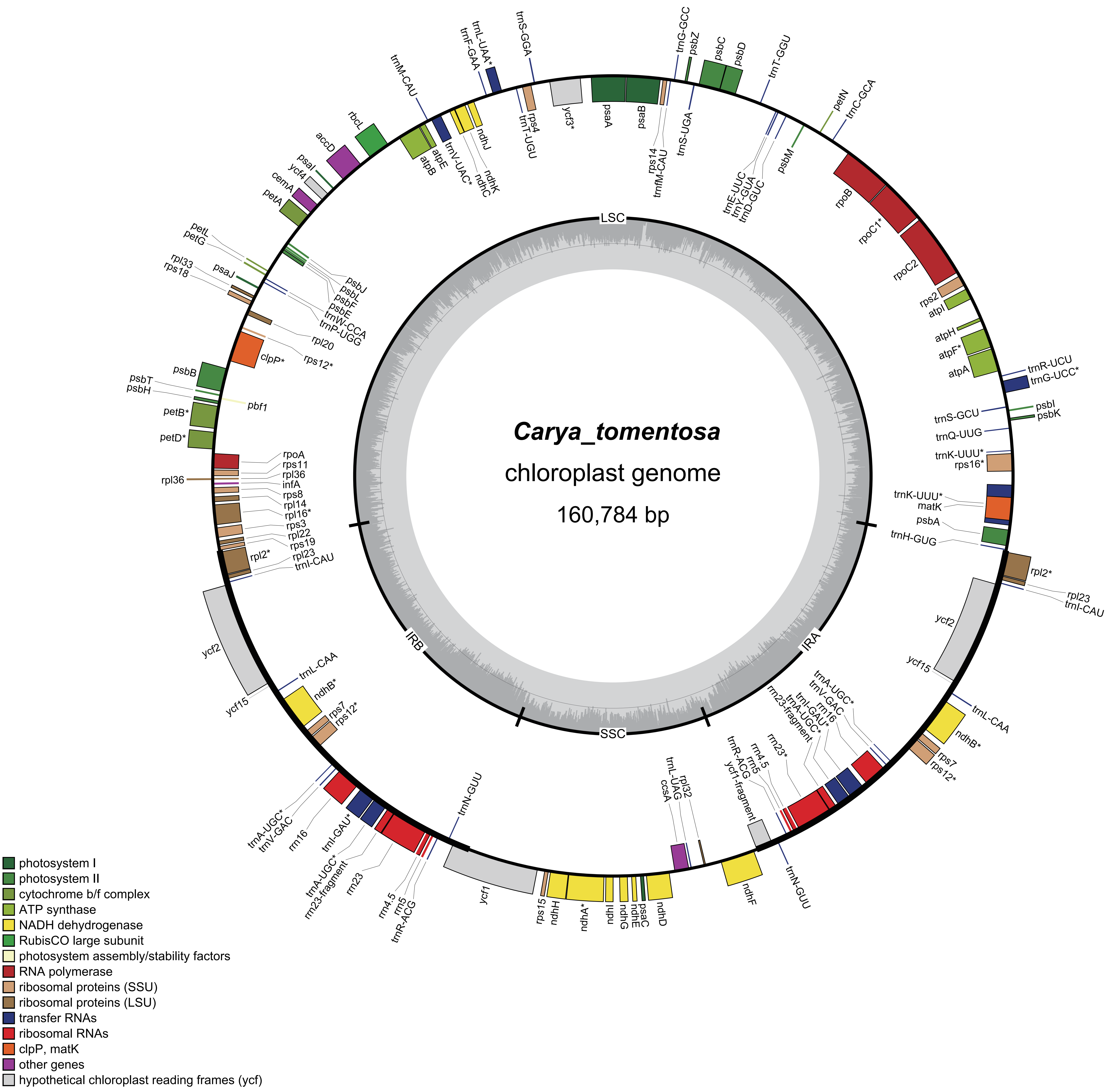

Figure S1J. Circular chloroplast genome map of *Carya tomentosa*. The genes showninside and outside of the circle are transcribed in clockwise and counterclockwise directions,respectively. Genes from different functional groups are shown in different colors. The thick darklines in inner circle show the extent of the Inverted repeats (IRA and IRB) separating the LargeSingle-Copy (LSC) and the Small Single-Copy (SSC) regions. The gray ring represents the GC-content.
