# Supplementary material for: Reassessment of Annamocarya sinesis (Carya sinensis) Taxonomy through Concatenation and Coalescence Phylogenetic Analysis
Source: Plants (Basel). 2021 Dec 24;11(1):52. doi: 10.3390/plants11010052 (PMC8747223; doi:10.3390/plants11010052)
Supplement: Supplementary file 1 [file plants-11-00052-s001.zip › supplementary_materials_correction/Supplementary_Figure_4.pdf]

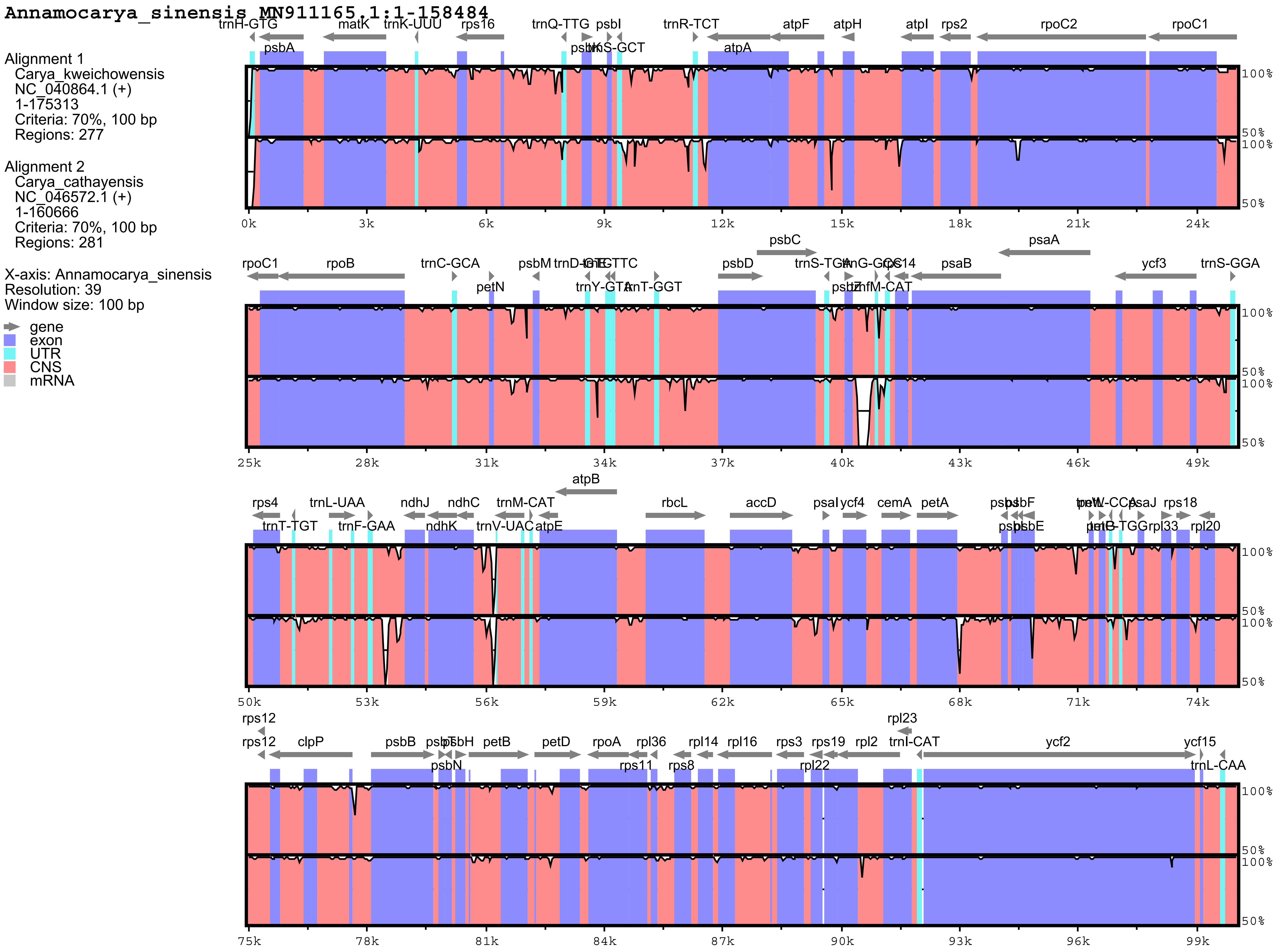

Annamocarya_sinensis MN911165.1:1-158484
Alignment 1
Carya_kweichowensis
NC_040864.1 (+)
1-175313
Criteria: 70%, 100 bp
Regions: 277
Alignment 2
Carya_cathayensis
NC_046572.1 (+)
1-160666
Criteria: 70%, 100 bp
Regions: 281
X-axis: Annamocarya_sinensis
Resolution: 39
Window size: 100 bp
gene
exon
UTR
CNS
mRNA
trnH-GTG
psbA
matK
trnK-UUU
rps16
trnQ-TTG
psbI
trnS-GCT
trnR-TCT
atpA
atpF
atpH
atpI
rps2
rpoC2
rpoC1
rpoB
trnC-GCA
petN
psbM
psbD
psbC
psaA
psaB
ycf3
trnS-GGA
trnY-GTA
trnT-GGT
rps14
trnfM-CAT
rps4
trnT-TGT
trnL-UAA
trnF-GAA
ndhJ
ndhK
ndhC
trnM-CAT
trnV-UAC
atpE
atpB
rbcL
accD
psaI
ycf4
cemA
petA
psbF
psbE
psaJ
rpl33
rps18
rpl20
rps12
clpP
psbB
psbN
psbH
petB
petD
rpoA
rpl36
rps11
rps8
rpl14
rpl16
rps3
rps19
rpl22
rpl2
rpl23
trnI-CAT
ycf2
ycf15
trnL-CAA
100%
50%
0k
3k
6k
9k
12k
15k
18k
21k
24k
25k
28k
31k
34k
37k
40k
43k
46k
49k
50k
53k
56k
59k
62k
65k
68k
71k
74k
75k
78k
81k
84k
87k
90k
93k
96k
99k

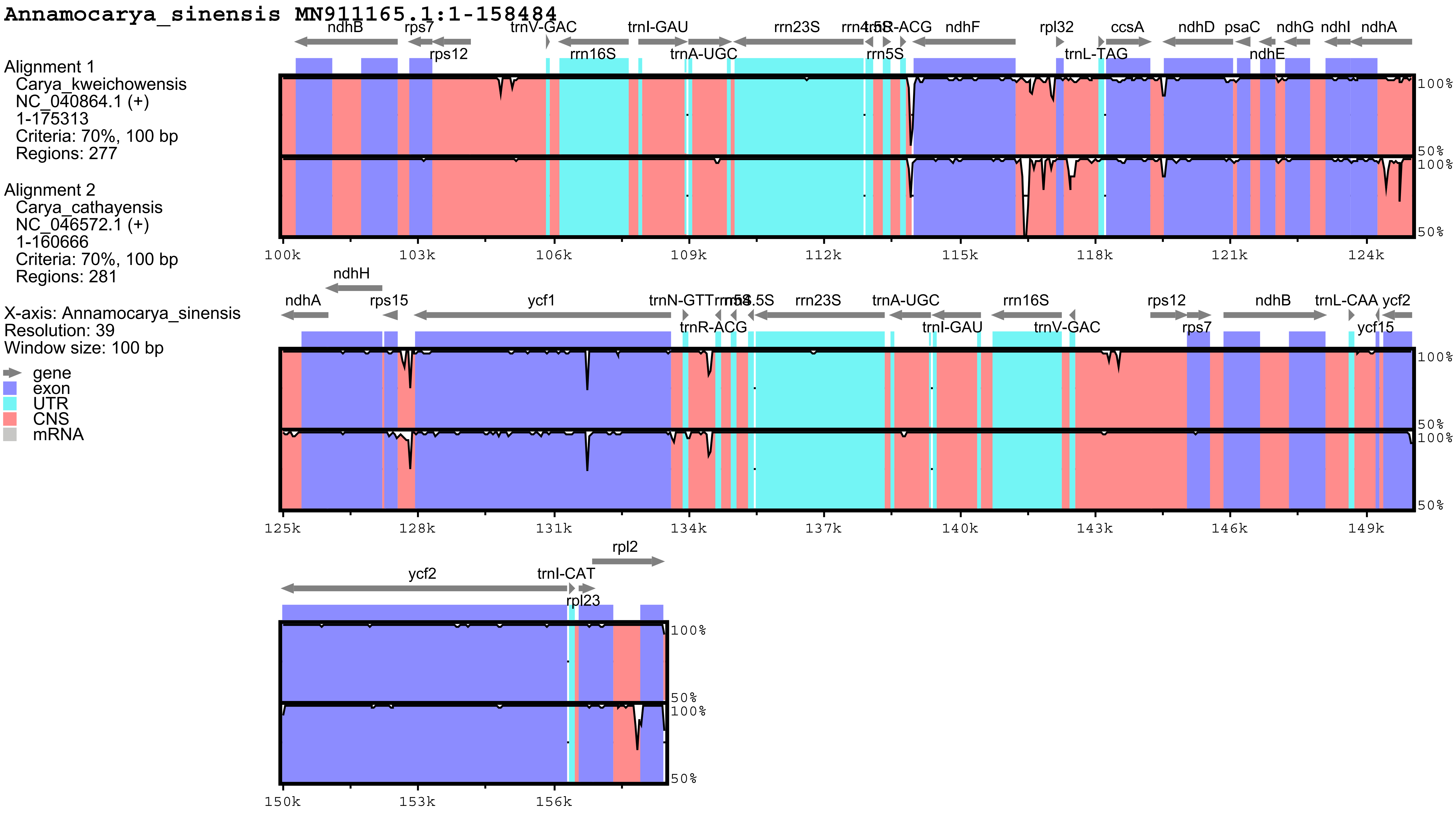

Figure S4: The alignment of chloroplast genomes of *A.sinensis*, *C.cathayensis* and *C.kweichowensis*. This result was constructed using mVISTA web tool (https://genome.lbl.gov/vista/index.shtml). The first raw (alignment 1) was the alignment between *C.kwichowensis* and *A.sinensis*. And the second raw (alignment 2) was the alignment between *C.cathayensis* and *A.sinensis*. The "valleys" in this graph indicated the non-conservation percentage between the two alignment genomes. The dark blue regions are exon. Untranslated regions (UTR) are marked in light blue, while conservative non-coding sequences (CNS) are marked in pink. The arrows signify the direction of the gene.
